# Supplementary material for: Associations of air pollution exposures in preconception and pregnancy with birth outcomes and infant neurocognitive development: analysis of the Complex Lipids in Mothers and Babies (CLIMB) prospective cohort in Chongqing, China
Source: BMJ Open. 2024 Jul 2;14(7):e082475. doi: 10.1136/bmjopen-2023-082475 (PMC11227797; doi:10.1136/bmjopen-2023-082475)
Supplement: Supplementary data [file bmjopen-2023-082475supp001.pdf]

Associations of air pollution exposures in preconception and pregnancy with birth outcomes and infant neurocognitive development: analysis of the Complex Lipids in Mothers and Babies (CLIMB) prospective cohort in Chongqing, China

Yingxin Chen[1,2], Tao Kuang[3], Ting Zhang[4], Yutong Samuel Cai[1,2,5], John Colombo[6], Alex Harper[7], Ting-Li Han[8], Yinyin Xia[9], John Gulliver[10], Anna L Hansell[1,2,5], Hua Zhang[8], Philip N Baker[7,8]

1: Centre for Environmental Health and Sustainability, University of Leicester, Leicester, UK

2: The National Institute of Health Research (NIHR) Health Protection Research Unit (HPRU) in Environmental Exposure and Health at the University of Leicester, Leicester, UK

3: Department of public health and management, Zunyi Medical and Pharmaceutical College, Zunyi, 563000, Guizhou, China

4. Stomatological Hospital of Chongqing Medical University, Chongqing, 401147, China

5: NIHR Leicester Biomedical Research Centre, Leicester General Hospital, Leicester, UK

6: Schiefelbusch Institute for Life Span Studies and Department of Psychology, University of Kansas, Lawrence, KS 66045, USA

7: College of Life Sciences, University of Leicester, Leicester, UK

8: Department of Obstetrics and Gynaecology, the First Affiliated Hospital of Chongqing Medical University, Chongqing, 400016, China

9: School of Public Health, Chongqing Medical University, Chongqing, 400016, China

10: Environmental and Exposure Sciences, Population Health Research Institute, St George's, University of London, London, UK

Correspondence to: Yinyin Xia. School of Public Health, Chongqing Medical University, Chongqing, 400016, China. Email: 100118@cqmu.edu.cn

Supplement

eTable 1 Mental Development Index (Chinese version)

智力量表

（⁎可偶尔观察到）

序号	月龄	条目	计分
1	0.1	对铃声反应	
2	0.1	抱起时安静	
3	0.1	对摇鼓声反应	
4	0.1	对尖声反应：（电灯开关）	
5	0.1	短暂地注视红环	
6	0.2	短暂地注视人	
7	0.4	稍长时间地注视红环	
8	0.5	眼的水平协调活动（红环）	
9	0.7	眼的水平向天活动（光）	
10	0.7	眼睛追随移动的人	
11	0.7	对说话声反应	
12	0.8	眼的垂直协调活动（光）	
13	0.9	发声一至两次	
14	1	眼的垂直协调活动（红环）	
15	1.2	眼的旋转协调活动（光）	
16	1.2	眼的旋转细条活动（光环）	
17	1.3	⁎自由环视周围	
18	1.5	社交笑：测试者谈话与微笑时	
19	1.6	眼转向红环	
20	1.6	眼转向光	
21	1.6	⁎发声至少四次	
22	1.7	期待性兴奋	
23	1.7	对面部的纸有反应	
24	1.9	能用视觉辨认母亲	
25	1.9	社交笑：测试者微笑与安静时	
26	2	⁎对测试者的微笑和说话有发声反应	
27	2.1	⁎用眼睛寻找声源（详细说明）	
28	2.2	⁎发出两种不同的声音	
29	2.2	对手的遮蔽眨眼	
30	2.2	对面孔的消失有反应	
31	2.4	注视方木	
32	2.6	从一物转看另一物	
33	2.6	眼睛追随铅笔	
34	2.7	对抱起有预感性的调节反应	
35	2.9	目光追随横过桌面的球	
36	2.9	头追随悬摆的环	

37	3.1	头追随逐渐消失的勺子	
38	3.2	操作红环	
39	3.3	简单地玩摇鼓	
40	3.4	※轻轻地抚摸桌沿	
41	3.4	※意识到陌生环境	
42	3.5	头转向铃声	
43	3.6	头转向摇鼓声	
44	3.6	※手碰手的玩耍	
45	3.6	将红环送入口中	
46	3.7	伸手够悬环	
47	3.8	看自己的手	
48	4.2	接近悬环（优势手）	
49	4.4	※发声时的姿态（描述）	
50	4.4	※主动抚摸桌沿	
51	4.4	接近镜像	
52	4.4	注意小糖丸	
53	4.6	伸手取方木	
54	4.7	喜欢嬉戏	
55	4.9	伸手时眼手协调	
56	4.9	拾起方木（优势手）	
57	5	保持两块方木	
58	5	持久地看红环	
59	5	头部跟着掉下的勺转动	
60	5	探索性地玩纸	
61	5	对镜像微笑	
62	5	坚持够东西	
63	5.1	在小床内重新找到摇鼓	
64	5.1	※辨别生人	
65	5.4	举起倒扣的茶杯	
66	5.5	※敲打玩耍	
67	5.5	探索性地玩细绳	
68	5.5	伸手取第二块方木	
69	5.6	※由一手向另一手传递物体	
70	5.8	※对产生响声感兴趣	
71	5.9	灵巧而直接地拾起方木	
72	6	※对镜像开玩笑	
73	6	用把柄举起茶杯	
74	6	寻找掉落的勺子	
75	6.1	牵拉细绳获取红环	
76	6.1	保留三块方木中的两块	
77	6.6	※发出四个不同的音节	
78	6.8	能配合玩游戏	
79	7	恰当地牵拉细绳获取红环	
80	7.1	玩摇铃，对细节感兴趣	
81	7.4	企图获得三块方木	

82	7.4	有目的地摇铃	
83	7.5	※选择性地倾听熟悉的词语	
84	8	※对 da-da 或类同词	
85	8.1	暴露玩具	
86	8.2	注意测试者的乱写	
87	8.3	将手指插入桩板洞中	
88	8.6	观看书中图画	
89	8.9	对他人的言语要求有反应	
90	9.1	拿起茶杯获得方木	
91	9.8	寻找盒子里面的东西	
92	10.3	遵照命令将方木放入茶杯（放入数）	
93	10.7	企图模仿乱写	
94	10.8	模仿用勺子搅拌	
95	10.9	遵照命令停止	
96	10.9	推动小汽车	
97	11	模仿地拍打哨娃	
98	11.1	※重复引入发笑的把戏	
99	11.2	解开裹着的方木	
100	11.2	将三块方木放入杯中	
101	11.4	※快速而不清的表达	
102	11.4	揭开兰盒子的盖	
103	11.5	翻开书页	
104	11.5	摇晃悬环的	
105	11.8	将骰子放入盒中（6 个）	
106	12	恰当地握持画笔	
107	12.2	模仿说单词（记录用过的词）	
108	12.4	重复地插一根桩钉	
109	12.5	用手势表达想要的东西	
110	12.9	自动乱写	
111	12.9	能说两个词	
112	13	搭两层塔	
113	13.1	出示鞋子或其他衣服或自己的玩具	
114	13.2	从瓶中移出小糖丸	
115	13.3	掺九块方木放入杯中	
116	14.3	※盖上圆盒	
117	14.4	兰色模板：放置一个圆形模块	
118	14.8	用棍子够取玩具	
119	15.4	搭三层塔	
120	15.7	在 70 秒钟内插完桩钉	
121	16.1	指出娃娃身体的各部分：三个部位以上	
122	16.3	粉红模板：放置圆形模块	
123	16.6	兰色模板：放置两个圆形模块	
124	17.2	用笔模仿画一划	
125	17.5	在 42 秒钟内插完桩钉	
126	17.6	说出一物名	

127	17.7	对娃娃执行指令（在通过部位打钩：椅、杯、鼻）	
128	18.1	用语言表达要求	
129	18.6	不用于一划的乱写?	
130	18.8	兰色模板：放置两个圆快和方块	
131	18.8	指出三幅画	
132	19.1	能说两个单词的句子	
133	19.2	说出一副画名	
134	19.2	说出两幅画名	
135	19.3	找出两物	
136	19.8	在 30 秒钟内插完桩钉	
137	20.4	粉红模板：完成	
138	20.4	搭六层塔	
139	20.5	兰色模板，放置六个模块	
140	21	指出五副画	
141	21.1	说出三物名	
142	21.2	勉强合格地安装破娃娃	
143	21.2	区别两物：杯、盘、盒	
144	22.8	辨认钟表：第四张图 1，2，3，4，5	
145	22.9	说出三幅画名	
146	23.8	粉红模板（反转）	
147	24.3	近似地安装破娃娃	
148	24.6	区别三物：杯、盘、盒	
149	24.7	兰色模板，在 150 秒钟内完成	
150	25	搭八层塔	
151	25.1	指出七副画	
152	25.1	用方木搭火车	
153	25.7	说出五副画名	
154	26.3	模仿笔划：垂直线和水平线	
155	27.1	辨认钟表：第 2 张图	
156	27.6	理解两个方位词	
157	28	在 22 秒钟内插完桩钉	
158	28.5	兰色模板：90 秒钟内完成	
159	29.5	折纸	
160	29.6	兰色模板：60 秒钟内完成	
161	30+	正确安装破娃娃	
162	30+	“—”的概念	
163	30+	理解三个方位词	

eTable 2 Psychomotor Development Index (Chinese version)

运动量表

（⁎可偶尔观察到，△可在施测智力量表时观察到）

序号	月龄	条目	计分
1	0.1	抱起靠肩时抬头	
2	0.1	抱起靠肩时调整姿势	
3	0.1	侧头	
4	0.1	爬起	
5	0.8	△保留红环	
6	0.8	⁎伸臂玩耍	
7	0.8	⁎踢腿玩耍	
8	0.8	头起竖起：垂直位	
9	1.6	头部稳定地竖起	
10	1.7	抬头（背悬位）	
11	1.8	由侧卧转向仰卧	
12	2.2	在俯卧位时用双臂撑起自己	
13	2.2	支撑下坐起	
14	2.5	保持头部稳定	
15	2.6	⁎双手张开占优势	
16	3.3	头平衡	
17	3.4	⁎尺侧一手掌抓握方木	
18	3.5	轻度支撑坐位	
19	4.3	⁎由仰卧转向侧卧	
20	4.7	努力想坐起	
21	5.0	部分的拇指相对（桡侧一手掌）拾起方木	
22	5.1	独坐片刻	
23	5.1	⁎单手抽取	
24	5.2	⁎转腕	
25	5.2	牵拉坐起	
26	5.6	△试图获取小糖丸	
27	5.7	独立 30 秒钟或以上	
28	5.8	由仰卧转向俯卧	
29	6.2	稳定地独坐	
30	6.5	独坐时协调好	
31	6.6	⁎舀起小糖丸	
32	6.6	△完全的拇指相对拾起方木	
33	7	早期跨步运动	
34	7.5	牵拉站起	
35	7.6	⁎不完全的拇指相对抓糖丸	
36	7.6	走路之前的行进方式（俯卧、手膝、手足、其他）	
37	8.3	使两个勺子或方木在中线相碰	
38	8.5	跨步运动	

39	8.6	自己坐起	
40	8.6	借助家具站起	
41	8.9	精细地抓糖丸（灵巧地钳夹）	
42	9.6	拍手（中线技巧）	
43	9.8	坐下	
44	10	扶助下行走	
45	11.1	独站	
46	12	投球	
47	12.1	独走	
48	12.4	起立 I	
49	13.2	扶助下右足独站	
50	13.7	扶助下左足独站	
51	14.1	侧身走	
52	14.5	扶助上楼梯	
53	14.7	倒退走	
54	15.1	扶助下楼梯	
55	17.6	试图站在行木上	
56	18.7	左足独站	
57	19.3	单足踏在行木上走	
58	19.9	起立 II	
59	20.1	右足独站	
60	21.1	走直线：大致方向	
61	23.1	行木：双足站立	
62	24	踮脚走几步	
63	24.3	独自上楼梯：双足	
64	24.4	双足跳离地面	
65	25.3	独自下楼梯	
66	25.6	行木：企图跨步	
67	25.6	倒行两米半	
68	25.7	自第一级台阶下跳下	
69	29.2	自第二级台阶下跳下	
70	29.8	踮脚走两米半	
71	29.9	跳远：10 至 35cm（记录距离）	
72	30+	起立：III	
73	30+	上楼梯：双足交替向前	
74	30+	行木：交替步伐走部分路程	
75	30+	保持双足走在直线上（两米半）	
76	30+	跳远：35cm 至 60cm	
77	30+	跳过：5cm 高的绳子	
78	30+	跳远：60cm 至 85cm	
79	30+	独脚跳两次以上	
80	30+	下楼梯：双足交替向前	
81	30+	跳过 20cm 高的绳子	

eTable 3 Distributions of $PM_{2.5}$ and NO_2 exposure level in 90 days prior to conception, each trimester (T1, T2, and T3) and combined across whole pregnancy period (WP) (n = 1,174)

	Estimated exposure (μg/m³)						
	N	Minimum	25th percentile	Mean ± SD	Median ± IQR	75th percentile	Maximum
Estimated exposure to $PM_{2.5}$							
90 days prior to conception	1,174	38.17	44.00	52.91 ± 10.99	48.43 ± 18.07	62.06	80.53
First trimester	1,174	37.26	43.77	52.07 ± 10.98	47.26 ± 17.31	61.08	82.41
Second trimester	1,174	38.46	47.57	58.64 ± 12.21	57.97 ± 19.62	67.19	90.02
Third trimester	1,174	37.03	47.25	61.83 ± 16.04	58.82 ± 28.7	75.95	96.48
Total pregnancy	1,174	46.69	54.85	57.48 ± 3.97	57.31 ± 5.76	60.61	66.98
Estimated exposure to NO_2							
90 days prior to conception	1,174	25.86	45.49	49.59 ± 6.34	49.94 ± 8.27	53.76	70.48
First trimester	1,174	20.81	44.60	48.8 ± 6.43	48.92 ± 8.51	53.10	69.31
Second trimester	1,174	28.93	47.18	50.98 ± 6.23	51.20 ± 7.72	54.90	70.42
Third trimester	1,174	20.57	47.20	51.79 ± 6.78	52.45 ± 9.47	56.67	75.12
Total pregnancy	1,174	27.50	47.89	50.52 ± 5.08	50.46 ± 5.51	53.40	67.53

eTable 4 Pearson's correlations of $PM_{2.5}$ and NO_2 between each of the five different pregnancy time periods (N = 1,174)

Estimated exposure to		$PM_{2.5}$					NO_2				
		90 days prior to conception	First trimester	Second trimester	Third trimester	Total pregnancy	90 days prior to conception	First trimester	Second trimester	Third trimester	Total pregnancy
**$PM_{2.5}$**	90 days prior to conception	1									
	First trimester	-0.065	1								
	Second trimester	-0.779	-0.2012	1							
	Third trimester	0.288	-0.7613	-0.1688	1						
	Total pregnancy	-0.534	-0.2709	0.6838	0.3858	1					
**NO_2**	90 days prior to conception	0.6383	0.0684	-0.4588	0.3714	0.0376	1				
	First trimester	0.1537	0.6352	-0.0159	-0.4927	-0.0633	0.5545	1			
	Second trimester	-0.431	0.0714	0.6269	-0.0133	0.7251	0.3345	0.5399	1		
	Third trimester	0.3027	-0.5213	0.0528	0.6817	0.4432	0.7149	0.2159	0.5145	1	
	Total pregnancy	0.0057	0.0781	0.2862	0.0737	0.4779	0.6786	0.7435	0.8755	0.7331	1

eTable 5 Associations between $PM_{2.5}$ and NO_2 exposure in different pregnancy periods and adverse birth outcomes (unadjusted models)

Per IQR increase in		Mean difference		Odd ratios			
		Birth weight, grams (95% CI) (N=941)	Birth length, cm (95% CI) (N=927)	PTB (case: 33) (95% CI) (N=945)	LBW (case: 30) (95% CI) (N=945)	LGA (case: 108) (95% CI) (N=945)	SGA (case: 84) (95% CI) (N=945)
Estimated exposure to $PM_{2.5}$	90 days prior to conception	9.28 (-31.26, 49.83)	-0.09 (-0.27, 0.09)	0.98 (0.56, 1.74)	1.35 (0.77, 2.36)	1.2 (0.87, 1.64)	0.98 (0.68, 1.41)
	First trimester	21.95 (-16.90, 60.80)	0.14 (-0.03, 0.31)	0.98 (0.57, 1.70)	1 (0.57, 1.77)	0.97 (0.71, 1.33)	0.78 (0.54, 1.13)
	Second trimester	-18.21 (-57.78, 21.37)	0.04 (-0.13, 0.21)	0.85 (0.48, 1.50)	0.61 (0.32, 1.15)	0.92 (0.67, 1.27)	1.33 (0.94, 1.89)
	Third trimester	-37.38 (-81.43, 6.68)	**-0.32 (-0.51, -0.13)**	1.35 (0.74, 2.47)	1.51 (0.80, 2.85)	1.08 (0.76, 1.54)	1.12 (0.76, 1.66)
	Total pregnancy	-20.02 (-55.69, 15.65)	-0.1 (-0.26, 0.05)	0.81 (0.49, 1.33)	0.69 (0.41, 1.16)	1 (0.75, 1.34)	1.2 (0.87, 1.66)
Estimated exposure to NO_2	90 days prior to conception	-13.23 (-45.50, 19.03)	-0.12 (-0.26, 0.02)	1.2 (0.76, 1.89)	1.62 (0.99, 2.65)	1.21 (0.93, 1.57)	1.24 (0.92, 1.66)
	First trimester	0.3 (-32.36, 32.96)	0.08 (-0.06, 0.22)	1.01 (0.64, 1.60)	1.15 (0.71, 1.86)	1.17 (0.90, 1.52)	1.27 (0.94, 1.71)
	Second trimester	-22.85 (-53.39, 7.70)	-0.04 (-0.17, 0.09)	1.11 (0.72, 1.70)	1.08 (0.69, 1.69)	1.06 (0.83, 1.36)	**1.46 (1.10, 1.93)**
	Third trimester	-32.72 (-67.16, 1.72)	**-0.16 (-0.32, -0.01)**	1.13 (0.69, 1.85)	1.35 (0.80, 2.28)	1.24 (0.94, 1.65)	**1.58 (1.14, 2.18)**
	Total pregnancy	-16.58 (-43.35, 10.20)	-0.03 (-0.15, 0.09)	1.03 (0.71, 1.50)	1.13 (0.76, 1.69)	1.16 (0.93, 1.44)	**1.44 (1.13, 1.85)**

All significant findings in the table are bold.

eTable 6 Associations between $PM_{2.5}$ and NO_2 exposure in different pregnancy periods and adverse birth outcomes (co-exposure models)

Per IQR increase in		Mean difference		Odd ratios			
		Birth weight, grams (95% CI) (N=941)	Birth length, cm (95% CI) (N=927)	PTB (case: 33) (95% CI) (N=945)	LBW (case: 30) (95% CI) (N=945)	LGA (case: 108) (95% CI) (N=945)	SGA (case: 84) (95% CI) (N=945)
Estimated exposure to $PM_{2.5}$	90 days prior to conception	75.00 (-9.86, 159.86)	0.23 (-0.14, 0.60)	0.98 (0.56, 1.89)	0.41 (0.14, 1.22)	1.14 (0.55, 2.40)	1.18 (0.48, 2.92)
	First trimester	19.59 (-71.23, 110.41)	0.00 (-0.40, 0.39)	0.97 (0.26, 3.65)	0.66 (0.14, 3.05)	0.67 (0.32, 1.42)	0.73 (0.28, 1.93)
	Second trimester	-25.62 (-104.32, 53.09)	0.08 (-0.26, 0.42)	1.34 (0.37, 4.86)	0.94 (0.21, 4.21)	0.83 (0.42, 1.62)	0.69 (0.34, 1.40)
	Third trimester	13.77 (-72.33, 99.86)	-0.2 (-0.58, 0.17)	1.12 (0.31, 4.07)	0.94 (0.26, 3.35)	1.00 (0.48, 2.12)	0.57 (0.26, 1.23)
	Total pregnancy	21.13 (-36.41, 78.67)	0.02 (-0.23, 0.27)	0.73 (0.33, 1.61)	0.52 (0.24, 1.15)	0.98 (0.60, 1.61)	**0.55 (0.32, 0.96)**
Estimated exposure to NO_2	90 days prior to conception	-18.63 (-64.02, 26.76)	-0.09 (-0.29, 0.10)	1.24 (0.61, 2.49)	1.3 (0.64, 2.64)	1.27 (0.84, 1.90)	1.39 (0.90, 2.15)
	First trimester	-14.53 (-61.15, 32.09)	0.05 (-0.16, 0.25)	0.91 (0.45, 1.83)	1.14 (0.55, 2.37)	1.33 (0.89, 2.00)	**1.70 (1.07, 2.69)**
	Second trimester	-14.46 (-57.45, 28.54)	-0.08 (-0.26, 0.11)	1.22 (0.63, 2.36)	1.36 (0.68, 2.71)	1.27 (0.87, 1.87)	1.50 (1.00, 2.24)
	Third trimester	-13.13 (-64.87, 38.62)	0.04 (-0.18, 0.27)	0.77 (0.35, 1.67)	0.97 (0.43, 2.16)	1.41 (0.90, 2.23)	**1.77 (1.08, 2.91)**
	Total pregnancy	-15.02 (-49.33, 19.30)	0.00 (-0.15, 0.15)	1.08 (0.64, 1.80)	1.28 (0.75, 2.18)	1.21 (0.90, 1.63)	**1.60 (1.15, 2.23)**

All significant findings in the table are bold.

Models adjusted for maternal age at enrolment, infant’s sex, maternal BMI at 11–14 weeks' gestation, primiparity, monthly household income level, and season of births, and adjusted for the other air pollutant
