# Supplementary material for: Associations of air pollution exposures in preconception and pregnancy with birth outcomes and infant neurocognitive development: analysis of the Complex Lipids in Mothers and Babies (CLIMB) prospective cohort in Chongqing, China
Source: BMJ Open. 2024 Jul 2;14(7):e082475. doi: 10.1136/bmjopen-2023-082475 (PMC11227797; doi:10.1136/bmjopen-2023-082475)
Supplement: Author's manuscript [file bmjopen-2023-082475.draft_revisions.pdf]

BMJ Open is committed to open peer review. As part of this commitment we make the peer review history of every article we publish publicly available.

When an article is published we post the peer reviewers' comments and the authors' responses online. We also post the versions of the paper that were used during peer review. These are the versions that the peer review comments apply to.

The versions of the paper that follow are the versions that were submitted during the peer review process. They are not the versions of record or the final published versions. They should not be cited or distributed as the published version of this manuscript.

BMJ Open is an open access journal and the full, final, typeset and author-corrected version of record of the manuscript is available on our site with no access controls, subscription charges or pay-per-view fees (http://bmjopen.bmj.com).

If you have any questions on BMJ Open's open peer review process please email info.bmjopen@bmj.com

Associations of early pregnancy air pollution with adverse birth outcomes and infant neurocognitive development in the Complex Lipids in Mothers and Babies (CLIMB) cohort

Journal:	*BMJ Open*
Manuscript ID	bmjopen-2023-082475
Article Type:	Original research
Date Submitted by the Author:	28-Nov-2023

Complete List of Authors:	Chen, Yingxin; University of Leicester, Centre for Environmental Health and Sustainability<br>Kuang, Tao; Zunyi Medical and Pharmaceutical College, Department of public health and management<br>Harper, Alex; University of Leicester<br>Gulliver, John; St George's University of London<br>Zhang, Ting; Stomatological Hospital of Chongqing Medical University<br>Cai, Samuel; University of Leicester, Department of Health Sciences<br>Colombo, John ; University of Kansas<br>Han, Ting-Li; University of Auckland Liggins Institute; Canada - China - New Zealand Joint Laboratory of Maternal and Fetal Medicine<br>xia, yinyin; Chongqing Medical University<br>Hansell, Anna ; University of Leicester, Centre for Environmental Health and Sustainability<br>Zhang, Hua; The First Affiliated Hospital of Chongqing Medical University, Department of Obstetrics and Gynaecology<br>Baker, Philip ; University of Leicester, College of Medicine

Keywords:	China, Developmental neurology & neurodisability < PAEDIATRICS, Child & adolescent psychiatry < PSYCHIATRY, EPIDEMIOLOGIC STUDIES, EPIDEMIOLOGY, Maternal medicine < OBSTETRICS

SCHOLARONE™
Manuscripts

I, the Submitting Author has the right to grant and does grant on behalf of all authors of the Work (as defined in the below author licence), an exclusive licence and/or a non-exclusive licence for contributions from authors who are: i) UK Crown employees; ii) where BMJ has agreed a CC-BY licence shall apply, and/or iii) in accordance with the terms applicable for US Federal Government officers or employees acting as part of their official duties; on a worldwide, perpetual, irrevocable, royalty-free basis to BMJ Publishing Group Ltd ("BMJ") its licensees and where the relevant Journal is co-owned by BMJ to the co-owners of the Journal, to publish the Work in this journal and any other BMJ products and to exploit all rights, as set out in our licence.

The Submitting Author accepts and understands that any supply made under these terms is made by BMJ to the Submitting Author unless you are acting as an employee on behalf of your employer or a postgraduate student of an affiliated institution which is paying any applicable article publishing charge ("APC") for Open Access articles. Where the Submitting Author wishes to make the Work available on an Open Access basis (and intends to pay the relevant APC), the terms of reuse of such Open Access shall be governed by a Creative Commons licence – details of these licences and which Creative Commons licence will apply to this Work are set out in our licence referred to above.

Other than as permitted in any relevant BMJ Author's Self Archiving Policies, I confirm this Work has not been accepted for publication elsewhere, is not being considered for publication elsewhere and does not duplicate material already published. I confirm all authors consent to publication of this Work and authorise the granting of this licence.

For peer review only

Associations of early pregnancy air pollution with adverse birth outcomes and infant neurocognitive development in the Complex Lipids in Mothers and Babies (CLIMB) cohort

Yingxin Chen*[1,2], Tao Kuang*[3], Ting Zhang[4], Yutong Samuel Cai[1,2], John Colombo[5], Alex Harper[6], Ting-Li Han[7], Yinyin Xia[8], John Gulliver[9], Anna L Hansell[1,2,10], Hua Zhang[7], Philip N Baker[6,7]

1: Centre for Environmental Health and Sustainability, University of Leicester, Leicester, UK

2: The National Institute of Health Research (NIHR) Health Protection Research Unit (HPRU) in Environmental Exposure and Health at the University of Leicester, Leicester, UK

3: Department of public health and management, Zunyi Medical and Pharmaceutical College, Zunyi, 563000, Guizhou, China

4. Stomatological Hospital of Chongqing Medical University, Chongqing, 401147, China

5: Schiefelbusch Institute for Life Span Studies and Department of Psychology, University of Kansas, Lawrence, KS 66045, USA

6: College of Life Sciences, University of Leicester, Leicester, UK

7: Department of Obstetrics and Gynaecology, the First Affiliated Hospital of Chongqing Medical University, Chongqing, 400016, China

8: School of Public Health, Chongqing Medical University, Chongqing, 400016, China

9: Environmental and Exposure Sciences, Population Health Research Institute, St George's, University of London, London, UK

10: NIHR Leicester Biomedical Research Centre, Leicester General Hospital, Leicester, UK

Correspondence to: Yinyin Xia. School of Public Health, Chongqing Medical University, Chongqing, 400016, China. Email: 100118@cqmu.edu.cn

*: Contributed equally

For peer review only

For peer review only - http://bmjopen.bmj.com/site/about/guidelines.xhtml

Abstract

Objectives: to investigate the associations of traffic-related air pollution exposures in early pregnancy with birth outcomes and infant neurocognitive development

Design: cohort study

Setting: in the maternity clinics of two centres (the First Affiliated Hospital of Chongqing Medical University (FCQMU) and Chongqing Health Centre for Women and Children (CHC)).

Participants: women who were between 20 and 40 years of age and were at 11–14 weeks gestation with a singleton pregnancy were eligible for participation. Women were excluded if they had a history of premature delivery before 32 weeks of gestation, maternal milk allergy or aversion, or severe lactose intolerance. 1,273 pregnant women enrolled in 2015-2016 and 1,174 live births were included in this analysis.

Interventions: Air pollution concentrations at their home addresses, including particulate matter (PM) with diameter ≤2.5μm ($PM_{2.5}$) and nitrogen dioxide (NO_2), during pre-conception and each trimester period (TI, T2, T3) were estimated using land-use regression (LUR) models.

Primary and secondary outcome measures: birth outcomes (i.e., birthweight, birth length, preterm birth (PTB), low birth weight (LBW), large for gestational age (LGA) and small for gestational age (SGA) status) and neurodevelopment outcomes measured by the Chinese version of Bayley Scales of Infant Development (CBSID)).

Results: An association between SGA and per Interquartile range (IQR) increases in NO_2 was found during the whole pregnancy (Odd ratio (OR): 1.60, 99% confidence interval (CI): 1.03, 2.48) after co-adjusted for $PM_{2.5}$. Both $PM_{2.5}$ and NO_2 exposure in the 90 days prior to conception (but not during) were associated with lower PDI score (β: -6.15, 99% CI: -9.69, -2.61; β: -2.83, 99% CI: -4.27, -0.93, respectively).

Conclusions: NO_2 levels during pregnancy were associated with increased risk of SGA, while both $PM_{2.5}$ and NO_2 pre-conception were associated with adverse neurodevelopment outcomes at 12 months of age.

Keywords: Air pollution; birth outcomes; child cognition

For peer review only - http://bmjopen.bmj.com/site/about/guidelines.xhtml

Article summary

Strength and limitation

- We developed an LUR model to capture spatial and temporal variations of air pollution at individual level to reduce exposure misclassification if using monitoring stations.
- This is one of the few studies to investigate both pre-conception and prenatal $PM_{2.5}$ and NO_2 exposure with neurodevelopment outcomes among young infants, in the context of a relatively high air pollution urban environment.
- Our sample size was relatively small, limiting the statistical power to assess several outcomes.
- We defined exposure windows for clinically-defined trimesters.
- The performance of the NO_2 spatiotemporal model was moderate (COR-R^2: 0.39), which may introduce exposure misclassification and therefore bias in the coefficients.

For peer review only

For peer review only - http://bmjopen.bmj.com/site/about/guidelines.xhtml

Introduction

Air pollution is a major environmental factor that has been linked to a range of adverse health outcomes in children. Maternal exposure to air pollutants during pregnancy, especially $PM_{2.5}$ and NO_2, has been found to be associated with adverse birth outcomes, including PTB(1), term low birth weight (TLBW)(2), and small for gestational age (SGA) status (3). According to the developmental origins of health and disease (DOHaD) hypothesis, prenatal environmental exposures to air pollution may lead to adverse birth outcomes and subsequently increase the susceptibility to the development of certain diseases later in life (4). A number of epidemiological studies have linked prenatal air pollutant exposure to neurodevelopmental disorders such as autism spectrum disorder (ASD), attention deficit hyperactivity disorder (ADHD), and cognitive impairment (5). Although the underlying biological mechanisms are still unclear, some studies indicated that prenatal air pollution exposure may induce systemic oxidative stress that triggers intrauterine inflammation, leading to damage to several fetal organs, including the brain (6, 7).

It is also unclear that whether the adverse effects of air pollution start earlier before conception. Three months before conception was considered as a critical developmental window for gametogenesis. Air pollution exposure during three months preconception or early stages of pregnancy may have adverse effects on gametogenesis of sperm (8, 9) and ova cells (10). Exposures to $PM_{2.5}$ in preconception period have been associated with various neurodevelopmental outcomes, such as neural tube defects (11), lower psychomotor development scores (12), higher risk of ASD (13, 14), and higher risk of intellectual disability (15). While there is growing evidence for the effects of preconception $PM_{2.5}$ exposure on the risk of adverse neurodevelopmental outcomes, no study examined the effects of preconception NO_2 exposure. There are also inconsistencies across studies and heterogeneities in health outcomes and air pollutant levels (12).

Many studies have reported the effects of prenatal exposure to air pollution on neurodevelopmental function in children. However, the reported associations vary, due to the heterogeneous assessments of air pollutants exposure and neurodevelopmental outcomes (5, 16). Moreover, most of these studies were conducted in relatively developed countries where pollution is lower; little evidence has come from populations in developing countries such as China (17-20), that are more likely to experience more severe air pollution exposure.

For peer review only - http://bmjopen.bmj.com/site/about/guidelines.xhtml

The current study leveraged the Complex Lipids in Mothers and Babies (CLIMB) cohort, a prospective birth cohort recruited in Chongqing, China(21), with trimester-specific maternal $PM_{2.5}$ and NO_2 air pollution exposure derived from a spatio-temporal LUR model (22). The aim of this analysis was to examine the association between $PM_{2.5}$ and NO_2 air pollution pre- and during pregnancy, with birth and infant neurocognitive development outcomes at 12 months of age.

A key aspect in all studies like this one is the accuracy of documenting exposure; a recent Chinese study determined air pollution exposure based on the nearest monitoring station data (18) may not reflect the temporal and spatial variability of pollutant exposures among participants. The current study employed common air pollutant exposure models based on advanced geographic information systems (GIS), to address some of the limitations of previous studies (23).

In addition, the timing of in exposure is also critical in determining the effects of exposure on developmental outcomes. Indeed, the evidence from previous studies on the sensitive time window for exposure pre- and during pregnancy remains inconclusive. Some studies have indicated that the early-to-mid pregnancy phase may be a critical period in terms of the impact of air pollution on neurodevelopment(17, 24). Early pregnancy is particularly important for neurogenesis and neuromigration, making it a susceptible period (25). However, actual findings have been varied, with some studies showing stronger associations for middle or late pregnancy (18, 19, 26). More studies identifying critical periods are needed to enhance our understanding of how pre-conception and prenatal air pollution exposure affect neurodevelopment. With this cohort, we are able to examine the effects of exposure pre-conception, at each trimester, as well as across the entire pregnancy.

Methods

Study population

A description of participant recruitment in the CLIMB cohort has been described previously (27). In brief, women who were between 20 and 40 years of age and were at 11–14 weeks gestation with a singleton pregnancy were eligible for participation. Women were excluded if they had a history of premature delivery before 32 weeks of gestation, maternal milk allergy or aversion, or severe lactose intolerance.

For peer review only - http://bmjopen.bmj.com/site/about/guidelines.xhtml

From September 2015 to November 2016, a total of 1,500 women were recruited into the cohort. Participants attended six visits at the First Affiliated Hospital of Chongqing Medical University and Chongqing Health Centre for Women and Children: 11–14 weeks' gestation (visit 1), 22–28 week's gestation (visit 2), 32–34 week's gestation (visit 3), at birth (visit 4), 6 weeks postnatal (visit 5), and 12 months postnatal (visit 6).

Women who withdrew from the study (n = 146), terminated their pregnancy (n = 29), miscarried (n = 12) or were lost to follow-up (n = 40) were excluded from the analysis, leaving a sample size of 1,273 women. Analyses were restricted to mothers whose detailed residential addresses during pregnancy were known (**Figure 1**). A total of 1,174 live births were thus included in the pregnancy and neonatal outcomes analysis. Subsequently, at 1 year follow-up, 946 children were included in the analysis of neurodevelopment outcomes.

Exposure assessment

The address of participants was collected at the first visit. Exposure assessment based on spatiotemporal land use regression (LUR) models for $PM_{2.5}$ and NO_2 were developed for the study region. The study area focused on the urban center of the Chinese municipality of Chongqing (**Figure 2**). A description of the methodology of exposure modelling has been reported previously (22). Briefly, the models included both spatial and temporal components of exposure. $PM_{2.5}$ and NO_2 concentration data were collected from 17 routine monitoring sites operated by the Chongqing Environmental Monitoring Center in 2015-2016. For the spatial component of models, we calculated annual average concentrations of each pollutant in 2015, and fit linear regression models using five groups of geographic data (road network, land use, topography, vegetation, and population density) as spatial predictor variables. For the temporal component of models, we calculated the residuals from the spatial component at each monitoring site on a daily basis by subtracting the predicted annual average concentration from the observed daily average concentrations measured in 2015 and 2016, and then fitted generalised additive models (GAM) using seven groups of meteorological data (temperature, amount of rainfall, rainfall events, relative humidity, horizontal visibility, wind direction and wind speed) as temporal predictor variables. The meteorological variables were used to account for the influence of weather on the change in air pollution concentration over time. To account for the remaining spatial autocorrelation, the smoothed terms of longitude and latitude were fit to spatiotemporal residuals which were calculated by subtracting the sum of the spatial

For peer review only - http://bmjopen.bmj.com/site/about/guidelines.xhtml

temporal predictions from the measured daily average concentrations in 2015 and 2016. The performance of the $PM_{2.5}$ spatiotemporal models was good (COR-R^2: 0.72) and the NO_2 spatiotemporal model was moderate (COR-R^2: 0.39) when providing concentration estimates in absolute terms.

Combining the family address coordinates of each pregnant woman and the gestation period of the pregnancy (calculated from the date of last menstrual period to the date of delivery), we used this spatiotemporal model to estimate the average exposure of each pregnant woman in 90 days prior to pregnancy (90D), first trimester (T1), second trimester (T2), third trimester (T3) and whole pregnancy period (WP), respectively.

Outcomes

Birth Outcomes

Birth outcomes were determined by experienced obstetricians and abstracted from the medical records. Birth outcomes included: birthweight (in grams), birth length (in centimetres), PTB, low birth weight (LBW), large for gestational age (LGA) and SGA status (28). PTB was defined as delivery before 37 weeks. LBW was defined as weighing less than 2500 g at birth. LGA and SGA were indicated by birth weight greater than and less than the 90th and 10th percentile within this study for the gestational age by sex respectively (29). Term low birth weight was not considered due to a small sample size of only 8 cases.

Neurodevelopment outcomes

The Chinese version of Bayley Scales of Infant Development (CBSID) was used to assess mental and psychomotor development for infants in this study. The CBSID is appropriate for evaluation of infants from 2–30 months old (30) and takes into consideration each infant's age in days. Infants were assessed at around 12 months (range from 11 months and 15 days to 12 months and 15 days) by a trained examiner, with ages corrected for preterm birth. These scales have been formally adapted to the Chinese language and locally standardized to become culturally appropriate, with two main indexes: the Mental Development Index (MDI) and the Psychomotor Development Index (PDI). The MDI component comprised 163 items and assessed age-appropriate items related to cognitive functioning, personal and social

For peer review only - http://bmjopen.bmj.com/site/about/guidelines.xhtml

development, and language development (see **eTable 1** in the Supplement). The PDI component comprised 81 items and assessed age-appropriate fine and gross motor skills (see **eTable 2** in the Supplement). The test provided raw scores for mental and psychomotor development that were converted to standardized (in terms of age in days) MDI and PDI scores, based on norms for the Chinese population. As with other forms of the Bayley test these index scores have a mean of 100, and a standard deviation of 15, with a lower score reflecting poorer performance (31). If an infant refused to cooperate with the examiners to finish the task, a second assessment was arranged within two weeks. If the infant could not cooperate at the second BSID assessment, their data were classified as missing.

Covariates

Socio-demographic data were collected through interviews by trained nurses. The following potential confounders were identified: maternal age at enrolment (in years), infant sex (male/female), maternal BMI at 11–14 weeks' gestation (kg/m^2), parity (Yes/No), monthly household income level (categorized as: <2,000 yuan, 2,000–7,000 yuan, 7,000–10,000 yuan, or >10,000 yuan), season of birth (categorized as: Spring (Mar-May), Summer (Jun-Aug), Autumn (Sep-Nov) or Winter (Dec-Feb)). Marital status (single/married) and smoking or drinking during pregnancy (Yes/no) were not taken into account in this analysis because of the homogeneity of the study population (i.e., 98.6% women were married and 99.6% women reported not smoking or drinking alcohol during pregnancy).

Statistical analyses

Data were described in terms of mean ± SD or median (IQR) for continuous variables, or as percentages for categorical variables. Modelled $PM_{2.5}$ and NO_2 exposure levels in 90D, T1, T2, T3 and WP were considered separately. We examined the correlation between each of the exposures in the different pregnancy periods. For birth outcomes, multivariable linear regression was used for continuous outcomes (e.g., birth weight and birth length) to estimate β coefficient and their 95% confidence intervals (CIs) and multivariable logistic regression for binary outcomes (e.g., PTB, LBW, LGA and SGA status) to estimate odds ratio (OR) and 99% CIs. For mental and psychomotor development (e.g., MDI and PDI scores), multivariate linear), multivariable linear regression models were fit to estimate β coefficient and their 99%CIs.

For peer review only - http://bmjopen.bmj.com/site/about/guidelines.xhtml

For peer review only

Models were adjusted for maternal age at enrolment, infant sex, maternal BMI at 11–14 weeks gestation, primiparity, monthly household income level, and season of birth. We also ran co-exposure models to estimate associations of one air pollutant with outcomes adjusted for the other air pollutant (i.e., $PM_{2.5}$ in T1 adjusted for NO_2 in T1). Effect estimates are reported for each IQR increase of $PM_{2.5}$ and NO_2. We also tested for indirect effects of NO_2 on PDI mediated by SGA with "'medsem" commands. All analyses were performed using STATA version 17. A p-value of <0.01 was considered statistically significant to address multiple comparisons in the analyses.

For peer review only

For peer review only - http://bmjopen.bmj.com/site/about/guidelines.xhtml

Results

Study Participants

Participant characteristics are presented in **Table 1**. Of those participating women, the mean age was 28 years and mean BMI was 21.5 kg/m^2. 98.0% of women were of Han ethnicity, 77.9% were primiparous, and 67.6% had completed tertiary education. 33 (2.8%), 30 (2.6%), 108 (9.2%), 84 (7.2%) of the 1,174 births considered in this analysis were classified as PTB, LBW, LGA and SGA, respectively. For those 946 children who completed the BSID test, the mean MDI score was 94.7 (SD: 17.7) and the mean MDI score was 87.4 (SD: 14.9).

Exposure assessment

Median $PM_{2.5}$ exposure levels were 57.31 μg/m3 (IQR: 5.76) and median NO^2 exposure levels were 50.46 μg/m3 (IQR: 5.51) during the whole pregnancy period (**eTable 3 in the Supplement**). For $PM_{2.5}$, the concentration in the pre-conception and T1 were considerably lower than other periods, close to 10 μg/m3. The between-trimester and 90D values for NO_2 were generally moderately correlated (Pearson's r > 0.5). The correlation coefficients of $PM_{2.5}$ were more variable between time periods reflecting the high variability of $PM_{2.5}$ concentrations, with values ranging from -0.78 to +0.68. Correlations between $PM_{2.5}$ and NO_2 in the same pregnancy period were moderately correlated (Pearson's r ~0.6, **eTable 4 in the Supplement**).

Association with birth outcomes

In the unadjusted models (**eTable 5 in the Supplement**), higher exposure concentrations of $PM_{2.5}$ in T3 were significantly associated with lower birth length (β: -0.32, 99% CI: -0.57, -0.07; per IQR increase). A risk between SGA and increases in NO_2 (per IQR) was found in T2 (OR: 1.46, 99% CI: 1.01, 2.11), T3 (OR: 1.58, 99% CI: 1.03, 2.42) and in the whole pregnancy period (OR: 1.44, 99% CI: 1.04, 2.00). We observed no evidence of associations of NO_2 with overall birth weight, birth length and other adverse birth outcomes (e.g., PTB, LBW, and LGA).

In the adjusted models (**Table 2**), we found slightly reduced effect size for NO_2 and SGA in the whole pregnancy period (OR: 1.33, 99% CI:0.92, 1.75) compared with the unadjusted model. We observed no evidence of associations with birth length in the adjusted models. After

For peer review only - http://bmjopen.bmj.com/site/about/guidelines.xhtml

co-adjustment for $PM_{2.5}$ (**see eTable 6 in the Supplement**), the association of NO_2 with SGA became significant in the whole pregnancy period (OR: 1.60, 99% CI: 1.03, 2.48)

Association with infant neurodevelopment outcomes

In unadjusted models, $PM_{2.5}$ exposure in the 90 days prior to conception was associated with lower MDI and PDI scores in offspring (β: -3.54, 99% CI: -5.94, -1.13; β: -3.42, 95% CI: -5.44, -1.40) (**eTable 7 in the Supplement**). We also observed an unexpected positive association between $PM_{2.5}$ exposures in second trimester with MDI (β: 4.21, 99% CI: 1.87, 6.56) and PDI (β:2.63, 99%CI: 0.65, 4.61). Exposure to NO_2 was associated with lower PDIs in the 90 days prior to conception (-2.86, 99% CI: -4.46, -1.26), and T3 (-1.97, 99% CI: -3.70, -0.23). We did not observe any association between NO_2 and MDI in any pregnancy periods.

In the adjusted models (Table 5), we found $PM_{2.5}$ exposure in the 90 days prior to conception was associated with lower PDI scores (β: -6.15, 99% CI: -9.69, -2.61). Similarly, there was also a significant association of increased NO_2 exposure and lower PDI score (β: -2.83, 99% CI: -4.27, -0.93). However, the positive association between $PM_{2.5}$ exposures in second trimester with PDI (β: 3.76, 99% CI: 0.49, 7.02) remained. We did not observe any association with MDI in any pregnancy periods.

In the co-exposure models (**see eTable 8 in the Supplement**), $PM_{2.5}$ exposure in the 90 days prior to conception was associated with lower PDI scores (β: -4.74, 99% CI: -8.67, -0.81). We also observed a positive association between $PM_{2.5}$ exposures in second trimester with PDI (β: 5.51, 99% CI: 1.86, 9.16). Exposure to NO_2 was significantly associated with lower PDI in the second trimester (β: -2.11, 99% CI: -4.11, -0.12) and whole pregnancy period (β: -1.68, 99% CI: -3.28, -0.08). In the mediation analyses, we did not observe any indirect effect for $PM_{2.5}$ and NO_2 on BSID scores mediated by SGA in all pregnancy periods (**see eTable 9 and 10 in the Supplement**).

Discussion

We analyzed associations between modelled $PM_{2.5}$ and NO_2 pre- and during pregnancy with birth and neurodevelopment outcomes in singleton children born in a south-western metropolis of China in 2015-16. We found the likelihood of SGA increased by 60% per IQR higher

For peer review only - http://bmjopen.bmj.com/site/about/guidelines.xhtml

exposure to NO_2 in the whole pregnancy periods after adjusted for maternal age at enrolment, infant's sex, maternal BMI at 11–14 weeks' gestation, primiparity, monthly household income level, and season of births and $PM_{2.5}$. For childhood cognitive development, increased exposure to $PM_{2.5}$ and NO_2 in the 90 days prior to conception were both associated with lower PDI scores, with the effect size per IQR being higher for $PM_{2.5}$ than for NO_2. We also found a positive association between $PM_{2.5}$ exposures in second trimester with PDI. While SGA was associated with NO_2 exposures, SGA was not found to mediate the effects of $PM_{2.5}$ and NO_2 on BSID scores in this study.

Many studies from other geographic areas, including Europe (32-34), the United States (24, 26), and Asia (17, 35-37) have found that prenatal air pollution exposure has a negative impact on a variety of neurodevelopmental outcomes. Our finding of a negative association between prenatal NO_2 air pollution exposure and infant neurocognitive development is consistent with these reports. A recent Chinese birth cohort study of 15,778 child-mother pairs in Foshan reported that maternal NO_2 exposure during pregnancy was associated with increased risk of suspected developmental delay (OR: 1.06, 95% CI: 0.94, 1.19) measured by a five-domain scale and developmental quotient (DQ) (17). A birth cohort study of 520 mother-child pairs in South Korea reported that maternal NO_2 exposure during pregnancy was associated with impairment of psychomotor development ($\beta = -1.30$, $p = 0.05$) but – as in the present study - not with cognitive function ($\beta = -0.84$, $p = 0.20$) (35). However, results from previous research varied by air pollutants. For example, a Chinese study of 1193 mother-newborn pairs in Changsha found significant associations between $PM_{2.5}$ exposure in trimester 2 and lower neurobehavioral developmental scores, while other air pollutants such as PM_{10}, carbon monoxide (CO), and Sulfur dioxide (SO_2) had null or even reverse associations. In this study, we observed that the negative effect of NO_2 exposure during pregnancy on PDI is significant at 5% level, whereas no such effect was found for $PM_{2.5}$; in the co-exposure model, this negative effect of NO_2 was stronger and became significant at 1% level after adjustment for $PM_{2.5}$. This heterogeneity may relate to the time of exposure assessment, the type of instruments or evaluators, and the levels of air pollution. In addition, air pollution mixtures may have differed among the study regions, thus there are several potential explanations for the heterogeneity of the findings.

To date, most studies on prenatal air pollution exposure and child neurodevelopment have been conducted in developed countries with relatively low levels of air pollution. In this study, the level of air pollution was higher (median $PM_{2.5}$: 57.31 μg/m^3, IQR: 5.76; median NO_2:

For peer review only - http://bmjopen.bmj.com/site/about/guidelines.xhtml

50.46 μg/m^3, IQR: 5.51) compared to studies in developed countries such as Europe and the United States. In a multi-centre European cohort, the mean $PM_{2.5}$ and NO_2 exposure levels during pregnancy were 13.4 μg/m^3 and 11.5 μg/m^3 (32). Researchers found that the psychomotor development score significantly decreased by 0.68 points (95% CI: -1.25, -0.11) for every 10 μg/m^3 increase in NO_2, and there was also a non-significant decrease of 1.64 points (95% CI: -3.47, 0.18) for every 5 μg/m^3 increase in $PM_{2.5}$ during pregnancy (32). Noticeably, we did not find associations of either NO_2 or $PM_{2.5}$ during pregnancy with neurodevelopmental delay. Factors such as the types of pollutants and concentrations may differ between China and other regions with a lower air pollution level, leading to variations in the observed effects.

Contrary to expectations, we found significant positive associations between prenatal exposure to $PM_{2.5}$ air pollution in the second trimester and PDI. Given the prior literature and the high variability observed here, we believe that this is likely spurious/sample specific. Several epidemiological studies have reported associations between prenatal exposure to high levels of $PM_{2.5}$ and lower neurodevelopment in children ranging in age from 6 months to 6 years (12, 33, 38-40). In agreement with our findings, a multi-centre cohort study from six European countries investigated the effects of prenatal exposure to multiple air pollutants including $PM_{2.5}$, PM_{10}, coarse particles, NO_2 and nitrogen oxides (NOx) among 9482 children between 1 and 6 years; the authors found nonsignificant positive associations between prenatal $PM_{2.5}$ exposure and normal neurodevelopment (β: 1.64, 95% CI: −3.47, 0.18; per 5 μg/m^3 increase in $PM_{2.5}$) (32). Similarly, another study examining the effects of multiple pollutant exposures on early childhood cognition at 40 days of age in a highly exposed area of Spain also found PM_{10}, PM_{coarse}, $PM_{2.5absorbance}$, NO_2, NO_x, and Ozone (O_3) were linked to lower motor function in children, except for $PM_{2.5}$ (41). The inconsistent findings could be because of heterogeneity between studies in terms of exposure (e.g., exposure assessment methods used, $PM_{2.5}$ exposure levels, or composition of $PM_{2.5}$).

Aside from the conflicting findings regarding prenatal $PM_{2.5}$ exposure and neurodevelopmental outcomes, results regarding the most potential sensitive time windows before and during pregnancy are also inconclusive. Some studies suggested that early-to-mid pregnancy might be a potential sensitive period (17, 24), while other studies found stronger associations for mid-ate pregnancy, thus results are equivocal (18, 19, 26). The potential biological mechanisms by which air pollution could affect neurodevelopment are not yet clearly understood. There is evidence suggesting that exposure to prenatal $PM_{2.5}$ could potentially induce maternal immune activation during pregnancy (42). Higher levels of cytokines or reactive oxygen species may

For peer review only - http://bmjopen.bmj.com/site/about/guidelines.xhtml

potentially interfere fetal neurodevelopment through three mechanisms: crossing the placental barrier into the fetal body, inducing fetal immune dysregulation, and contributing to inadequate placental perfusion that affects nutritional processes and oxygenation of maternal blood(43). More research is needed to investigate trimester effects of air pollution on neurodevelopment and provide better understanding on the underlying biological mechanisms. Our study is the first to consider an exposure window 90 days prior to conception for NO_2. A novel observation is that effects of NO_2 or $PM_{2.5}$ air pollution on child cognition can be seen at least 90 days prior to conception, representing a potentially vulnerable periods in relation to air pollution on neurodevelopment. Similar results were found in previous study recruited 1329 mother-child pairs in Wuhan, China (12). This study reported a higher level of $PM_{2.5}$ during preconception (Median: 76.1 μg/m^3) and in the first trimester (Median: 82.3 μg/m^3). This study found for each doubling of $PM_{2.5}$ exposure during preconception, children's PDI scores was reduced by 8.23 (95% CI: -10.01, -6.44) points. A potential explanation is that preconception air pollution exposures induce genetic and epigenetic alterations in sperm, that increase the risk of adverse health outcomes in offspring(44, 45). To date, all studies examined the effect of maternal preconception exposure while omitting paternal exposures (16). Future studies should consider the effect of preconception paternal exposure in relation to childhood health outcomes.

This study has several strengths. We developed an LUR model to capture spatial and temporal variations of air pollution at individual level to reduce exposure misclassification if using monitoring stations. This is one of the few studies to investigate both pre-conception and prenatal $PM_{2.5}$ and NO_2 exposure with neurodevelopment outcomes among young infants, in the context of a relatively high air pollution urban environment.

A major limitation of this study was that our sample size was relatively small, limiting the statistical power to assess several outcomes, although the higher exposures in Chongqing than in some other studies may increase probability of detecting effects. In terms of limitations, due to a lack of information on participant time-activity patterns, exposure estimates in this study refer only to ambient concentrations at home addresses, and no other activity spaces (e.g., indoor, workplace, commuting) were considered. We may have thus underestimated total air pollution exposure. Second, we defined exposure windows for clinically-defined trimesters; sensitive periods may be shorter or longer than 3 months, or they may exist in the overlap of multiple trimesters. However, we were unable to investigate the sensitive time windows using established methods such as distributed lag non-linear models due to the lack of highly time-resolved air pollution estimates. Third, the performance of the NO_2 spatiotemporal model was

For peer review only - http://bmjopen.bmj.com/site/about/guidelines.xhtml

moderate (COR-R^2: 0.39), which may introduce exposure misclassification and therefore bias in the coefficients. Finally, we were unable to include some other air pollutants such as polycyclic aromatic hydrocarbons (PAH), black carbon (BC) and Ozone, which have bene found particular harmful to neurodevelopment in children (46).

Conclusion

This study provides evidence for an association between NO_2 exposure pre- and during pregnancy with birth and neurodevelopmental outcomes in a birth cohort in Chongqing, China. Exposure to NO_2 and $PM_{2.5}$ exposure before pregnancy was associated with a lower psychomotor development score and further studies are warranted.

For peer review only

For peer review only - http://bmjopen.bmj.com/site/about/guidelines.xhtml

399 **List of abbreviations**

ADHD	Attention deficit hyperactivity disorder
ASD	Autism spectrum disorder
BMI	Body mass index
BSID	Bayley Scales of Infant Development
CBSID	Chinese version of Bayley Scales of Infant Development
CI	Confidence interval
CLIMB	Complex Lipids in Mothers and Babies
CO	Carbon monoxide
DOHaD	Developmental origins of health and disease
DQ	Developmental quotient
GIS	Geographic information systems
IQR	Interquartile range
LBW	Low birth weight
LGA	Large for gestational age
LUR	Land-use Regression
MDI	Mental Development Index
NO_x	Nitrogen oxides
NO_2	Nitrogen dioxide
OR	Odd ratio
O_3	Ozone
PDI	Psychomotor Development Index
PM	Particulate matter
$PM_{2.5}$	Particulate matter with diameter ≤2.5μm

For peer review only - http://bmjopen.bmj.com/site/about/guidelines.xhtml

For peer review only

PTB	Preterm birth
SGA	Small for gestational age
SO_2	Sulfur dioxide
TLBW	Term low birth weight
T1	First trimester
T2	Second trimester
T3	Third trimester
WP	Whole pregnancy period
90D	90 days prior to pregnancy

For peer review only - http://bmjopen.bmj.com/site/about/guidelines.xhtml

Declarations

Ethics approval and consent to participate

Ethical approval for this study was granted by the Ethics Committee of Chongqing Medical University (#2014034). The participants provided their written informed consent to participate in this study. Written informed consent was obtained from the individual(s) for the publication of any potentially identifiable images or data included in this article.

Availability of data and materials

The data that support the findings of this study are available from Chongqing Medical University but restrictions apply to the availability of these data, which were used under license for the current study, and so are not publicly available. Data are however available from the authors upon reasonable request and with permission of Chongqing Medical University.

Conflicts of interests

The authors declare that they have no conflicts of interests.

Funding

This work was supported by the National Natural Science Foundation of China (No. 81971406, 82271715), The 111 Project (Yuwaizhuan (2016)32), Chongqing Science & Technology Bureau (CSTB2022NSCQ-MSX1680), Youth Innovation Team Development Support Program of Chongqing Medical University (W0083), and Smart Medicine Research Project of Chongqing Medical University (No. ZHYX202103), Zunyi science and technology plan project (Zunshikehe HZ (2022)153).

Author statement

Y. X., T.L.H., H.Z. and P.B. conceived and designed research; T.Z., Y.X. and H.Z. recruited the patients and collected the samples; T.K., A.H., and J.G. constructed the air pollution model; Y.C. analyzed, interpreted the data and prepared the figures; Y.C and T.K were major contributors in writing the manuscript text; YSC, JC, TLH, YX, ALH, and PNB substantively revised the manuscript; All authors read and approved the final manuscript.

Acknowledgement

For peer review only

For peer review only - http://bmjopen.bmj.com/site/about/guidelines.xhtml

429 The research was supported by National Institute for Health Research (NIHR) Health Protection Research Unit in Environmental Exposures and Health, a partnership between UK Health Security Agency, the Health and Safety Executive and the University of Leicester and by the NIHR Leicester Biomedical Research Centre (BRC). The views expressed are those of the author(s) and not necessarily those of the NIHR, the Department of Health and Social Care or UK Health Security Agency.

For peer review only

Tables and figures

Figure 1 Flow chart of the study population in CLIMB

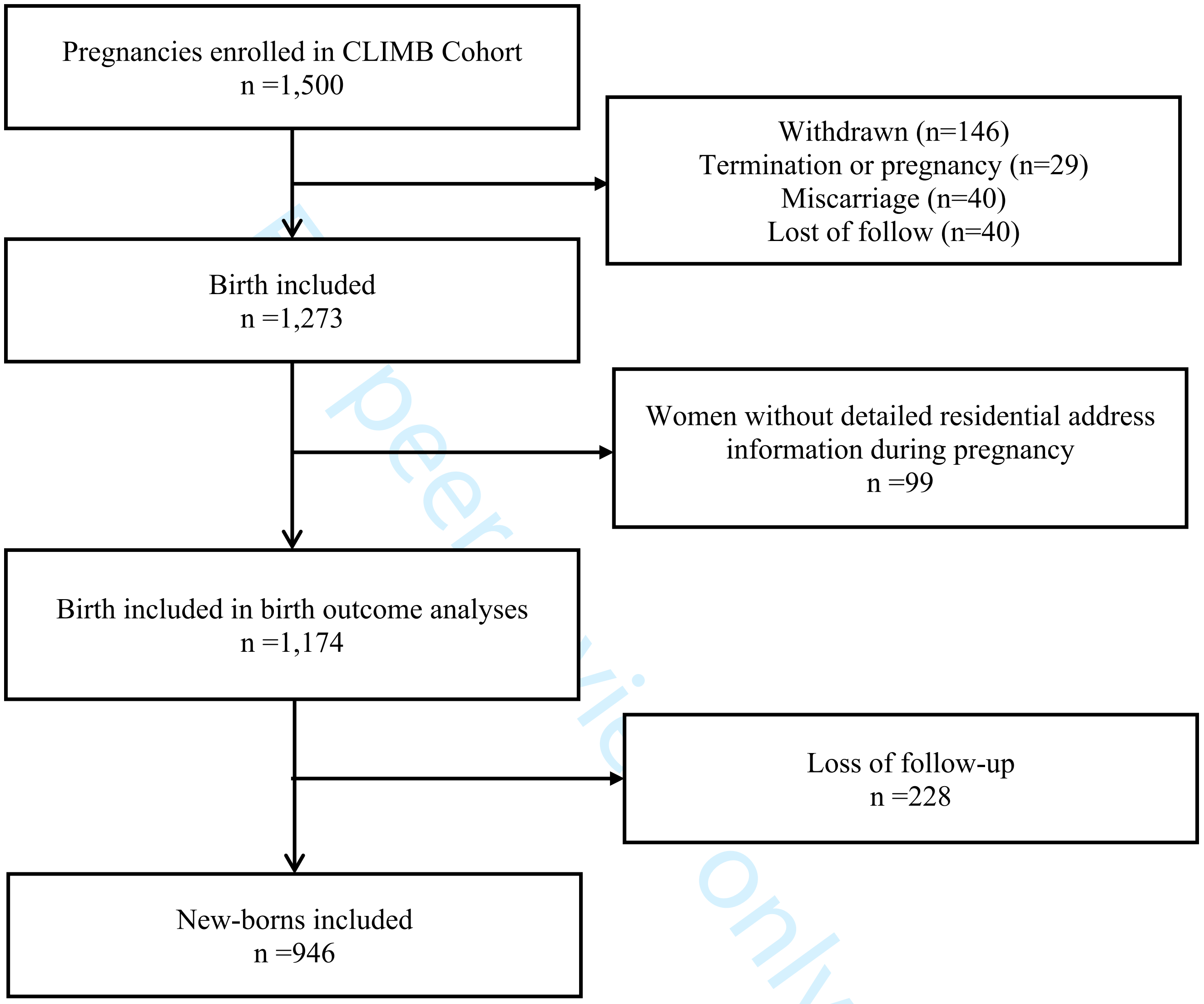

For peer review only - http://bmjopen.bmj.com/site/about/guidelines.xhtml

Figure 2 Study area and location of monitoring sites (OpenStreetMap contributors, 2015; https://data.nextgis.com/en/region/CN-50/).

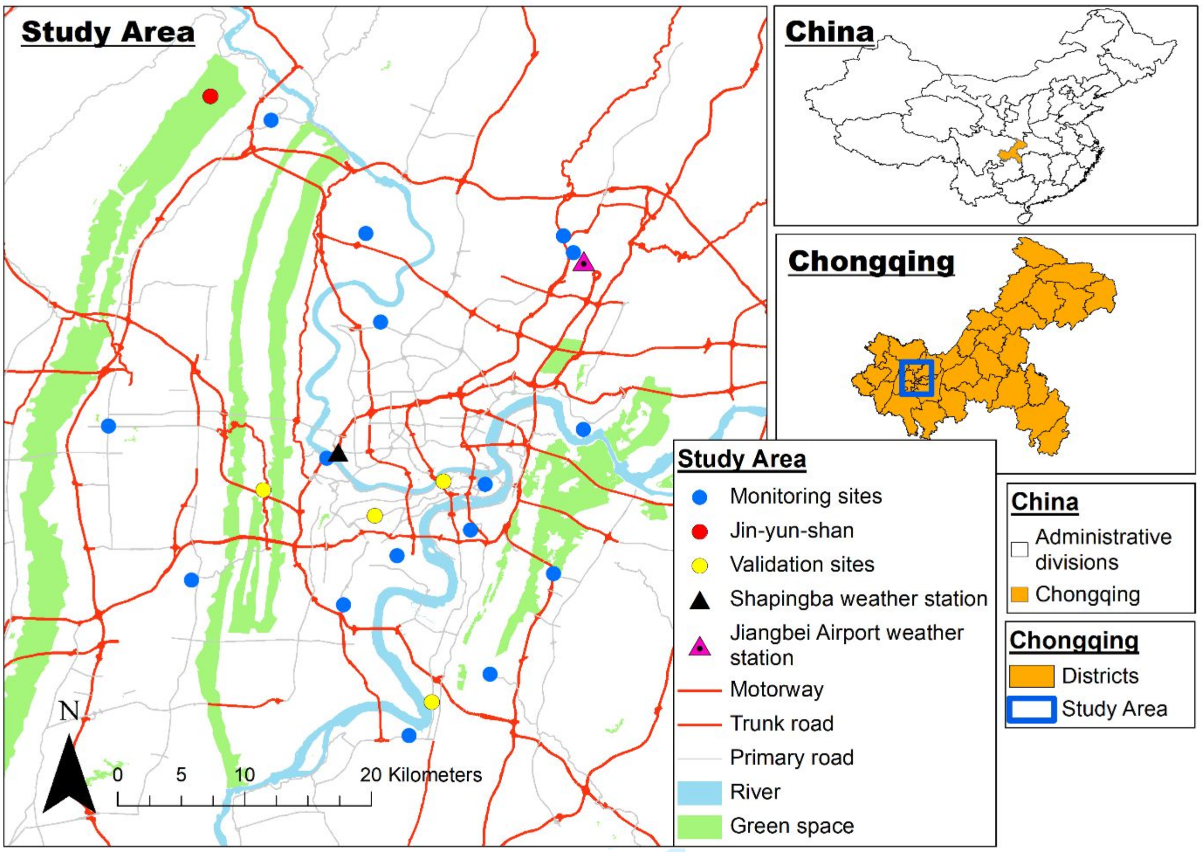

For peer review only - http://bmjopen.bmj.com/site/about/guidelines.xhtml

Table 1 Characteristics of study sample in the CLIMB cohort (N = 1,174)

Characteristic of mothers	N	n (%) or mean ± SD	Characteristic of child	N	n (%) or mean ± SD
Maternal age (Years)	1,174	28.7 ± 3.5	(week)	1,174	39.4 ± 1.5
BMI (kg/m^2)	1,174	21.5 ± 2.9	Birth weight (g)	1,165	3314.4 ± 428.8
Han ethnicity (%)	1,174		Birth length (cm)	1,149	49.7 ± 1.9
Yes		1,151 (98.0%)	Apgar score at 1 min	1,035	9.4 ± 1.3
No		23 (2.0%)	Apgar score at 5 min	1,035	9.9 ± 3.0
Marital status (%)	1,174		New born sex	1,172	
Single		16 (1.4%)	Female		561 (47.9%)
Married		1,158 (98.6%)	Male		611 (52.1%)
Primiparity (%)	1,174		Birth outcomes		
Yes		914 (77.9%)	Preterm birth (PTB)	1,174	
No		260 (22.1%)	Yes		33 (2.8%)
History of miscarriage or abortion (%)	1,174		No		1,141 (97.2%)
Yes		553 (47.1%)	Low birth weight (LBW)	1,174	
No		621 (52.9%)	Yes		30 (2.6%)
Smoking/drinking during pregnancy (%)	1,174		No		1,141 (97.2%)
Yes		5 (0.4%)	Large for gestational age (LGA)	1,174	
No		1,169 (99.6%)	Yes		108 (9.2%)
Education level	946		No		1,066 (90.8%)
Low: High school or below		306 (32.3%)	Small for gestational age (SGA)	1,174	
High: College/uni or above		640 (67.6%)	Yes		84 (7.2%)
Job	946		No		1,090 (92.8%)
Full-time		762 (80.5%)	BSID test	946	
Housewife		82 (8.7%)	MDI (mean ± SD)		94.7 ± 17.7
Others		102 (10.8%)	PDI (mean ± SD)		87.4 ± 14.9
Household income (Monthly)	946		Birth season	1,174	
<2000 RMB		186 (19.7%)	Spring (Mar-May)		411 (35.01%)
2000-4000 RMB		329 (34.8%)	Summer (Jun-Aug)		263 (22.40%)
4000-7000 RMB		292 (30.9%)	Autumn (Sep-Nov)		198 (16.87%)
7000-10000 RMB		139 (14.7%)	Winter (Dec-Feb)		302 (25.72%)

For peer review only

For peer review only - http://bmjopen.bmj.com/site/about/guidelines.xhtml

435 **Table 2 Associations between $PM_{2.5}$ and NO_2 exposure in different pregnancy periods and adverse birth outcomes (adjusted models)**

		Mean difference		Odd ratios			
		Birth weight, grams	Birth length, cm	PTB (case: 33)	LBW (case: 30)	LGA (case: 108)	SGA (case: 84)
		99% CI	99% CI	99% CI	99% CI	99% CI	99% CI
	Per IQR increase in	(N=941)	(N=927)	(N=945)	(N=945)	(N=945)	(N=945)
Estimated exposure to $PM_{2.5}$	90 days prior to conception	59.73 (-40.56, 160.01)	0.15 (-0.28, 0.58)	0.24 (0.06, 1.01)	0.49 (0.13, 1.76)	1.4 (0.59, 3.33)	1.66 (0.58, 4.73)
	First trimester	6.21 (-99.01, 111.42)	0.04 (-0.42, 0.50)	0.88 (0.19, 4.02)	0.76 (0.14, 4.24)	0.86 (0.36, 2.05)	1.33 (0.45, 3.95)
	Second trimester	-37.64 (-129.82, 54.54)	0.02 (-0.38, 0.42)	1.62 (0.37, 7.05)	1.34 (0.26, 6.92)	1 (0.46, 2.20)	0.94 (0.41, 2.15)
	Third trimester	4.2 (-97.55, 105.95)	-0.17 (-0.62, 0.27)	0.92 (0.20, 4.17)	0.92 (0.21, 4.06)	1.29 (0.53, 3.13)	0.83 (0.33, 2.06)
	Total pregnancy	8.01 (-56.57, 72.59)	0.02 (-0.27, 0.30)	0.77 (0.31, 1.91)	0.62 (0.25, 1.56)	1.15 (0.65, 2.02)	0.84 (0.45, 1.57)
Estimated exposure to NO_2	90 days prior to conception	-1.03 (-54.75, 52.68)	-0.04 (-0.27, 0.20)	0.84 (0.37, 1.91)	1.04 (0.44, 2.42)	1.31 (0.82, 2.10)	1.45 (0.88, 2.38)
	First trimester	-9.78 (-63.78, 44.22)	0.04 (-0.19, 0.28)	0.9 (0.41, 2.00)	1.03 (0.46, 2.32)	1.21 (0.76, 1.92)	1.57 (0.94, 2.62)
	Second trimester	-20.82 (-71.18, 29.54)	-0.06 (-0.28, 0.16)	1.31 (0.61, 2.81)	1.34 (0.62, 2.88)	1.21 (0.78, 1.89)	1.36 (0.85, 2.19)
	Third trimester	-9.5 (-70.66, 51.65)	-0.01 (-0.28, 0.26)	0.79 (0.32, 1.97)	0.95 (0.37, 2.43)	1.42 (0.83, 2.42)	1.51 (0.84, 2.71)
	Total pregnancy	-8.45 (-46.96, 30.06)	0 (-0.17, 0.17)	0.97 (0.54, 1.74)	1.04 (0.57, 1.89)	1.2 (0.86, 1.69)	1.33 (0.92, 1.91)

Models adjusted for maternal age at enrolment, infant's sex, maternal BMI at 11–14 weeks' gestation, primiparity, monthly household income level, and season of births.

For peer review only - http://bmjopen.bmj.com/site/about/guidelines.xhtml

437 **Table 3 Associations between $PM_{2.5}$ and NO_2 exposure in different pregnancy periods**
438 **and BSID scores (adjusted models)**

		Mean difference	
		MDI 99% CI	PDI 99% CI
	Per IQR increase in	(N=946)	(N=946)
Estimated exposure to $PM_{2.5}$	90 days prior to conception	-1.98 (-6.21, 2.25)	**-6.15 (-9.69, -2.61)**
	First trimester	-1.66 (-6.08, 2.76)	-2.11 (-5.84, 1.62)
	Second trimester	3.79 (-0.08, 7.66)	**3.76 (0.49, 7.02)**
	Third trimester	-2.73 (-7.01, 1.55)	-1.37 (-4.99, 2.26)
	Total pregnancy	-0.27 (-2.99, 2.45)	0.23 (-2.07, 2.53)
Estimated exposure to NO_2	90 days prior to conception	-0.72 (-2.98, 1.55)	**-2.83 (-4.72, -0.93)**
	First trimester	0.59 (-1.68, 2.86)	-1.91 (-3.83, 0.00)
	Second trimester	0.56 (-1.56, 2.68)	-0.75 (-2.54, 1.04)
	Third trimester	0.51 (-2.06, 3.09)	-1.92 (-4.09, 0.26)
	Total pregnancy	0.41 (-1.22, 2.03)	-1.15 (-2.52, 0.21)

Models adjusted for maternal age at enrolment, infant's sex, maternal BMI at 11–14 weeks' gestation, primiparity, monthly household income level, and season of births.

439

For peer review only

Reference

1. Llop S, Ballester F, Estarlich M, Esplugues A, Rebagliato M, Iniguez C. Preterm birth and exposure to air pollutants during pregnancy. Environmental Research. 2010;110(8):778-85.
2. Ambient air pollution and low birthweight: a European cohort study (ESCAPE). The Lancet Respiratory Medicine. 2013;1(9):695-704.
3. Stieb DM, Chen L, Hystad P, Beckerman BS, Jerrett M, Tjepkema M, et al. A national study of the association between traffic-related air pollution and adverse pregnancy outcomes in Canada, 1999–2008. Environmental Research. 2016.
4. Barker DJ. The origins of the developmental origins theory. Journal of internal medicine. 2007;261(5):412-7.
5. Volk HE, Perera F, Braun JM, Kingsley SL, Gray K, Buckley J, et al. Prenatal air pollution exposure and neurodevelopment: A review and blueprint for a harmonized approach within ECHO. Environmental research. 2021;196:110320.
6. Feng S, Dan G, Liao F, Zhou F, Wang X. The health effects of ambient PM2.5 and potential mechanisms. Ecotoxicology and Environmental Safety. 2016;128:67-74.
7. Massa NR, Guangyun M, Xingyou Z, Xiumei H, Zhu C, Sampankanpanich SC, et al. Intrauterine Inflammation and Maternal Exposure to Ambient PM2.5 during Preconception and Specific Periods of Pregnancy: The Boston Birth Cohort. Environ Health Perspect. 2016;124(10):1608-15.
8. Vecoli C, Montano L, Andreassi MG. Environmental pollutants: genetic damage and epigenetic changes in male germ cells. Environmental Science and Pollution Research. 2016;23:23339-48.
9. Marcho C, Oluwayiose OA, Pilsner JR. The preconception environment and sperm epigenetics. Andrology. 2020;8(4):924-42.
10. Udagawa O, Furuyama A, Imai K, Fujitani Y, Hirano S. Effects of diesel exhaust-derived secondary organic aerosol (SOA) on oocytes: Potential risks to meiotic maturation. Reproductive Toxicology. 2018;75:56-64.
11. Zhang J-Y, Wu Q-J, Huang Y-H, Li J, Liu S, Chen Y-L, et al. Association between maternal exposure to ambient PM10 and neural tube defects: a case-control study in Liaoning Province, China. International Journal of Hygiene and Environmental Health. 2020;225:113453.
12. Li J, Liao J, Hu C, Bao S, Mahai G, Cao Z, et al. Preconceptional and the first trimester exposure to PM2. 5 and offspring neurodevelopment at 24 months of age: Examining mediation by maternal thyroid hormones in a birth cohort study. Environmental Pollution. 2021;284:117133.
13. Jo H, Eckel SP, Chen J-C, Cockburn M, Martinez MP, Chow T, et al. Gestational diabetes mellitus, prenatal air pollution exposure, and autism spectrum disorder. Environment international. 2019;133:105110.
14. Raz R, Roberts AL, Lyall K, Hart JE, Just AC, Laden F, et al. Autism spectrum disorder and particulate matter air pollution before, during, and after pregnancy: a nested case–control analysis within the Nurses' Health Study II cohort. Environmental health perspectives. 2015;123(3):264-70.
15. Grineski S, Alexander C, Renteria R, Collins TW, Bilder D, VanDerslice J, et al. Trimester-specific ambient PM2.5 exposures and risk of intellectual disability in Utah. Environmental Research. 2023;218:115009.
16. Blanc N, Liao J, Gilliland F, Zhang JJ, Berhane K, Huang G, et al. A systematic review of evidence for maternal preconception exposure to outdoor air pollution on Children's health. Environmental Pollution. 2022:120850.
17. Su X, Zhang S, Lin Q, Wu Y, Yang Y, Yu H, et al. Prenatal exposure to air pollution and neurodevelopmental delay in children: A birth cohort study in Foshan, China. Science of The Total Environment. 2022;816:151658.
18. Chen B, Huang S, He J, He Q, Chen S, Liu X, et al. Sex-specific influence of prenatal air pollutant exposure on neonatal neurobehavioral development and the sensitive window. Chemosphere. 2020;254:126824.

For peer review only - http://bmjopen.bmj.com/site/about/guidelines.xhtml

19. Wang P, Zhao Y, Li J, Zhou Y, Luo R, Meng X, et al. Prenatal exposure to ambient fine particulate matter and early childhood neurodevelopment: A population-based birth cohort study. Science of The Total Environment. 2021;785:147334.
20. Lei X, Zhang Y, Wang Z, Lu Z, Pan C, Zhang S, et al. Effects of prenatal exposure to PM2.5 and its composition on cognitive and motor functions in children at 12 months of age: The Shanghai Birth Cohort Study. Environment International. 2022;170:107597.
21. Huang S, Mo T-T, Norris T, Sun S, Zhang T, Han T-L, et al. The CLIMB (Complex Lipids In Mothers and Babies) study: protocol for a multicentre, three-group, parallel randomised controlled trial to investigate the effect of supplementation of complex lipids in pregnancy, on maternal ganglioside status and subsequent cognitive outcomes in the offspring. BMJ open. 2017;7(10):e016637.
22. Harper A, Baker PN, Xia Y, Kuang T, Zhang H, Chen Y, et al. Development of spatiotemporal land use regression models for PM2. 5 and NO2 in Chongqing, China, and exposure assessment for the CLIMB study. Atmospheric Pollution Research. 2021;12(7):101096.
23. Volk HE, Perera F, Braun JM, Kingsley SL, Gray K, Buckley J, et al. Prenatal air pollution exposure and neurodevelopment: A review and blueprint for a harmonized approach within ECHO. Environ Res. 2021;196:110320.
24. Ha S, Yeung E, Bell E, Insaf T, Ghassabian A, Bell G, et al. Prenatal and early life exposures to ambient air pollution and development. Environmental research. 2019;174:170-5.
25. Bennet L, Walker DW, Horne RS. Waking up too early–the consequences of preterm birth on sleep development. The Journal of physiology. 2018;596(23):5687-708.
26. Chiu Y-HM, Hsu H-HL, Coull BA, Bellinger DC, Kloog I, Schwartz J, et al. Prenatal particulate air pollution and neurodevelopment in urban children: examining sensitive windows and sex-specific associations. Environment international. 2016;87:56-65.
27. Huang S, Mo T-T, Norris T, Sun S, Zhang T, Han T-L, et al. The CLIMB (Complex Lipids In Mothers and Babies) study: protocol for a multicentre, three-group, parallel randomised controlled trial to investigate the effect of supplementation of complex lipids in pregnancy, on maternal ganglioside status and subsequent cognitive outcomes in the offspring. BMJ Open. 2017;7(10):e016637.
28. Albert BB, Derraik JG, Xia Y-Y, Norris T, Zhang T, Han T-L, et al. Supplementation with milk enriched with complex lipids during pregnancy: a double-blind randomized controlled trial. Plos one. 2021;16(2):e0244916.
29. Zhao X, Xia Y, Zhang H, Baker PN, Norris T. Birth weight charts for a Chinese population: an observational study of routine newborn weight data from Chongqing. BMC Pediatr. 2019;19(1):426.
30. Shourong Y, Xuerong L, Zhiwei Y. The revising of the bayley scales of infant development (BSID) in China. Chin J Clin Psychol. 1993;2:71-5.
31. Chen Y-T, Zhang T, Chen C, Xia Y-Y, Han T-L, Chen X-Y, et al. Associations of early pregnancy BMI with adverse pregnancy outcomes and infant neurocognitive development. Scientific Reports. 2021;11(1):1-8.
32. Guxens M, Garcia-Esteban R, Giorgis-Allemand L, Forns J, Badaloni C, Ballester F, et al. Air pollution during pregnancy and childhood cognitive and psychomotor development: six European birth cohorts. Epidemiology. 2014:636-47.
33. Lertxundi A, Baccini M, Lertxundi N, Fano E, Aranbarri A, Martínez MD, et al. Exposure to fine particle matter, nitrogen dioxide and benzene during pregnancy and cognitive and psychomotor developments in children at 15 months of age. Environment international. 2015;80:33-40.
34. Porta D, Narduzzi S, Badaloni C, Bucci S, Cesaroni G, Colelli V, et al. Air pollution and cognitive development at age 7 in a prospective Italian birth cohort. Epidemiology. 2016;27(2):228-36.
35. Kim E, Park H, Hong Y-C, Ha M, Kim Y, Kim B-N, et al. Prenatal exposure to PM10 and NO2 and children's neurodevelopment from birth to 24 months of age: Mothers and Children's Environmental Health (MOCEH) study. Science of the Total Environment. 2014;481:439-45.

For peer review only - http://bmjopen.bmj.com/site/about/guidelines.xhtml

36. Lin C-C, Yang S-K, Lin K-C, Ho W-C, Hsieh W-S, Shu B-C, et al. Multilevel analysis of air pollution and early childhood neurobehavioral development. International journal of environmental research and public health. 2014;11(7):6827-41.
37. Yorifuji T, Kashima S, Diez MH, Kado Y, Sanada S, Doi H. Prenatal exposure to traffic-related air pollution and child behavioral development milestone delays in Japan. Epidemiology. 2016;27(1):57-65.
38. Tozzi V, Lertxundi A, Ibarluzea JM, Baccini M. Causal effects of prenatal exposure to PM2. 5 on child development and the role of unobserved confounding. International Journal of Environmental Research and Public Health. 2019;16(22):4381.
39. Hurtado-Díaz M, Riojas-Rodríguez H, Rothenberg SJ, Schnaas-Arrieta L, Kloog I, Just A, et al. Prenatal PM2. 5 exposure and neurodevelopment at 2 years of age in a birth cohort from Mexico city. International journal of hygiene and environmental health. 2021;233:113695.
40. Lertxundi A, Andiarena A, Martínez MD, Ayerdi M, Murcia M, Estarlich M, et al. Prenatal exposure to PM2.5 and NO2 and sex-dependent infant cognitive and motor development. Environmental Research. 2019;174:114-21.
41. Iglesias-Vázquez L, Binter A-C, Canals J, Hernández-Martínez C, Voltas N, Ambros A, et al. Maternal exposure to air pollution during pregnancy and child's cognitive, language, and motor function: ECLIPSES study. Environmental Research. 2022;212:113501.
42. Umezawa M, Onoda A, Korshunova I, Jensen AC, Koponen IK, Jensen KA, et al. Maternal inhalation of carbon black nanoparticles induces neurodevelopmental changes in mouse offspring. Particle and Fibre Toxicology. 2018;15:1-18.
43. Monk C, Lugo-Candelas C, Trumpff C. Prenatal developmental origins of future psychopathology: mechanisms and pathways. Annual review of clinical psychology. 2019;15:317-44.
44. Xu R, Zhong Y, Li R, Li Y, Zhong Z, Liu T, et al. Association between exposure to ambient air pollution and semen quality: A systematic review and meta-analysis. Science of The Total Environment. 2023;870:161892.
45. Braun JM, Messerlian C, Hauser R. Fathers matter: why it's time to consider the impact of paternal environmental exposures on children's health. Current epidemiology reports. 2017;4:46-55.
46. Lopuszanska U, Samardakiewicz M. The relationship between air pollution and cognitive functions in children and adolescents: a systematic review. Cognitive and Behavioral Neurology. 2020;33(3):157-78.

For peer review only

For peer review only - http://bmjopen.bmj.com/site/about/guidelines.xhtml

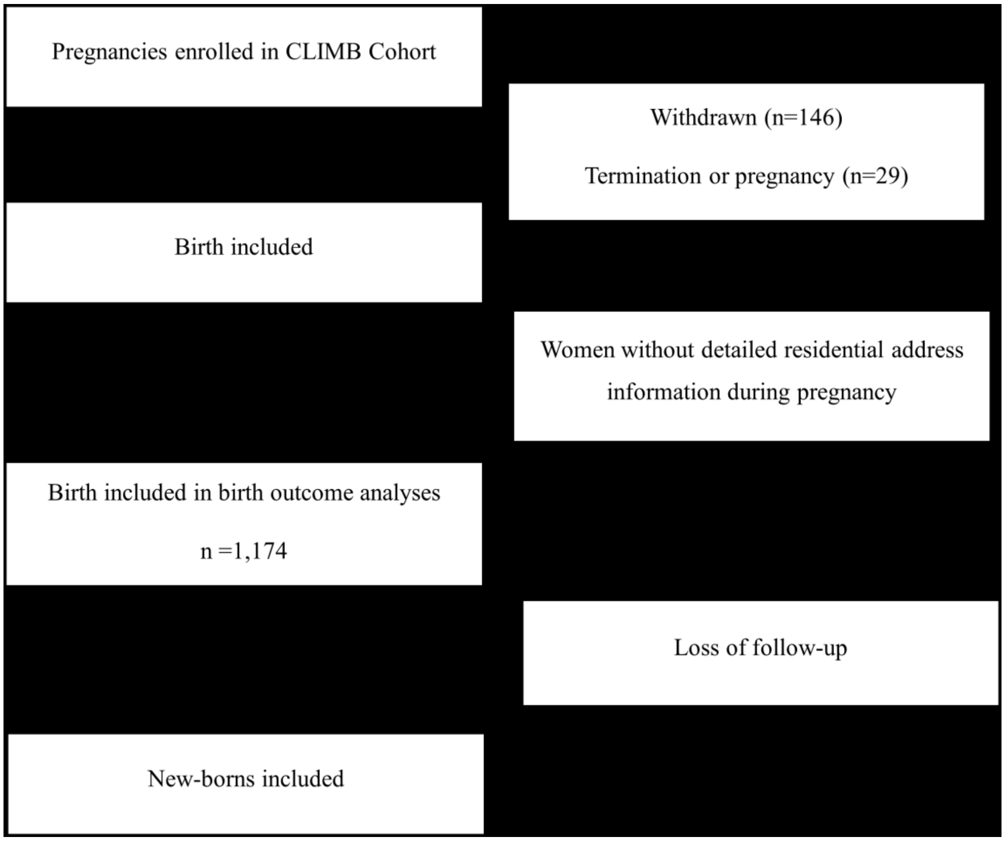

Figure 1 Flow chart of the study population in CLIMB

441x367mm (130 x 130 DPI)

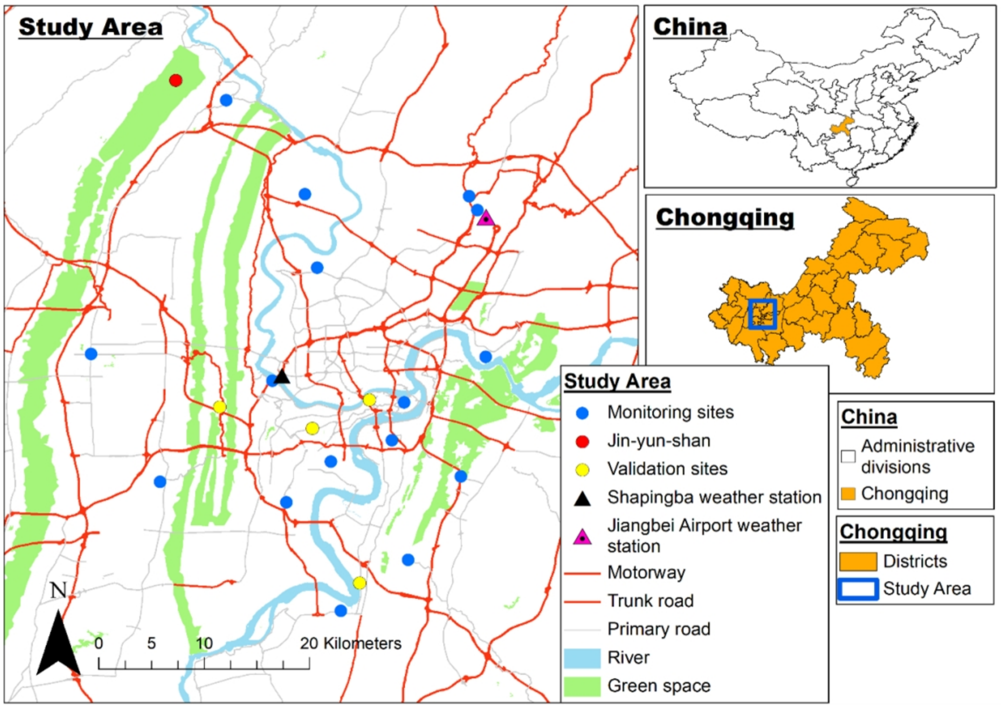

Figure 2 Study area and location of monitoring sites (OpenStreetMap contributors, 2015; https://data.nextgis.com/en/region/CN-50/).

402x284mm (87 x 87 DPI)

Associations of early pregnancy air pollution with adverse birth outcomes and infant neurocognitive development in the Complex Lipids in Mothers and Babies (CLIMB) cohort

Yingxin Chen*[1,2], Tao Kuang*[3], Ting Zhang[4], Yutong Samuel Cai[1,2], John Colombo[5], Alex Harper[6], Ting-Li Han[7], Yinyin Xia[8], John Gulliver[9], Anna L Hansell[1,2,10], Hua Zhang[7], Philip N Baker[6,7]

1: Centre for Environmental Health and Sustainability, University of Leicester, Leicester, UK

2: The National Institute of Health Research (NIHR) Health Protection Research Unit (HPRU) in Environmental Exposure and Health at the University of Leicester, Leicester, UK

3: Department of public health and management, Zunyi Medical and Pharmaceutical College, Zunyi, 563000, Guizhou, China

4. Stomatological Hospital of Chongqing Medical University, Chongqing, 401147, China

5: Schiefelbusch Institute for Life Span Studies and Department of Psychology, University of Kansas, Lawrence, KS 66045, USA

6: College of Life Sciences, University of Leicester, Leicester, UK

7: Department of Obstetrics and Gynaecology, the First Affiliated Hospital of Chongqing Medical University, Chongqing, 400016, China

8: School of Public Health, Chongqing Medical University, Chongqing, 400016, China

9: Environmental and Exposure Sciences, Population Health Research Institute, St George's, University of London, London, UK

10: NIHR Leicester Biomedical Research Centre, Leicester General Hospital, Leicester, UK

Correspondence to: Yinyin Xia. School of Public Health, Chongqing Medical University, Chongqing, 400016, China. Email: 100118@cqmu.edu.cn

*: Contributed equally

For peer review only

Supplement

eTable 1 Mental Development Index (Chinese version)

智力量表

（※可偶尔观察到）

序号	月龄	条目	计分
1	0.1	对铃声反应	
2	0.1	抱起时安静	
3	0.1	对摇鼓声反应	
4	0.1	对尖声反应：（电灯开关）	
5	0.1	短暂地注视红环	
6	0.2	短暂地注视人	
7	0.4	稍长时间地注视红环	
8	0.5	眼的水平协调活动（红环）	
9	0.7	眼的水平向天活动（光）	
10	0.7	眼睛追随移动的人	
11	0.7	对说话声反应	
12	0.8	眼的垂直协调活动（光）	
13	0.9	发声一至两次	
14	1	眼的垂直协调活动（红环）	
15	1.2	眼的旋转协调活动（光）	
16	1.2	眼的旋转细条活动（光环）	

For peer review only

17	1.3	※自由环视周围	
18	1.5	社交笑：测试者谈话与微笑时	
19	1.6	眼转向红环	
20	1.6	眼转向光	
21	1.6	※发声至少四次	
22	1.7	期待性兴奋	
23	1.7	对面部的纸有反应	
24	1.9	能用视觉辨认母亲	
25	1.9	社交笑：测试者微笑与安静时	
26	2	※对测试者的微笑和说话有发声反应	
27	2.1	※用眼睛寻找声源（详细说明）	
28	2.2	※发出两种不同的声音	
29	2.2	对手的遮蔽眨眼	
30	2.2	对面孔的消失有反应	
31	2.4	注视方木	
32	2.6	从一物转看另一物	
33	2.6	眼睛追随铅笔	
34	2.7	对抱起有预感性的调节反应	
35	2.9	目光追随横过桌面的球	
36	2.9	头追随悬摆的环	
37	3.1	头追随逐渐消失的勺子	
38	3.2	操作红环	
39	3.3	简单地玩摇鼓	

For peer review only

40	3.4	※轻轻地抚摸桌沿	
41	3.4	※意识到陌生环境	
42	3.5	头转向铃声	
43	3.6	头转向摇鼓声	
44	3.6	※手碰手的玩耍	
45	3.6	将红环送入口中	
46	3.7	伸手够悬环	
47	3.8	看自己的手	
48	4.2	接近悬环（优势手）	
49	4.4	※发声时的姿态（描述）	
50	4.4	※主动抚摸桌沿	
51	4.4	接近镜像	
52	4.4	注意小糖丸	
53	4.6	伸手取方木	
54	4.7	喜欢嬉戏	
55	4.9	伸手时眼手协调	
56	4.9	拾起方木（优势手）	
57	5	保持两块方木	
58	5	持久地看红环	
59	5	头部跟着掉下的勺转动	
60	5	探索性地玩纸	
61	5	对镜像微笑	
62	5	坚持够东西	

For peer review only

63	5.1	在小床内重新找到摇鼓	
64	5.1	※辨别生人	
65	5.4	举起倒扣的茶杯	
66	5.5	※敲打玩耍	
67	5.5	探索性地玩细绳	
68	5.5	伸手取第二块方木	
69	5.6	※由一手向另一手传递物体	
70	5.8	※对产生响声感兴趣	
71	5.9	灵巧而直接地拾起方木	
72	6	※对镜像开玩笑	
73	6	用把柄举起茶杯	
74	6	寻找掉落的勺子	
75	6.1	牵拉细绳获取红环	
76	6.1	保留三块方木中的两块	
77	6.6	※发出四个不同的音节	
78	6.8	能配合玩游戏	
79	7	恰当地牵拉细绳获取红环	
80	7.1	玩摇铃，对细节感兴趣	
81	7.4	企图获得三块方木	
82	7.4	有目的地摇铃	
83	7.5	※选择性地倾听熟悉的词语	
84	8	※对 da-da 或类同词	
85	8.1	暴露玩具	

For peer review only

86	8.2	注意测试者的乱写	
87	8.3	将手指插入桩板洞中	
88	8.6	观看书中图画	
89	8.9	对他人的言语要求有反应	
90	9.1	拿起茶杯获得方木	
91	9.8	寻找盒子里面的东西	
92	10.3	遵照命令将方木放入茶杯（放入数）	
93	10.7	企图模仿乱写	
94	10.8	模仿用勺子搅拌	
95	10.9	遵照命令停止	
96	10.9	推动小汽车	
97	11	模仿地拍打哨娃	
98	11.1	※重复引入发笑的把戏	
99	11.2	解开裹着的方木	
100	11.2	将三块方木放入杯中	
101	11.4	※快速而不清的表达	
102	11.4	揭开兰盒子的盖	
103	11.5	翻开书页	
104	11.5	摇晃悬环的	
105	11.8	将骰子放入盒中（6 个）	
106	12	恰当地握持画笔	
107	12.2	模仿说单词（记录用过的词）	
108	12.4	重复地插一根桩钉	

For peer review only

109	12.5	用手势表达想要的东西	
110	12.9	自动乱写	
111	12.9	能说两个词	
112	13	搭两层塔	
113	13.1	出示鞋子或其他衣服或自己的玩具	
114	13.2	从瓶中移出小糖丸	
115	13.3	掺九块方木放入杯中	
116	14.3	※盖上圆盒	
117	14.4	兰色模板：放置一个圆形模块	
118	14.8	用棍子够取玩具	
119	15.4	搭三层塔	
120	15.7	在 70 秒钟内插完桩钉	
121	16.1	指出娃娃身体的各部分：三个部位以上	
122	16.3	粉红模板：放置圆形模块	
123	16.6	兰色模板：放置两个圆形模块	
124	17.2	用笔模仿画一划	
125	17.5	在 42 秒钟内插完桩钉	
126	17.6	说出一物名	
127	17.7	对娃娃执行指令（在通过部位打钩：椅、杯、鼻）	
128	18.1	用语言表达要求	
129	18.6	不用于一划的乱写？	
130	18.8	兰色模板：放置两个圆快和方块	
131	18.8	指出三幅画	

For peer review only

132	19.1	能说两个单词的句子	
133	19.2	说出一副画名	
134	19.2	说出两幅画名	
135	19.3	找出两物	
136	19.8	在 30 秒钟内插完桩钉	
137	20.4	粉红模板：完成	
138	20.4	搭六层塔	
139	20.5	兰色模板，放置六个模块	
140	21	指出五副画	
141	21.1	说出三物名	
142	21.2	勉强合格地安装破娃娃	
143	21.2	区别两物：杯、盘、盒	
144	22.8	辨认钟表：第四张图 1，2，3，4，5	
145	22.9	说出三幅画名	
146	23.8	粉红模板（反转）	
147	24.3	近似地安装破娃娃	
148	24.6	区别三物：杯、盘、盒	
149	24.7	兰色模板，在 150 秒钟内完成	
150	25	搭八层塔	
151	25.1	指出七副画	
152	25.1	用方木搭火车	
153	25.7	说出五副画名	
154	26.3	模仿笔划：垂直线和水平线	

For peer review only

155	27.1	辨认钟表：第 2 张图	
156	27.6	理解两个方位词	
157	28	在 22 秒钟内插完桩钉	
158	28.5	兰色模板：90 秒钟内完成	
159	29.5	折纸	
160	29.6	兰色模板：60 秒钟内完成	
161	30+	正确安装破娃娃	
162	30+	“—”的概念	
163	30+	理解三个方位词	

For peer review only

eTable 2 Psychomotor Development Index (Chinese version)

运动量表

（※可偶尔观察到，▵可在施测智力量表时观察到）

序号	月龄	条目	计分
1	0.1	抱起靠肩时抬头	
2	0.1	抱起靠肩时调整姿势	
3	0.1	侧头	
4	0.1	爬起	
5	0.8	▵保留红环	
6	0.8	※伸臂玩耍	
7	0.8	※踢腿玩耍	
8	0.8	头起竖起：垂直位	
9	1.6	头部稳定地竖起	
10	1.7	抬头（背悬位）	
11	1.8	由侧卧转向仰卧	
12	2.2	在俯卧位时用双臂撑起自己	
13	2.2	支撑下坐起	
14	2.5	保持头部稳定	
15	2.6	※双手张开占优势	
16	3.3	头平衡	
17	3.4	※尺侧一手掌抓握方木	

For peer review only

18	3.5	轻度支撑坐位	
19	4.3	※由仰卧转向侧卧	
20	4.7	努力想坐起	
21	5.0	部分的拇指相对（桡侧一手掌）拾起方木	
22	5.1	独坐片刻	
23	5.1	※单手抽取	
24	5.2	※转腕	
25	5.2	牵拉坐起	
26	5.6	△试图获取小糖丸	
27	5.7	独立 30 秒钟或以上	
28	5.8	由仰卧转向俯卧	
29	6.2	稳定地独坐	
30	6.5	独坐时协调好	
31	6.6	※舀起小糖丸	
32	6.6	△完全的拇指相对拾起方木	
33	7	早期跨步运动	
34	7.5	牵拉站起	
35	7.6	※不完全的拇指相对抓糖丸	
36	7.6	走路之前的行进方式（俯卧、手膝、手足、其他）	
37	8.3	使两个勺子或方木在中线相碰	
38	8.5	跨步运动	
39	8.6	自己坐起	
40	8.6	借助家具站起	

For peer review only

41	8.9	精细地抓糖丸（灵巧地钳夹）	
42	9.6	拍手（中线技巧）	
43	9.8	坐下	
44	10	扶助下行走	
45	11.1	独站	
46	12	投球	
47	12.1	独走	
48	12.4	起立 I	
49	13.2	扶助下右足独站	
50	13.7	扶助下左足独站	
51	14.1	侧身走	
52	14.5	扶助上楼梯	
53	14.7	倒退走	
54	15.1	扶助下楼梯	
55	17.6	试图站在行木上	
56	18.7	左足独站	
57	19.3	单足踏在行木上走	
58	19.9	起立 II	
59	20.1	右足独站	
60	21.1	走直线：大致方向	
61	23.1	行木：双足站立	
62	24	踮脚走几步	
63	24.3	独自上楼梯：双足	

For peer review only

64	24.4	双足跳离地面	
65	25.3	独自下楼梯	
66	25.6	行木：企图跨步	
67	25.6	倒行两米半	
68	25.7	自第一级台阶下跳下	
69	29.2	自第二级台阶下跳下	
70	29.8	踮脚走两米半	
71	29.9	跳远：10 至 35cm（记录距离）	
72	30+	起立：III	
73	30+	上楼梯：双足交替向前	
74	30+	行木：交替步伐走部分路程	
75	30+	保持双足走在直线上（两米半）	
76	30+	跳远：35cm 至 60cm	
77	30+	跳过：5cm 高的绳子	
78	30+	跳远：60cm 至 85cm	
79	30+	独脚跳两次以上	
80	30+	下楼梯：双足交替向前	
81	30+	跳过 20cm 高的绳子	

For peer review only

eTable 3 Distributions of PM2.5 and NO2 exposure level in 90 days prior to conception, each trimester (T1, T2, and T3) and combined across whole pregnancy period (WP) (n = 1,174)

	Estimated exposure (μg/m³)						
	N	Minimum	25th percentile	Mean ± SD	Median ± IQR	75th percentile	Maximum
Estimated exposure to $PM_{2.5}$							
90 days prior to conception	1,174	38.17	44.00	52.91 ± 10.99	48.43 ± 18.07	62.06	80.53
First trimester	1,174	37.26	43.77	52.07 ± 10.98	47.26 ± 17.31	61.08	82.41
Second trimester	1,174	38.46	47.57	58.64 ± 12.21	57.97 ± 19.62	67.19	90.02
Third trimester	1,174	37.03	47.25	61.83 ± 16.04	58.82 ± 28.7	75.95	96.48
Total pregnancy	1,174	46.69	54.85	57.48 ± 3.97	57.31 ± 5.76	60.61	66.98
Estimated exposure to NO_2							
90 days prior to conception	1,174	25.86	45.49	49.59 ± 6.34	49.94 ± 8.27	53.76	70.48
First trimester	1,174	20.81	44.60	48.8 ± 6.43	48.92 ± 8.51	53.10	69.31
Second trimester	1,174	28.93	47.18	50.98 ± 6.23	51.20 ± 7.72	54.90	70.42
Third trimester	1,174	20.57	47.20	51.79 ± 6.78	52.45 ± 9.47	56.67	75.12
Total pregnancy	1,174	27.50	47.89	50.52 ± 5.08	50.46 ± 5.51	53.40	67.53

For peer review only

eTable 4 Pearson's correlations of $PM_{2.5}$ and NO_2 between each of the five different pregnancy time periods (N = 1,174)

Estimated exposure to		$PM_{2.5}$					NO_2				
		90 days prior to conception	First trimester	Second trimester	Third trimester	Total pregnancy	90 days prior to conception	First trimester	Second trimester	Third trimester	Total pregnancy
$PM_{2.5}$	90 days prior to conception	1									
	First trimester	-0.065	1								
	Second trimester	-0.779	-0.2012	1							
	Third trimester	0.288	-0.7613	-0.1688	1						
	Total pregnancy	-0.534	-0.2709	0.6838	0.3858	1					
NO_2	90 days prior to conception	0.6383	0.0684	-0.4588	0.3714	0.0376	1				
	First trimester	0.1537	0.6352	-0.0159	-0.4927	-0.0633	0.5545	1			
	Second trimester	-0.431	0.0714	0.6269	-0.0133	0.7251	0.3345	0.5399	1		
	Third trimester	0.3027	-0.5213	0.0528	0.6817	0.4432	0.7149	0.2159	0.5145	1	
	Total pregnancy	0.0057	0.0781	0.2862	0.0737	0.4779	0.6786	0.7435	0.8755	0.7331	1

For peer review only

eTable 5 Associations between $PM_{2.5}$ and NO_2 exposure in different pregnancy periods and adverse birth outcomes (unadjusted models)

		Mean difference		Odd ratios			
		Birth weight, grams	Birth length, cm	PTB (case: 33)	LBW (case: 30)	LGA (case: 108)	SGA (case: 84)
		99% CI	99% CI	99% CI	99% CI	99% CI	99% CI
	Per IQR increase in	(N=1,165)	(N=1,149)	(N=1,174)	(N=1,174)	(N=1,174)	(N=1,174)
Estimated exposure to $PM_{2.5}$	90 days prior to conception	9.28 (-44.04, 62.60)	-0.09 (-0.32, 0.14)	0.98 (0.46, 2.09)	1.35 (0.64, 2.82)	1.2 (0.79, 1.81)	0.98 (0.60, 1.58)
	First trimester	21.95 (-29.14, 73.04)	0.14 (-0.09, 0.36)	0.98 (0.48, 2.02)	1 (0.47, 2.12)	0.97 (0.64, 1.47)	0.78 (0.48, 1.27)
	Second trimester	-18.21 (-70.25, 33.84)	0.04 (-0.19, 0.27)	0.85 (0.40, 1.79)	0.61 (0.26, 1.40)	0.92 (0.60, 1.40)	1.33 (0.84, 2.10)
	Third trimester	-37.38 (-95.31, 20.56)	**-0.32 (-0.57, -0.07)**	1.35 (0.61, 2.99)	1.51 (0.66, 3.47)	1.08 (0.68, 1.72)	1.12 (0.67, 1.88)
	Total pregnancy	-20.02 (-66.93, 26.89)	-0.1 (-0.30, 0.10)	0.81 (0.42, 1.55)	0.69 (0.35, 1.37)	1.00 (0.69, 1.46)	1.2 (0.78, 1.84)
Estimated exposure to NO_2	90 days prior to conception	-13.23 (-55.66, 29.19)	-0.12 (-0.31, 0.06)	1.2 (0.65, 2.19)	1.62 (0.85, 3.10)	1.21 (0.85, 1.70)	1.24 (0.84, 1.83)
	First trimester	0.3 (-42.65, 43.25)	0.08 (-0.11, 0.27)	1.01 (0.55, 1.85)	1.15 (0.61, 2.17)	1.17 (0.83, 1.66)	1.27 (0.86, 1.87)
	Second trimester	-22.85 (-63.02, 17.32)	-0.04 (-0.22, 0.13)	1.11 (0.63, 1.95)	1.08 (0.60, 1.95)	1.06 (0.77, 1.47)	**1.46 (1.01, 2.11)**
	Third trimester	-32.72 (-78.00, 12.57)	-0.16 (-0.36, 0.03)	1.13 (0.60, 2.16)	1.35 (0.68, 2.69)	1.24 (0.86, 1.80)	**1.58 (1.03, 2.42)**
	Total pregnancy	-16.58 (-51.79, 18.63)	-0.03 (-0.18, 0.12)	1.03 (0.63, 1.69)	1.13 (0.67, 1.91)	1.16 (0.87, 1.55)	**1.44 (1.04, 2.00)**

For peer review only

eTable 6 Associations between $PM_{2.5}$ and NO_2 exposure in different pregnancy periods and adverse birth outcomes (co-exposure models)

		Mean difference		Odd ratios			
		Birth weight, grams	Birth length, cm	PTB (case: 33)	LBW (case: 30)	LGA (case: 108)	SGA (case: 84)
		99% CI	99% CI	99% CI	99% CI	99% CI	99% CI
	Per IQR increase in	(N=941)	(N=927)	(N=945)	(N=945)	(N=945)	(N=945)
Estimated exposure to $PM_{2.5}$	90 days prior to conception	75.00 (-36.61, 186.61)	0.23 (-0.25, 0.71)	0.21 (0.04, 1.03)	0.41 (0.10, 1.71)	1.14 (0.43, 3.03)	1.18 (0.36, 3.87)
	First trimester	19.59 (-99.85, 139.04)	0 (-0.52, 0.52)	0.97 (0.17, 5.55)	0.66 (0.09, 4.94)	0.67 (0.25, 1.79)	0.73 (0.21, 2.61)
	Second trimester	-25.62 (-129.13, 77.90)	0.08 (-0.36, 0.53)	1.34 (0.25, 7.29)	0.94 (0.13, 6.73)	0.83 (0.34, 2.01)	0.69 (0.27, 1.75)
	Third trimester	13.77 (-99.47, 127.00)	-0.2 (-0.69, 0.29)	1.12 (0.20, 6.12)	0.94 (0.18, 4.99)	1 (0.38, 2.68)	0.57 (0.21, 1.56)
	Total pregnancy	21.13 (-54.55, 96.81)	0.02 (-0.31, 0.35)	0.73 (0.26, 2.07)	0.52 (0.18, 1.48)	0.98 (0.51, 1.88)	0.55 (0.27, 1.15)
Estimated exposure to NO_2	90 days prior to conception	-18.63 (-78.33, 41.07)	-0.09 (-0.35, 0.17)	1.24 (0.49, 3.11)	1.3 (0.52, 3.29)	1.27 (0.74, 2.16)	1.39 (0.79, 2.46)
	First trimester	-14.53 (-75.84, 46.78)	0.05 (-0.22, 0.31)	0.91 (0.36, 2.28)	1.14 (0.44, 2.98)	1.33 (0.78, 2.27)	1.70 (0.93, 3.11)
	Second trimester	-14.46 (-71.01, 42.09)	-0.08 (-0.32, 0.17)	1.22 (0.51, 2.91)	1.36 (0.55, 3.36)	1.27 (0.77, 2.10)	1.50 (0.88, 2.55)
	Third trimester	-13.13 (-81.19, 54.93)	0.04 (-0.25, 0.34)	0.77 (0.28, 2.13)	0.97 (0.34, 2.78)	1.41 (0.78, 2.57)	1.77 (0.92, 3.40)
	Total pregnancy	-15.02 (-60.15, 30.11)	0 (-0.20, 0.19)	1.08 (0.55, 2.12)	1.28 (0.64, 2.58)	1.21 (0.81, 1.80)	**1.60 (1.03, 2.48)**

Models adjusted for maternal age at enrolment, infant's sex, maternal BMI at 11–14 weeks' gestation, primiparity, monthly household income level, and season of births.

For peer review only

For peer review only

eTable 7 Associations between $PM_{2.5}$ and NO_2 exposure in different pregnancy periods and BSID scores (unadjusted models)

		Mean difference	
		MDI 99% CI	PDI 99% CI
	Per IQR increase in	(N=946)	(N=946)
Estimated exposure to $PM_{2.5}$	90 days prior to conception	**-3.54 (-5.94, -1.13)**	**-3.42 (-5.44, -1.40)**
	First trimester	-1.07 (-3.51, 1.37)	0.04 (-2.01, 2.10)
	Second trimester	**4.21 (1.87, 6.56)**	**2.63 (0.65, 4.61)**
	Third trimester	-1.43 (-4.03, 1.17)	-1.76 (-3.94, 0.42)
	Total pregnancy	1.64 (-0.43, 3.71)	0.5 (-1.24, 2.25)
Estimated exposure to NO_2	90 days prior to conception	-1.90 (-3.82, 0.02)	**-2.86 (-4.46, -1.26)**
	First trimester	-0.08 (-2.05, 1.90)	-1.17 (-2.83, 0.48)
	Second trimester	1.81 (-0.03, 3.66)	0 (-1.56, 1.55)
	Third trimester	0.04 (-2.04, 2.11)	**-1.97 (-3.70, -0.23)**
	Total pregnancy	0.67 (-0.95, 2.28)	-1.08 (-2.44, 0.28)

For peer review only

eTable 8 Associations between $PM_{2.5}$ and NO_2 exposure in different pregnancy periods and BSID scores (co-exposure models)

		Mean difference	
		MDI 99% CI	PDI 99% CI
Per IQR increase in		(N=946)	(N=946)
Estimated exposure to $PM_{2.5}$	90 days prior to conception	-1.73 (-6.43, 2.98)	**-4.74 (-8.67, -0.81)**
	First trimester	-2.84 (-7.85, 2.18)	-0.45 (-4.68, 3.77)
	Second trimester	4.19 (-0.15, 8.53)	**5.51 (1.86, 9.16)**
	Third trimester	-3.84 (-8.60, 0.93)	0.04 (-3.99, 4.06)
	Total pregnancy	-0.85 (-4.04, 2.33)	1.69 (-0.99, 4.37)
Estimated exposure to NO_2	90 days prior to conception	-0.31 (-2.83, 2.20)	-1.72 (-3.82, 0.38)
	First trimester	1.28 (-1.30, 3.86)	-1.80 (-3.98, 0.37)
	Second trimester	-0.48 (-2.85, 1.90)	**-2.11 (-4.11, -0.12)**
	Third trimester	1.52 (-1.34, 4.38)	-1.92 (-4.34, 0.49)
	Total pregnancy	0.67 (-1.23, 2.57)	**-1.68 (-3.28, -0.08)**

Models adjusted for maternal age at enrolment, infant’s sex, maternal BMI at 11–14 weeks' gestation, primiparity, monthly household income level, and season of births.

For peer review only

eTable 9 Mediation effect of SGA on the associations between $PM_{2.5}$ and NO_2 exposure in different pregnancy periods and MDI scores

		Total Effect	P value	Direct Effect	P value	Indirect Effect	P value
Estimated exposure to PM2.5	90 days prior to conception	-3.54	0.000	-3.543	0.000	0.007	0.745
	First trimester	-1.07	0.259	-1.083	0.253	0.013	0.715
	Second trimester	4.21	0.000	4.239	0.000	0.024	0.599
	Third trimester	-1.43	0.157	-1.422	0.158	-0.006	0.779
	Total pregnancy	1.64	0.042	1.65	0.040	-0.012	0.695
Estimated exposure to NO2	90 days prior to conception	-1.90	0.011	1.896	0.011	0.007	0.799
	First trimester	-0.08	0.921	0.067	0.930	0.009	0.744
	Second trimester	1.81	0.011	1.844	0.010	-0.03	0.596
	Third trimester	0.04	0.962	0.059	0.942	-0.021	0.728
	Total pregnancy	0.67	0.288	0.686	0.274	-0.021	0.672

For peer review only

eTable 10 Mediation effect of SGA on the associations between $PM_{2.5}$ and NO_2 exposure in different pregnancy periods and PDI scores

		Total Effect	P value	Direct Effect	P value	Indirect Effect	P value
Estimated exposure to PM2.5	90 days prior to conception	-3.422	0.000	-3.421	0.000	0.001	0.931
	First trimester	0.045	0.955	0.049	0.951	-0.005	0.872
	Second trimester	2.632	0.001	2.631	0.001	0.000	0.997
	Third trimester	-1.758	0.038	-1.762	0.037	0.003	0.849
	Total pregnancy	0.504	0.456	0.501	0.458	0.003	0.894
Estimated exposure to NO2	90 days prior to conception	-2.862	0.000	-2.869	0.000	0.007	0.762
	First trimester	-1.174	0.067	-1.179	0.066	0.005	0.826
	Second trimester	-0.003	0.996	-0.010	0.986	0.007	0.872
	Third trimester	-1.966	0.004	-1.985	0.003	0.018	0.711
	Total pregnancy	-1.079	0.041	-1.092	0.038	0.013	0.751

For peer review only

eTable 11 Comparison of major confounders and outcomes for those with missing and non-missing outcome data and non-missing confounder data

	Excluded from analyses due to missing cognitive outcome data (n=256)	Included in analyses (n = 1,017)	p-value for difference
**Maternal age, mean ± SD**	28.47 ± 0.22	28.78 ± 0.11	0.22
**(week), mean ± SD**	39.34 ± 0.12	39.39 ± 0.04	0.69
**Maternal BMI (kg/m²), mean ± SD**	21.13 ± 0.17	21.56 ± 0.09	**0.03**
**Infant's sex**			
Female, n (%)	136 (53.33)	527 (51.87)	0.676
Male, n (%)	119 (46.67)	489 (48.13)	
**Primiparity**			0.583
Yes, n (%)	226 (22.22)	791 (77.78)	
No, n (%)	61 (23.83)	195 (76.17)	
**Season**			**0.00**
Spring (Mar-May), n (%)	74 (32.46)	337 (35.62)	
Summer (Jun-Aug), n (%)	74 (32.46)	189 (19.98)	
Autumn (Sep-Nov), n (%)	55 (24.12)	143 (15.12)	
Winter (Dec-Feb), n (%)	25 (10.96)	277 (29.28)	

For peer review only

Reporting checklist for cohort study.

Based on the STROBE cohort guidelines.

Instructions to authors

Complete this checklist by entering the page numbers from your manuscript where readers will find each of the items listed below.

Your article may not currently address all the items on the checklist. Please modify your text to include the missing information. If you are certain that an item does not apply, please write "n/a" and provide a short explanation.

Upload your completed checklist as an extra file when you submit to a journal.

In your methods section, say that you used the STROBE cohortreporting guidelines, and cite them as:

von Elm E, Altman DG, Egger M, Pocock SJ, Gotzsche PC, Vandenbroucke JP. The Strengthening the Reporting of Observational Studies in Epidemiology (STROBE) Statement: guidelines for reporting observational studies.

		Reporting Item	Page Number
**Title and abstract**			
Title	#1a	Indicate the study's design with a commonly used term in the title or the abstract	1

For peer review only

Abstract	#1b	Provide in the abstract an informative and balanced summary of what was done and what was found	2, 3
**Introduction**			
Background / rationale	#2	Explain the scientific background and rationale for the investigation being reported	4, 5
Objectives	#3	State specific objectives, including any prespecified hypotheses	5
**Methods**			
Study design	#4	Present key elements of study design early in the paper	5,6
Setting	#5	Describe the setting, locations, and relevant dates, including periods of recruitment, exposure, follow-up, and data collection	5,6
Eligibility criteria	#6a	Give the eligibility criteria, and the sources and methods of selection of participants. Describe methods of follow-up.	5,6
Eligibility criteria	#6b	For matched studies, give matching criteria and number of exposed and unexposed	6
Variables	#7	Clearly define all outcomes, exposures, predictors, potential confounders, and effect modifiers. Give diagnostic criteria, if applicable	6, 7, 8
Data sources / measurement	#8	For each variable of interest give sources of data and details of methods of assessment (measurement). Describe comparability of assessment methods if there is	6, 7, 8

For peer review only

		more than one group. Give information separately for for exposed and unexposed groups if applicable.	
Bias	#9	Describe any efforts to address potential sources of bias	6, 7, 8
Study size	#10	Explain how the study size was arrived at	6
Quantitative variables	#11	Explain how quantitative variables were handled in the analyses. If applicable, describe which groupings were chosen, and why	8, 9
Statistical methods	#12a	Describe all statistical methods, including those used to control for confounding	8, 9
Statistical methods	#12b	Describe any methods used to examine subgroups and interactions	8, 9
Statistical methods	#12c	Explain how missing data were addressed	8, 9
Statistical methods	#12d	If applicable, explain how loss to follow-up was addressed	8, 9
Statistical methods	#12e	Describe any sensitivity analyses	8, 9

Results

Participants	#13a	Report numbers of individuals at each stage of study—eg numbers potentially eligible, examined for eligibility,	10

For peer review only

		confirmed eligible, included in the study, completing follow-up, and analysed. Give information separately for for exposed and unexposed groups if applicable.	
Participants	#13b	Give reasons for non-participation at each stage	10, 20
Participants	#13c	Consider use of a flow diagram	10, 20
Descriptive data	#14a	Give characteristics of study participants (eg demographic, clinical, social) and information on exposures and potential confounders. Give information separately for exposed and unexposed groups if applicable.	10,22
Descriptive data	#14b	Indicate number of participants with missing data for each variable of interest	N/A
Descriptive data	#14c	Summarise follow-up time (eg, average and total amount)	N/A
Outcome data	#15	Report numbers of outcome events or summary measures over time. Give information separately for exposed and unexposed groups if applicable.	10,22
Main results	#16a	Give unadjusted estimates and, if applicable, confounder-adjusted estimates and their precision (eg, 95% confidence	10, 11

For peer review only

		interval). Make clear which confounders were adjusted for and why they were included	
Main results	#16b	Report category boundaries when continuous variables were categorized	10, 11
Main results	#16c	If relevant, consider translating estimates of relative risk into absolute risk for a meaningful time period	N/A
Other analyses	#17	Report other analyses done—eg analyses of subgroups and interactions, and sensitivity analyses	11
**Discussion**			
Key results	#18	Summarise key results with reference to study objectives	11, 12
Limitations	#19	Discuss limitations of the study, taking into account sources of potential bias or imprecision. Discuss both direction and magnitude of any potential bias.	14
Interpretation	#20	Give a cautious overall interpretation considering objectives, limitations, multiplicity of analyses, results from similar studies, and other relevant evidence.	12, 13
Generalisability	#21	Discuss the generalisability (external validity) of the study results	14
**Other Information**			

For peer review only

Funding	#22	Give the source of funding and the role of the funders for the present study and, if applicable, for the original study on which the present article is based	18

None The STROBE checklist is distributed under the terms of the Creative Commons Attribution License CC-BY. This checklist can be completed online using https://www.goodreports.org/, a tool made by the EQUATOR Network in collaboration with Penelope.ai

For peer review only

Associations of air pollution exposures in preconception and pregnancy with birth outcomes and infant neurocognitive development: analysis of the Complex Lipids in Mothers and Babies (CLIMB) prospective cohort in Chongqing, China

Journal:	*BMJ Open*
Manuscript ID	bmjopen-2023-082475.R1
Article Type:	Original research
Date Submitted by the Author:	25-Mar-2024

Complete List of Authors:	Chen, Yingxin; University of Leicester, Centre for Environmental Health and Sustainability<br>Kuang, Tao; Zunyi Medical and Pharmaceutical College, Department of public health and management<br>Harper, Alex; University of Leicester<br>Gulliver, John; St George's University of London<br>Zhang, Ting; Stomatological Hospital of Chongqing Medical University<br>Cai, Samuel; University of Leicester, Department of Health Sciences<br>Colombo, John ; University of Kansas<br>Han, Ting-Li; University of Auckland Liggins Institute; Canada - China - New Zealand Joint Laboratory of Maternal and Fetal Medicine<br>xia, yinyin; Chongqing Medical University<br>Hansell, Anna ; University of Leicester, Centre for Environmental Health and Sustainability<br>Zhang, Hua; The First Affiliated Hospital of Chongqing Medical University, Department of Obstetrics and Gynaecology<br>Baker, Philip ; University of Leicester, College of Medicine

**Primary Subject Heading**:	Epidemiology
Secondary Subject Heading:	Obstetrics and gynaecology
Keywords:	China, Developmental neurology & neurodisability < PAEDIATRICS, Child & adolescent psychiatry < PSYCHIATRY, EPIDEMIOLOGIC STUDIES, EPIDEMIOLOGY, Maternal medicine < OBSTETRICS

SCHOLARONE™
Manuscripts

I, the Submitting Author has the right to grant and does grant on behalf of all authors of the Work (as defined in the below author licence), an exclusive licence and/or a non-exclusive licence for contributions from authors who are: i) UK Crown employees; ii) where BMJ has agreed a CC-BY licence shall apply, and/or iii) in accordance with the terms applicable for US Federal Government officers or employees acting as part of their official duties; on a worldwide, perpetual, irrevocable, royalty-free basis to BMJ Publishing Group Ltd ("BMJ") its licensees and where the relevant Journal is co-owned by BMJ to the co-owners of the Journal, to publish the Work in this journal and any other BMJ products and to exploit all rights, as set out in our licence.

The Submitting Author accepts and understands that any supply made under these terms is made by BMJ to the Submitting Author unless you are acting as an employee on behalf of your employer or a postgraduate student of an affiliated institution which is paying any applicable article publishing charge ("APC") for Open Access articles. Where the Submitting Author wishes to make the Work available on an Open Access basis (and intends to pay the relevant APC), the terms of reuse of such Open Access shall be governed by a Creative Commons licence – details of these licences and which Creative Commons licence will apply to this Work are set out in our licence referred to above.

Other than as permitted in any relevant BMJ Author's Self Archiving Policies, I confirm this Work has not been accepted for publication elsewhere, is not being considered for publication elsewhere and does not duplicate material already published. I confirm all authors consent to publication of this Work and authorise the granting of this licence.

For peer review only

Associations of air pollution exposures in preconception and pregnancy with birth outcomes and infant neurocognitive development: analysis of the Complex Lipids in Mothers and Babies (CLIMB) prospective cohort in Chongqing, China

Yingxin Chen[1,2], Tao Kuang[3], Ting Zhang[4], Yutong Samuel Cai[1,2, 5], John Colombo[6], Alex Harper[7], Ting-Li Han[8], Yinyin Xia[9], John Gulliver[10], Anna L Hansell[1,2,5], Hua Zhang[8], Philip N Baker[7,8]

1: Centre for Environmental Health and Sustainability, University of Leicester, Leicester, UK

2: The National Institute of Health Research (NIHR) Health Protection Research Unit (HPRU) in Environmental Exposure and Health at the University of Leicester, Leicester, UK

3: Department of public health and management, Zunyi Medical and Pharmaceutical College, Zunyi, 563000, Guizhou, China

4. Stomatological Hospital of Chongqing Medical University, Chongqing, 401147, China

5: NIHR Leicester Biomedical Research Centre, Leicester General Hospital, Leicester, UK

6: Schiefelbusch Institute for Life Span Studies and Department of Psychology, University of Kansas, Lawrence, KS 66045, USA

7: College of Life Sciences, University of Leicester, Leicester, UK

8: Department of Obstetrics and Gynaecology, the First Affiliated Hospital of Chongqing Medical University, Chongqing, 400016, China

9: School of Public Health, Chongqing Medical University, Chongqing, 400016, China

10: Environmental and Exposure Sciences, Population Health Research Institute, St George's, University of London, London, UK

Correspondence to: Yinyin Xia. School of Public Health, Chongqing Medical University, Chongqing, 400016, China. Email: 100118@cqmu.edu.cn

For peer review only - http://bmjopen.bmj.com/site/about/guidelines.xhtml

Abstract

Objectives: to investigate the associations of traffic-related air pollution exposures in early pregnancy with birth outcomes and infant neurocognitive development

Design: cohort study

Setting: eligible women attended six visits in the maternity clinics of two centres (the First Affiliated Hospital of Chongqing Medical University and Chongqing Health Centre for Women and Children).

Participants: women who were between 20 and 40 years of age and were at 11–14 weeks gestation with a singleton pregnancy were eligible for participation. Women were excluded if they had a history of premature delivery before 32 weeks of gestation, maternal milk allergy or aversion, or severe lactose intolerance. 1,273 pregnant women enrolled in 2015-2016 and 1,174 live births were included in this analysis.

Exposures: Air pollution concentrations at their home addresses, including particulate matter (PM) with diameter ≤2.5μm ($PM_{2.5}$) and nitrogen dioxide (NO_2), during pre-conception and each trimester period were estimated using land-use regression models.

Outcome measures: birth outcomes (i.e., birthweight, birth length, preterm birth (PTB), low birth weight (LBW), large for gestational age (LGA) and small for gestational age (SGA) status) and neurodevelopment outcomes measured by the Chinese version of Bayley Scales of Infant Development (CBSID).

Results: An association between SGA and per Interquartile range (IQR) increases in NO_2 was found in the first trimester (Odd ratio (OR): 1.57, 95% confidence interval (CI): 1.06, 2.32) and during the whole pregnancy (OR: 1.33, 99% CI: 1.01, 1.75). Both $PM_{2.5}$ and NO_2 exposure in the 90 days prior to conception were associated with lower Psychomotor Development Index (PDI) scores (β: -6.15, 95% CI: -8.84, -3.46; β: -2.83, 95% CI: -4.27, -1.39, respectively). Increased NO_2 exposure was associated with an increased risk of psychomotor development delay (PDD) during different trimesters of pregnancy.

Conclusions: Increased exposure to NO_2 during pregnancy were associated with increased risk of SGA and psychomotor development delay, while increased exposure to both $PM_{2.5}$ and NO_2 pre-conception were associated with adverse psychomotor development outcomes at 12 months of age.

For peer review only - http://bmjopen.bmj.com/site/about/guidelines.xhtml

Keywords: Air pollution; birth outcomes; child cognition

For peer review only

For peer review only - http://bmjopen.bmj.com/site/about/guidelines.xhtml

Article summary

Strengths and limitations of this study

- We developed an LUR model to capture spatial and temporal variations of air pollution at individual level to reduce exposure misclassification.
- This study uniquely explored the impacts of both pre-conception and prenatal exposure to $PM_{2.5}$ and NO_2 on neurodevelopmental outcomes in young infants, within an urban environment characterized by relatively high air pollution levels.
- Our sample size was relatively small, limiting the statistical power to assess several outcomes.
- We defined exposure windows for clinically-defined trimesters.

For peer review only

For peer review only - http://bmjopen.bmj.com/site/about/guidelines.xhtml

Introduction

Air pollution is a major environmental factor that has been linked to a range of adverse health outcomes in children. Maternal exposure to air pollutants during pregnancy, especially particulate matter (PM) with diameter ≤2.5μm ($PM_{2.5}$) and nitrogen dioxide (NO_2), has been found to be associated with adverse birth outcomes, including pre-term birth (PTB)(1), term low birth weight (TLBW) (2), and small for gestational age (SGA) status (3). According to the developmental origins of health and disease (DOHaD) hypothesis, prenatal exposures to air pollution may lead to adverse birth outcomes and subsequently increase the susceptibility to the development of certain diseases later in life (4). A number of epidemiological studies have linked prenatal air pollution exposure with neurodevelopmental disorders such as autism spectrum disorder (ASD), attention deficit hyperactivity disorder (ADHD), and cognitive impairment (5). Although the underlying biological mechanisms are still unclear, some studies indicated that prenatal air pollution exposure may induce systemic oxidative stress that triggers intrauterine inflammation, leading to damage to several fetal organs, including the brain (6, 7).

It is also unclear that whether the adverse effects of air pollution may start earlier before conception. Three months before conception was considered as a critical developmental window for gametogenesis. Air pollution exposure during the three-month preconception period may have adverse effects on gametogenesis of sperm (8, 9) and ova cells (10). Exposures to $PM_{2.5}$ in preconception period have been associated with various neurodevelopmental outcomes, such as neural tube defects (11), lower psychomotor development scores (12), higher risk of ASD (13, 14), and higher risk of intellectual disability (15). Further research is required due to inconsistencies across studies in terms of studied health outcomes and exposure levels of air pollution (12). Additionally, while there is growing evidence for the effects of preconception $PM_{2.5}$ exposure on the risk of adverse neurodevelopmental outcomes, no study to date has examined the effects of preconception NO_2 exposure. Exposure to NO_2 during pregnancy may be linked to compromised neural development in children, particularly affecting fine psychomotor skills(16). Studying $PM_{2.5}$ along with NO_2 may allow us to explore how multiple pollutants affect birth outcomes and infant neurocognitive development independently and jointly. Moreover, both $PM_{2.5}$ and NO_2 are regulated traffic-related air pollutants in many countries. Understanding their impacts on birth and infant neurocognitive development can provide valuable insights for policymakers and public health authorities to develop effective air quality regulations and interventions.

For peer review only - http://bmjopen.bmj.com/site/about/guidelines.xhtml

Many studies have reported the effects of prenatal exposure to air pollution on neurodevelopmental function in children. However, the reported associations vary, due to the heterogeneous assessments of air pollution and neurodevelopmental outcomes (5, 17).

The current study leveraged the Complex Lipids in Mothers and Babies (CLIMB) cohort, a prospective birth cohort recruited in Chongqing, China (18), with trimester-specific maternal $PM_{2.5}$ and NO_2 air pollution exposure derived from a spatio-temporal land use regression (LUR) model (19). The aim of this analysis was to examine the associations between $PM_{2.5}$ and NO_2 exposures during pre- and during pregnancy, with birth and infant neurocognitive development outcomes at 12 months of age.

A key aspect in all studies like this one is the accuracy of documenting exposure; a recent Chinese study determined air pollution exposure based on data from the nearest monitoring station (20) may not reflect the fine temporal and spatial variability of pollutant exposures among participants. Our study employed common air pollution exposure models based on advanced geographic information systems (GIS), to address some of the limitations of previous studies (21).

In addition, the timing of exposure is also critical in determining the effects of exposure on developmental outcomes. Indeed, the evidence from previous studies on the sensitive time windows for exposure pre- and during pregnancy remains inconclusive. Some studies have indicated that the early-to-mid pregnancy phase may be a critical period in terms of the impact of air pollution on neurodevelopment(22, 23). Early pregnancy is particularly important for neurogenesis and neuromigration, making it a susceptible period (24). However, some studies reported stronger associations for middle or late pregnancy (20, 25, 26). More studies identifying critical periods are needed to enhance our understanding of how pre-conception and prenatal air pollution exposure affect neurodevelopment. With this cohort, we are able to examine the effects of exposure pre-conception, at each trimester, and the entire pregnancy.

For peer review only - http://bmjopen.bmj.com/site/about/guidelines.xhtml

Methods

Study population

Participant recruitment in the CLIMB cohort has been described previously (27). In brief, women who were between 20 and 40 years of age and were at 11–14 weeks gestation with a singleton pregnancy were eligible for participation. Women were excluded if they had a history of premature delivery before 32 weeks of gestation, maternal milk allergy or aversion, or severe lactose intolerance.

From September 2015 to November 2016, a total of 1,500 women were recruited into the cohort. Participants attended six visits at the First Affiliated Hospital of Chongqing Medical University and Chongqing Health Centre for Women and Children: 11–14 weeks' gestation (visit 1), 22–28 week's gestation (visit 2), 32–34 week's gestation (visit 3), at birth (visit 4), 6 weeks postnatal (visit 5), and 12 months postnatal (visit 6).

Women who withdrew from the study (n = 146), terminated their pregnancy (n = 29), miscarried (n = 12) or were lost to follow-up (n = 40) were excluded from the analysis, leaving a sample size of 1,273 women. Analyses were restricted to mothers whose detailed residential addresses during pregnancy were known (**Figure 1**). A total of 1,174 live births were thus included in the pregnancy and neonatal outcomes analysis. Subsequently, at 1 year follow-up, 946 children were included in the analysis of neurodevelopment outcomes.

Exposure assessment

The address of participants was collected at the first visit. Exposure assessment based on spatiotemporal land use regression (LUR) models for $PM_{2.5}$ and NO_2 were developed for the study region. The study area focused on the urban center of the Chinese municipality of Chongqing (**Figure 2**). A description of the methodology of exposure modelling has been reported previously (19). Briefly, the models included both spatial and temporal components of exposure. $PM_{2.5}$ and NO_2 concentration data were collected from 17 routine monitoring sites operated by the Chongqing Environmental Monitoring Center in 2015-2016. For the spatial component of models, we calculated annual average concentrations of each pollutant in 2015, and fit linear regression models using five groups of geographic data (road network, land use, topography, vegetation, and population density) as spatial predictor variables. For the temporal component of models, we calculated the residuals from the spatial component at each

For peer review only - http://bmjopen.bmj.com/site/about/guidelines.xhtml

monitoring site on a daily basis by subtracting the predicted annual average concentration from the observed daily average concentrations measured in 2015 and 2016, and then fitted generalised additive models (GAM) using seven groups of meteorological data (temperature, amount of rainfall, rainfall events, relative humidity, horizontal visibility, wind direction and wind speed) as temporal predictor variables. The meteorological variables were used to account for the influence of weather on the change in air pollution concentration over time. To account for the remaining spatial autocorrelation, the smoothed terms of longitude and latitude were fit to spatiotemporal residuals which were calculated by subtracting the sum of the spatial temporal predictions from the measured daily average concentrations in 2015 and 2016. The performance of the $PM_{2.5}$ spatiotemporal models was good (Correlation (COR)-R^2: 0.72) and the NO_2 spatiotemporal model was moderate (COR-R^2: 0.39) when providing concentration estimates in absolute terms.

Combining the family address coordinates of each pregnant woman and the gestation period of the pregnancy (calculated from the date of last menstrual period to the date of delivery), we used this spatiotemporal model to estimate the average exposure of each pregnant woman in 90 days prior to pregnancy (90D), first trimester (T1), second trimester (T2), third trimester (T3) and whole pregnancy period (WP), respectively.

Outcomes

Birth Outcomes

Birth outcomes were determined by experienced obstetricians and abstracted from the medical records. Birth outcomes included: birthweight (in grams), birth length (in centimetres), PTB, low birth weight (LBW), large for gestational age (LGA) and SGA status (28). PTB was defined as delivery before 37 weeks. LBW was defined as weighing less than 2500 g at birth. LGA and SGA were indicated by birth weight greater than and less than the 90th and 10th percentile within this study for the gestational age by sex respectively (29). Term low birth weight was not considered due to a small sample size of only 8 cases.

Neurodevelopment outcomes

For peer review only - http://bmjopen.bmj.com/site/about/guidelines.xhtml

The Chinese version of Bayley Scales of Infant Development (CBSID) was used to assess mental and psychomotor development for infants in this study. The CBSID is appropriate for evaluation of infants from 2–30 months old (30) and takes into consideration each infant's age in days. Infants were assessed at around 12 months (range from 11 months and 15 days to 12 months and 15 days) by a trained examiner, with ages corrected for preterm birth. These scales have been formally adapted to the Chinese language and locally standardized to become culturally appropriate, with two main indexes: the Mental Development Index (MDI) and the Psychomotor Development Index (PDI). The MDI component comprised 163 items and assessed age-appropriate items related to cognitive functioning, personal and social development, and language development (see **eTable 1** in the Supplement). The PDI component comprised 81 items and assessed age-appropriate fine and gross motor skills (see **eTable 2** in the Supplement). The test provided raw scores for mental and psychomotor development that were converted to standardized (in terms of age in days) MDI and PDI scores, based on norms for the Chinese population. As with other forms of the Bayley test these index scores have a mean of 100, and a standard deviation of 15, with a lower score reflecting poorer performance (31). If an infant refused to cooperate with the examiners to finish the task, a second assessment was arranged within two weeks. If the infant could not cooperate at the second BSID assessment, their data were classified as missing. In addition to the continuous scores, we define mental developmental delay (MDD) and psychomotor developmental delay (PDD) if the score is less than 85(32).

Covariates

Socio-demographic data were collected through interviews by trained nurses. The following potential confounders were identified: maternal age at enrolment (in years), infant sex (male/female), maternal BMI at 11–14 weeks' gestation (kg/m^2), parity (Yes/No), monthly household income level (categorized as: <2,000 yuan, 2,000–7,000 yuan, 7,000–10,000 yuan, or >10,000 yuan), season of birth (categorized as: Spring (Mar-May), Summer (Jun-Aug), Autumn (Sep-Nov) or Winter (Dec-Feb)). Marital status (single/married) and smoking or drinking during pregnancy (Yes/no) were not taken into account in this analysis because of the homogeneity of the study population (i.e., 98.6% women were married and 99.6% women reported not smoking or drinking alcohol during pregnancy). We did not adjust dietary

For peer review only - http://bmjopen.bmj.com/site/about/guidelines.xhtml

supplements during pregnancy because all pregnant women routinely take folic acid in this cohort.

Statistical analyses

Data were described in terms of mean ± SD or median (IQR) for continuous variables, or as percentages for categorical variables. Modelled $PM_{2.5}$ and NO_2 exposure levels in 90D, T1, T2, T3 and WP were considered separately. We examined the Spearman correlation between each of the exposures in the different pregnancy periods. For birth outcomes, multivariable linear regression was used for continuous outcomes (e.g., birth weight and birth length) to estimate β coefficient and their 95% confidence intervals (CIs) and multivariable logistic regression for binary outcomes (e.g., PTB, LBW, LGA and SGA status) to estimate odds ratio (OR) and 95% CIs. For mental and psychomotor development (e.g., MDI and PDI scores), multivariable linear regression models were fit to estimate β coefficient and their 95% CIs. We also conducted multivariable logistic regression analysis for binary neurodevelopment outcomes (i.e., MDD and PDD). Models were adjusted for maternal age at enrolment, infant sex, maternal BMI at 11–14 weeks gestation, primiparity, monthly household income level, and season of birth. We also ran co-exposure models to estimate associations of one air pollutant whilst additionally adjusting for the other air pollutant (i.e., $PM_{2.5}$ effects in T1 adjusted for NO_2 in T1). Effect estimates are reported for each IQR increase of $PM_{2.5}$ and NO_2. All analyses were performed using STATA version 17. A p-value of <0.05 was considered statistically significant to address multiple comparisons in the analyses.

Patient and public involvement

Patients and/or the public were not involved in the design, or conduct, or reporting, or dissemination plans of this research.

For peer review only - http://bmjopen.bmj.com/site/about/guidelines.xhtml

Results

Study Participants

Participant characteristics are presented in **Table 1**. Of those participating women, the mean age was 28.7 years and mean BMI was 21.5 kg/m^2. 98.0% of women were of Han ethnicity, 77.9% were primiparous, and 67.6% had completed tertiary education. 33 (2.8%), 30 (2.6%), 108 (9.2%), 84 (7.2%) of the 1,174 births considered in this analysis were classified as PTB, LBW, LGA and SGA, respectively. For those 946 children who completed the BSID test, the mean MDI score was 94.7 (SD: 17.7) and the mean PDI score was 87.4 (SD: 14.9). The proportions of participants with MDD (MDI <85) and PDD (PDI < 85) were 27.1% and 42.4%, respectively.

Exposure assessment

Median $PM_{2.5}$ exposure concentrations were 57.31 μg/m^3 (IQR: 5.76) and median NO_2 exposure levels were 50.46 μg/m^3 (IQR: 5.51) during the whole pregnancy period (**eTable 3 in the Supplement**). For $PM_{2.5}$, the concentration in the pre-conception and T1 were considerably lower than other periods, close to 10 μg/m^3. The between-trimester and 90D values for NO_2 were generally moderately correlated (Pearson's $r > 0.5$). The correlation coefficients of $PM_{2.5}$ were more variable between time periods reflecting the high variability of $PM_{2.5}$ concentrations, with values ranging from -0.78 to +0.68. Correlations between $PM_{2.5}$ and NO_2 in the same pregnancy period were moderately correlated (Pearson's $r \sim 0.6$, **eTable 4 in the Supplement**).

For peer review only - http://bmjopen.bmj.com/site/about/guidelines.xhtml

Table 1 Characteristics of study sample in the CLIMB cohort (N = 1,174)

Characteristic of mothers	N	n (%) / mean ± SD	Characteristic of child	N	n (%) /mean ± SD
Maternal age (Years)	1,174	28.7 ± 3.5	Gestational week (week)	1,174	39.4 ± 1.5
BMI (kg/m^2)	1,174	21.5 ± 2.9	Birth weight (g)	1,165	3314.4 ± 428.8
Han ethnicity (%)	1,174		Birth length (cm)	1,149	49.7 ± 1.9
Yes		1,151 (98.0%)	New born sex	1,172	
No		23 (2.0%)	Female		561 (47.9%)
Marital status (%)	1,174		Male		611 (52.1%)
Single		16 (1.4%)	Birth outcomes		
Married		1,158 (98.6%)	Preterm birth (PTB)	1,174	
Primiparity (%)	1,174		Yes		33 (2.8%)
Yes		914 (77.9%)	No		1,141 (97.2%)
No		260 (22.1%)	Low birth weight (LBW)	1,174	
History of miscarriage or abortion (%)	1,174		Yes		30 (2.6%)
Yes		553 (47.1%)	No		1,141 (97.2%)
No		621 (52.9%)	Large for gestational age (LGA)	1,174	
Smoking/drinking during pregnancy (%)	1,174		Yes		108 (9.2%)
Yes		5 (0.4%)	No		1,066 (90.8%)
No		1,169 (99.6%)	Small for gestational age (SGA)	1,174	
Education level	946		Yes		84 (7.2%)
Low: High school or below		306 (32.3%)	No		1,090 (92.8%)
High: College/uni or above		640 (67.6%)	BSID test	946	
Job	946		MDI score		94.7 ± 17.7
Full-time		762 (80.5%)	PDI score		87.4 ± 14.9
Housewife		82 (8.7%)	Mental development	946	
Others		102 (10.8%)	Delay (MDI < 85)		276 (27.1%)
Household income (Monthly)	946		Normal (MDI ≥ 85)		741 (72.9%)
<2000 RMB		186 (19.7%)	Psychomotor Development	946	
2000-4000 RMB		329 (34.8%)	Delay (PDI < 85)		431 (42.4%)
4000-7000 RMB		292 (30.9%)	Normal (PDI ≥ 85)		586 (57.6%)
7000-10000 RMB		139 (14.7%)	Season of birth	1,174	
			Spring (Mar-May)		411 (35.01%)
			Summer (Jun-Aug)		263 (22.40%)
			Autumn (Sep-Nov)		198 (16.87%)
			Winter (Dec-Feb)		302 (25.72%)

For peer review only

For peer review only - http://bmjopen.bmj.com/site/about/guidelines.xhtml

Association with birth outcomes

In the unadjusted models (**eTable 5 in the Supplement**), higher exposure concentrations of $PM_{2.5}$ in T3 were significantly associated with lower birth length (β: -0.32, 95% CI: -0.51, -0.13; per IQR increase). We also observed increased NO_2 in T3 were significantly associated with lower birth length (β: -0.16, 95% CI: -0.32, -0.01; per IQR). A risk between SGA and increases in NO_2 (per IQR) was found in T2 (OR: 1.46, 95% CI: 1.10, 1.93), T3 (OR: 1.58, 95% CI: 1.14, 2.18) and in the whole pregnancy period (OR: 1.44, 95% CI: 1.13, 1.85). We observed no evidence of associations of NO_2 with overall birth weight, birth length and other adverse birth outcomes (e.g., PTB, LBW, and LGA).

In the adjusted models (**Table 2**), we found increased effect size for NO_2 and SGA in T2 (OR: 1.57, 95% CI: 1.06, 2.32), and slightly reduced effects size for NO_2 and SGA in the whole pregnancy period (OR: 1.33, 95% CI: 1.01, 1.75) compared with the unadjusted model. We observed no evidence of associations with birth length in the adjusted models. After co-adjustment for $PM_{2.5}$ (**see eTable 6 in the Supplement**), the association of NO_2 with SGA was also found in T1 (OR: 1.70, 95% CI: 1.07, 2.69), T3 (OR: 1.77, 95% CI: 1.08, 2.91) and in the whole pregnancy period (OR: 1.60, 95% CI: 1.15, 2.23).

For peer review only - http://bmjopen.bmj.com/site/about/guidelines.xhtml

Table 2 Associations between $PM_{2.5}$ and NO_2 exposure in different pregnancy periods and adverse birth outcomes (adjusted models)

Per IQR increase in		Mean difference		Odd ratios			
		Birth weight, grams (95% CI) (N=941)	Birth length, cm (95% CI) (N=927)	PTB (case: 33) (95% CI) (N=945)	LBW (case: 30) (95% CI) (N=945)	LGA (case: 108) (95% CI) (N=945)	SGA (case: 84) (95% CI) (N=945)
Estimated exposure to $PM_{2.5}$	90 days prior to conception	59.73 (-16.52, 135.98)	0.15 (-0.176, 0.48)	0.24 (0.06, 1.00)	0.49 (0.18, 1.29)	1.40 (0.72, 2.71)	1.66 (0.75, 3.68)
	First trimester	6.21 (-73.79, 86.20)	0.04 (-0.308, 0.388)	0.88 (0.28, 2.80)	0.76 (0.21, 2.81)	0.86 (0.45, 1.67)	1.33 (0.58, 3.04)
	Second trimester	-37.64 (-107.73, 32.44)	0.02 (-0.283, 0.326)	1.62 (0.53, 4.96)	1.34 (0.38, 4.68)	1.00 (0.55, 1.83)	0.94 (0.50, 1.76)
	Third trimester	4.20 (-73.17, 81.57)	-0.17 (-0.509, 0.162)	0.92 (0.29, 2.90)	0.92 (0.30, 2.85)	1.29 (0.65, 2.53)	0.83 (0.42, 1.66)
	Total pregnancy	8.01 (-41.10, 57.11)	0.02 (-0.198, 0.230)	0.77 (0.38, 1.54)	0.62 (0.31, 1.25)	1.15 (0.75, 1.77)	0.84 (0.52, 1.35)
Estimated exposure to NO_2	90 days prior to conception	-1.03 (-41.88, 39.81)	-0.04 (-0.215, 0.139)	0.84 (0.45, 1.57)	1.04 (0.54, 1.98)	1.31 (0.91, 1.88)	1.45 (0.99, 2.12)
	First trimester	-9.78 (-50.84, 31.28)	0.04 (-0.133, 0.223)	0.90 (0.49, 1.65)	1.03 (0.56, 1.91)	1.21 (0.85, 1.72)	**1.57 (1.06, 2.32)**
	Second trimester	-20.82 (-59.11, 17.47)	-0.06 (-0.222, 0.112)	1.31 (0.73, 2.34)	1.34 (0.75, 2.40)	1.21 (0.86, 1.70)	1.36 (0.95, 1.95)
	Third trimester	-9.50 (-56.00, 36.99)	-0.01 (-0.213, 0.191)	0.79 (0.40, 1.59)	0.95 (0.47, 1.94)	1.42 (0.94, 2.13)	1.51 (0.97, 2.36)
	Total pregnancy	-8.45 (-37.73, 20.83)	0.00 (-0.125, 0.130)	0.97 (0.62, 1.51)	1.04 (0.66, 1.64)	1.20 (0.93, 1.56)	**1.33 (1.01, 1.75)**

All significant findings in the table are bold.
Models adjusted for maternal age at enrolment, infant's sex, maternal BMI at 11–14 weeks' gestation, primiparity, monthly household income level, and season of births.

For peer review only

For peer review only - http://bmjopen.bmj.com/site/about/guidelines.xhtml

Association with infant neurodevelopment outcomes

In unadjusted models, $PM_{2.5}$ exposure in the 90 days prior to conception was associated with lower MDI and PDI scores in offspring (β: -3.54, 95% CI: -5.37, -1.71; β: -3.42, 95% CI: -4.96, -1.89) (**Table 3**). We also observed an unexpected positive association between $PM_{2.5}$ exposures in second trimester with MDI (β: 4.21, 95% CI: 2.43, 6.00) and PDI (β: 2.63, 95% CI: 1.12, 4.14). Exposure to NO_2 was associated with lower MDI (-1.90, 95% CI: -3.36, -0.44) and PDI in the 90 days prior to conception (-2.86, 95% CI: -4.08, -1.65). NO_2 exposure was also associated with lower PDI scores in T3 (-1.97, 95% CI: -3.29, -0.65) and in the whole pregnancy periods (-1.08, 95% CI: -2.11, -0.05). We did not observe any association between NO_2 and MDI in any pregnancy periods.

In the adjusted models (Table 3), we found $PM_{2.5}$ exposure in the 90 days prior to conception was associated with lower PDI scores (β: -6.15, 95% CI: -8.84, -3.46). Similarly, there was also a significant association of increased NO_2 exposure and lower PDI score in the 90 days prior to conception (β: -2.83, 95% CI: -4.27, -1.39), T1 (β: -1.91, 95% CI: -3.37, -0.46), T3 (β: -1.92, 95% CI: -3.57, -0.26) and whole pregnancy period (β: -1.15, 95% CI: -2.19, -0.11). The positive association between $PM_{2.5}$ exposures in second trimester with PDI (β: 3.76, 95% CI: 1.27, 6.24) remained. We did not observe any association with MDI in any pregnancy periods.

In the co-exposure models (**Table 3**), $PM_{2.5}$ exposure in the 90 days prior to conception was associated with lower PDI scores (β: -4.74, 95% CI: -7.73, -1.75). We also observed a positive association between $PM_{2.5}$ exposures in second trimester with PDI (β: 5.51, 95% CI:2.73, 8.28). Exposure to NO_2 was significantly associated with lower PDI in 90D (β: -1.72, 95% CI: -3.31, -0.12), T1 (β: -1.80, 95% CI: -3.46, -0.15), T2 (β: -2.11, 95% CI: -3.63, -0.60), T3 (β: -1.92, 95% CI: -3.76, -0.09) and whole pregnancy period (β: -1.68, 95% CI: -2.89, -0.46).

For peer review only - http://bmjopen.bmj.com/site/about/guidelines.xhtml

Table 3 Associations between $PM_{2.5}$ and NO_2 exposure in different pregnancy periods and continuous BSID scores

Per IQR increase in		Crude models		Adjusted models*		Co-exposure models**	
		MDI (95% CI) (N=946)	PDI 95% CI (N=946)	MDI (95% CI) (N=945)	PDI 95% CI (N=945)	MDI (95% CI) (N=945)	PDI 95% CI (N=945)
Estimated exposure to $PM_{2.5}$	90 days prior to conception	**-3.54 (-5.37, -1.71)**	**-3.42 (-4.96, -1.89)**	-1.98 (-5.19, 1.23)	**-6.15 (-8.84, -3.46)**	-1.73 (-5.30, 1.85)	**-4.74 (-7.73, -1.75)**
	First trimester	-1.07 (-2.93, 0.79)	0.04 (-1.52, 1.61)	-1.66 (-5.02, 1.70)	-2.11 (-4.95, 0.73)	-2.84 (-6.65, 0.97)	-0.45 (-3.67, 2.76)
	Second trimester	**4.21 (2.43, 6.00)**	**2.63 (1.12, 4.14)**	3.79 (0.85, 6.73)	**3.76 (1.27, 6.24)**	4.19 (0.89, 7.49)	**5.51 (2.73, 8.28)**
	Third trimester	-1.43 (-3.41, 0.55)	**-1.76 (-3.42, -0.10)**	-2.73 (-5.99, 0.53)	-1.37 (-4.12, 1.39)	-3.84 (-7.46, -0.22)	0.04 (-3.02, 3.09)
	Total pregnancy	**1.64 (0.06, 3.21)**	0.5 (-0.82, 1.83)	-0.27 (-2.34, 1.80)	0.23 (-1.52, 1.98)	-0.85 (-3.28, 1.57)	1.69 (-0.35, 3.73)
Estimated exposure to NO_2	90 days prior to conception	**-1.90 (-3.36, -0.44)**	**-2.86 (-4.08, -1.65)**	-0.72 (-2.43, 1.00)	**-2.83 (-4.27, -1.39)**	-0.31 (-2.22, 1.60)	**-1.72 (-3.31, -0.12)**
	First trimester	-0.08 (-1.57, 1.42)	-1.17 (-2.43, 0.08)	0.59 (-1.14, 2.32)	**-1.91 (-3.37, -0.46)**	1.28 (-0.68, 3.24)	**-1.80 (-3.46, -0.15)**
	Second trimester	**1.81 (0.41, 3.22)**	0.00 (-1.18, 1.18)	0.56 (-1.05, 2.17)	-0.75 (-2.11, 0.61)	-0.48 (-2.28, 1.33)	**-2.11 (-3.63, -0.60)**
	Third trimester	0.04 (-1.54, 1.62)	**-1.97 (-3.29, -0.65)**	0.51 (-1.45, 2.47)	**-1.92 (-3.57, -0.26)**	1.52 (-0.66, 3.69)	**-1.92 (-3.76, -0.09)**
	Total pregnancy	0.67 (-0.56, 1.89)	**-1.08 (-2.11, -0.05)**	0.41 (-0.83, 1.64)	**-1.15 (-2.19, -0.11)**	0.67 (-0.77, 2.12)	**-1.68 (-2.89, -0.46)**

All significant findings in the table are bold.

*Models adjusted for maternal age at enrolment, infant's sex, maternal BMI at 11–14 weeks' gestation, primiparity, monthly household income level, and season of births.

**Models adjusted for maternal age at enrolment, infant's sex, maternal BMI at 11–14 weeks' gestation, primiparity, monthly household income level, and season of births, and adjusted for the other air pollutant.

For peer review only

In the adjusted model, the risk of PDD was found to increase by 112% and 42% with each per-IQR increase in $PM_{2.5}$ (OR: 2.12, 95% CI: 1.45, 3.11) and NO_2 (OR: 1.42, 95% CI: 1.16, 1.75) in the 90 days prior to conception (**Table 4**). There was also a significant association between increased NO_2 exposure and the risk of PDD in T1 (OR: 1.29, 95% CI: 1.05, 1.58), T3 (OR: 1.27, 95% CI: 1.01, 1.60), and the whole pregnancy period (OR: 1.17, 95% CI: 1.02, 1.36). We did not observe any association with MDD in any pregnancy periods.

For peer review only

For peer review only - http://bmjopen.bmj.com/site/about/guidelines.xhtml

Table 4 Associations between $PM_{2.5}$ and NO_2 exposure in different pregnancy periods and mental and psychomotor developmental delay

Per IQR increase in		Crude models		Adjusted models*		Co-exposure models**	
		MDD (95% CI) (N=946)	PDD (95% CI) (N=946)	MDD (95% CI) (N=945)	PDD (95% CI) (N=945)	MDD (95% CI) (N=945)	PDD (95% CI) (N=945)
Estimated exposure to $PM_{2.5}$	90 days prior to conception	**1.45 (1.16, 1.83)**	**1.49 (1.20, 1.83)**	0.95 (0.64, 1.42)	**2.12 (1.45, 3.11)**	0.97 (0.63, 1.51)	**1.78 (1.17, 2.71)**
	First trimester	1.05 (0.83, 1.33)	1.04 (0.84, 1.28)	1.14 (0.73, 1.79)	1.42 (0.96, 2.11)	1.35 (0.80, 2.25)	1.16 (0.74, 1.82)
	Second trimester	**0.63 (0.49, 0.80)**	**0.77 (0.63, 0.95)**	0.81 (0.54, 1.22)	0.72 (0.51, 1.02)	0.83 (0.52, 1.31)	**0.57 (0.38, 0.85)**
	Third trimester	1.23 (0.96, 1.58)	1.19 (0.95, 1.49)	1.25 (0.82, 1.90)	1.17 (0.80, 1.70)	1.39 (0.87, 2.23)	0.98 (0.64, 1.49)
	Total pregnancy	0.84 (0.69, 1.03)	0.98 (0.82, 1.18)	1.07 (0.82, 1.39)	1.07 (0.84, 1.35)	1.17 (0.86, 1.59)	0.90 (0.68, 1.20)
Estimated exposure to NO_2	90 days prior to conception	1.20 (0.99, 1.45)	**1.41 (1.19, 1.67)**	0.97 (0.77, 1.21)	**1.42 (1.16, 1.75)**	0.97 (0.76, 1.25)	1.24 (0.99, 1.56)
	First trimester	0.97 (0.80, 1.17)	1.18 (0.99, 1.40)	0.91 (0.72, 1.13)	**1.29 (1.05, 1.58)**	0.84 (0.65, 1.09)	1.24 (0.99, 1.57)
	Second trimester	**0.79 (0.66, 0.95)**	1.04 (0.88, 1.22)	0.94 (0.76, 1.15)	1.14 (0.95, 1.38)	0.98 (0.77, 1.24)	**1.31 (1.06, 1.63)**
	Third trimester	1.04 (0.85, 1.28)	**1.25 (1.04, 1.50)**	0.94 (0.73, 1.21)	**1.27 (1.01, 1.60)**	0.86 (0.65, 1.14)	1.28 (0.99, 1.65)
	Total pregnancy	0.92 (0.79, 1.07)	**1.16 (1.01, 1.33)**	0.94 (0.80, 1.11)	**1.17 (1.02, 1.36)**	0.9 (0.75, 1.08)	**1.21 (1.02, 1.43)**

All significant findings in the table are bold.

*Models adjusted for maternal age at enrolment, infant's sex, maternal BMI at 11–14 weeks' gestation, primiparity, monthly household income level, and season of births.

**Models adjusted for maternal age at enrolment, infant's sex, maternal BMI at 11–14 weeks' gestation, primiparity, monthly household income level, and season of births, and adjusted for the other air pollutant.

For peer review only

Discussion

We analyzed associations between modelled $PM_{2.5}$ and NO_2 pre- and during pregnancy with birth and neurodevelopment outcomes in singleton children born in a south-western metropolis of China in 2015-16. We found the likelihood of SGA increased by 33% per IQR higher exposure to NO_2 in the whole pregnancy periods after adjusted for maternal age at enrolment, infant's sex, maternal BMI at 11–14 weeks' gestation, primiparity, monthly household income level, and season of births and $PM_{2.5}$. For childhood cognitive development, increased exposure to $PM_{2.5}$ and NO_2 in the 90 days prior to conception were both associated with lower PDI scores, with the effect size per IQR being higher for $PM_{2.5}$ than for NO_2. Increased NO_2 exposure was associated with an increased risk of PDD during different trimesters of pregnancy.

Many studies from other geographic areas, including Europe (33-35), the United States (22, 26), and Asia (23, 36-38) have found that prenatal air pollution exposure has a negative impact on a variety of neurodevelopmental outcomes. Our finding of a negative association between prenatal NO_2 air pollution exposure and infant neurocognitive development is consistent with these reports. A recent Chinese birth cohort study of 15,778 child-mother pairs in Foshan reported that maternal NO_2 exposure during pregnancy was associated with an increased risk of suspected developmental delay (OR: 1.06, 95% CI: 0.94, 1.19) measured by a five-domain scale and developmental quotient (DQ) (23). A birth cohort study of 520 mother-child pairs in South Korea reported that maternal NO_2 exposure during pregnancy was associated with impairment of psychomotor development ($\beta = -1.30$, $p = 0.05$) but – as in the present study - not with cognitive function ($\beta = -0.84$, $p = 0.20$) (36). However, results from previous research varied by air pollutants. For example, a Chinese study of 1193 mother-newborn pairs in Changsha found significant associations between $PM_{2.5}$ exposure in trimester two and lower neurobehavioral developmental scores, while other air pollutants such as PM_{10}, carbon monoxide (CO), and Sulfur dioxide (SO_2) had null or even reverse associations. In this study, we observed that the negative effect of NO_2 exposure during pregnancy on PDI is significant at 5% level; this negative effect of NO_2 still remained after adjustment for $PM_{2.5}$. This heterogeneity may relate to the temporality of exposure assessment, types of outcome assessment instruments or evaluators, and the levels of air pollution. In addition, air pollution mixtures may have differed among the study regions, thus there are several potential explanations for the heterogeneity of the findings. We also observed negative correlations between certain exposures, indicating the need to consider potential collinearity in our two-pollutant models. In Chongqing, a major industrial city in southwest China, air pollution may

For peer review only - http://bmjopen.bmj.com/site/about/guidelines.xhtml

come from industrial and traffic emissions, construction activities, and dust, and negative correlations may occur if different sources contribute disproportionately to each pollutant. Their correlations may also be affected by seasonal changes and variations in weather patterns. Future research should also explore the impact of source-specific air pollution on children's cognitive health.

To date, most studies on prenatal air pollution exposure and child neurodevelopment have been conducted in developed countries with relatively low levels of air pollution. In this study, the level of air pollution was higher (median $PM_{2.5}$: 57.31 μg/m^3, IQR: 5.76; median NO_2: 50.46 μg/m^3, IQR: 5.51) compared to studies in developed countries such as Europe and the United States. In a multi-centre European cohort, the mean $PM_{2.5}$ and NO_2 exposure concentration during pregnancy were 13.4 μg/m^3 and 11.5 μg/m^3 (33). Researchers found that the psychomotor development score significantly decreased by 0.68 points (95% CI: -1.25, -0.11) for every 10 μg/m^3 increase in NO_2, and there was also a non-significant decrease of 1.64 points (95% CI: -3.47, 0.18) for every 5 μg/m^3 increase in $PM_{2.5}$ during pregnancy (33). Factors such as the types of pollutants and concentrations may differ between China and other regions with a lower air pollution level, leading to variations in the observed effects.

Contrary to expectations**,** we found significant positive associations between prenatal exposure to $PM_{2.5}$ air pollution in the second trimester and PDI. However, no association was observed between $PM_{2.5}$ exposures in the second trimester and the risk of PDD. Given the existing literature and the conflicted observation here, we believe that this is likely to be spurious/sample specific. Some plausible explanations include the uneven distribution of PDI scores, the potentially inappropriate selection of the cut-off value of 85 (which may not effectively discriminate between groups), or the possibility that the observed outcome occurred by chance. Several epidemiological studies have reported associations between prenatal exposure to high levels of $PM_{2.5}$ and lower neurodevelopment in children ranging in age from 6 months to 6 years (12, 34, 39-41). In agreement with our findings, a multi-centre cohort study from six European countries investigated the effects of prenatal exposure to multiple air pollutants including $PM_{2.5}$, PM_{10}, coarse particles, NO_2 and nitrogen oxides (NOx) among 9482 children between 1 and 6 years; the authors found nonsignificant positive associations between prenatal $PM_{2.5}$ exposure and normal neurodevelopment (β: 1.64, 95% CI: −3.47, 0.18; per 5 μg/m^3 increase in $PM_{2.5}$) (33). Similarly, another study examining the effects of multiple pollutant exposures on early childhood cognition at 40 days of age in a highly exposed area of Spain also found PM_{10}, PM_{coarse}, $PM_{2.5absorbance}$, NO_2, NO_x, and Ozone (O_3) were linked to lower

For peer review only - http://bmjopen.bmj.com/site/about/guidelines.xhtml

For peer review only

motor function in children, except for $PM_{2.5}$ (42). The inconsistent findings could be because of heterogeneity between studies in terms of exposure (e.g., exposure assessment methods used, $PM_{2.5}$ exposure levels, or composition of $PM_{2.5}$).

Aside from the conflicting findings regarding prenatal $PM_{2.5}$ exposure and neurodevelopmental outcomes, results regarding the most potential sensitive time windows before and during pregnancy are also inconclusive. Some studies suggested that early-to-mid pregnancy might be a potential sensitive period (22, 23), while other studies found stronger associations for middle-to- late pregnancy, thus results are equivocal (20, 25, 26).

The potential biological mechanisms by which air pollution could affect neurodevelopment are not yet clearly understood. There is evidence suggesting that exposure to prenatal $PM_{2.5}$ could potentially induce maternal immune activation during pregnancy (43). Higher levels of cytokines or reactive oxygen species may potentially interfere fetal neurodevelopment through three mechanisms: crossing the placental barrier into the fetal body, inducing fetal immune dysregulation, and contributing to inadequate placental perfusion that affects nutritional processes and oxygenation of maternal blood(44). More research is needed to investigate trimester effects of air pollution on neurodevelopment and provide better understanding on the underlying biological mechanisms. Our study is the first to consider an exposure window 90 days prior to conception for NO_2. A novel observation is that effects of NO_2 or $PM_{2.5}$ air pollution on child cognition can be seen at least 90 days prior to conception, representing a potentially vulnerable periods in relation to air pollution on neurodevelopment. Similar results were found in previous study recruited 1329 mother-child pairs in Wuhan, China (12). This study reported a higher level of $PM_{2.5}$ during preconception (Median: 76.1 $\mu g/m^3$) and in the first trimester (Median: 82.3 $\mu g/m^3$). This study found for each doubling of $PM_{2.5}$ exposure during preconception, children's PDI scores was reduced by 6.15 (95% CI: -8.84, -3.46) points. A potential explanation is that preconception air pollution exposures induce genetic and epigenetic alterations in sperm, that increase the risk of adverse health outcomes in offspring (45, 46). To date, all studies examined the effect of maternal preconception exposure while omitting paternal exposures (17). Future studies should consider the effect of preconception paternal exposure in relation to childhood health outcomes.

This study has several strengths. We developed an LUR model to capture spatial and temporal variations of air pollution at individual level to reduce exposure misclassification if using monitoring stations. This is an novel study to investigate both pre-conception and prenatal

For peer review only - http://bmjopen.bmj.com/site/about/guidelines.xhtml

$PM_{2.5}$ and NO_2 exposure with neurodevelopment outcomes among young infants, in the context of a relatively high air pollution urban environment. The exposure levels in our study were similar as those in comparable urban areas in Chinese cities. A study in Shanghai, China reported an average NO_2 exposure during pregnancy from 2014 to 2015, predicted by the LUR model, of 48.23 $\mu g/m^3$ (Mean $PM_{2.5}$ in our study: 50.52 $\mu g/m^3$) (47). Similarly, a study in Tianjin found the annual average $PM_{2.5}$ exposure to be 62 $\mu g/m^3$ in 2017 (Mean NO_2 in our study: 57.48 $\mu g/m^3$) (48). Wu et al. developed a LUR model for $PM_{2.5}$ in the main urban area of Chongqing (49). This model predicted an annual average $PM_{2.5}$ concentration of 40.6 $\mu g/m^3$ (49), whereas our prediction is higher at 55.9 $\mu g/m^3$ (19). This difference can be attributed to the temporal variations. Wu et al. used monitoring data from 2013, while we utilized data from 2015. It could be considered that our GAM model, with its temporal component, could explain temporal variations and is more suitable for pregnancy-specific exposure estimates.

A major limitation of this study was that our sample size was relatively small, limiting the statistical power to assess several outcomes, although the higher exposures in Chongqing than in some other studies may increase probability of detecting effects. In terms of limitations, due to a lack of information on participant time-activity patterns, exposure estimates in this study refer only to ambient concentrations at home addresses, and no other activity spaces (e.g., indoor, workplace, commuting) were considered. We may have thus underestimated total air pollution exposure. Second, we defined exposure windows for clinically-defined trimesters; sensitive periods may be shorter or longer than 3 months, or they may exist in the overlap of multiple trimesters. However, we were unable to investigate the sensitive time windows using established methods such as distributed lag non-linear models due to the lack of highly time-resolved air pollution estimates. Third, the performance of the NO_2 spatiotemporal model was moderate (COR-R^2: 0.39), which may introduce exposure misclassification and therefore bias in the coefficients. Finally, we were unable to include some other air pollutants such as polycyclic aromatic hydrocarbons (PAH), black carbon (BC) and Ozone, which have bene found particular harmful to neurodevelopment in children (50). Although we have accounted for most of the important confounders in this study, unfortunately, we did not collect information on the feeding patterns of infants. This may undermine the validity and reliability of our findings.

Conclusion

For peer review only

For peer review only - http://bmjopen.bmj.com/site/about/guidelines.xhtml

This study provides evidence for an association between NO_2 exposure prior to- and during pregnancy with birth and neurodevelopmental outcomes in a birth cohort in Chongqing, China. Exposure to NO_2 and $PM_{2.5}$ exposure before pregnancy was associated with a lower psychomotor development score. Increased NO_2 exposure was linked to a risk of psychomotor development delay during various pregnancy trimesters.

For peer review only

For peer review only - http://bmjopen.bmj.com/site/about/guidelines.xhtml

212 **List of abbreviations**

ADHD	Attention deficit hyperactivity disorder
ASD	Autism spectrum disorder
BMI	Body mass index
BSID	Bayley Scales of Infant Development
CBSID	Chinese version of Bayley Scales of Infant Development
CI	Confidence interval
CLIMB	Complex Lipids in Mothers and Babies
CO	Carbon monoxide
COR	Correlation
DOHaD	Developmental origins of health and disease
DQ	Developmental quotient
GIS	Geographic information systems
IQR	Interquartile range
LBW	Low birth weight
LGA	Large for gestational age
LUR	Land-use Regression
MDD	Mental Developmental Delay
MDI	Mental Development Index
NO_x	Nitrogen oxides
NO_2	Nitrogen dioxide
OR	Odd ratio
O_3	Ozone

For peer review only - http://bmjopen.bmj.com/site/about/guidelines.xhtml

PDD	Psychomotor Developmental Delay
PDI	Psychomotor Development Index
PM	Particulate matter
$PM_{2.5}$	Particulate matter with diameter ≤2.5μm
PTB	Preterm birth
SGA	Small for gestational age
SO_2	Sulfur dioxide
TLBW	Term low birth weight
T1	First trimester
T2	Second trimester
T3	Third trimester
WP	Whole pregnancy period
90D	90 days prior to pregnancy

213

For peer review only

For peer review only - http://bmjopen.bmj.com/site/about/guidelines.xhtml

Declarations

Ethics approval and consent to participate

Ethical approval for this study was granted by the Ethics Committee of Chongqing Medical University (#2014034). The participants provided their written informed consent to participate in this study. Written informed consent was obtained from the individual(s) for the publication of any potentially identifiable images or data included in this article.

Data availability statement

The data that support the findings of this study are available from Chongqing Medical University but restrictions apply to the availability of these data, which were used under license for the current study, and so are not publicly available. Data are however available from the authors upon reasonable request and with permission of Chongqing Medical University.

Competing interests

The authors declare that they have no conflicts of interests.

Funding

This work was supported by the National Natural Science Foundation of China (No. 81971406, 82271715), The 111 Project (Yuwaizhuan (2016)32), Chongqing Science & Technology Bureau (CSTB2022NSCQ-MSX1680), Youth Innovation Team Development Support Program of Chongqing Medical University (W0083), and Smart Medicine Research Project of Chongqing Medical University (No. ZHYX202103), Zunyi science and technology plan project (Zunshikehe HZ (2022)153). The research was supported by National Institute for Health Research (NIHR) Health Protection Research Unit in Environmental Exposures and Health, a partnership between UK Health Security Agency, the Health and Safety Executive and the University of Leicester and by the NIHR Leicester Biomedical Research Centre (BRC). The views expressed are those of the author(s) and not necessarily those of the NIHR, the Department of Health and Social Care or UK Health Security Agency.

Contributors

Y. X., T.L.H., H.Z. and P.B. conceived and designed research; T.Z., Y.X. and H.Z. recruited the patients and collected the samples; T.K., A.H., and J.G. constructed the air pollution model; Y.C. analyzed, interpreted the data and prepared the figures; Y.C and T.K were major

For peer review only

For peer review only - http://bmjopen.bmj.com/site/about/guidelines.xhtml

contributors in writing the manuscript text; YSC, JC, TLH, YX, ALH, and PNB substantively revised the manuscript; All authors read and approved the final manuscript.

Acknowledgements

The authors would like to acknowledge the clinical research staff who recruited subjects and facilitated sample collection, the women and their families who participated in the CLIMB study, and Jamie de Seymour, the leading author for diet pattern analysis in the CLIMB cohort, for her help and advice during the analysis process.

For peer review only

For peer review only - http://bmjopen.bmj.com/site/about/guidelines.xhtml

Reference

1. Llop S, Ballester F, Estarlich M, Esplugues A, Rebagliato M, Iniguez C. Preterm birth and exposure to air pollutants during pregnancy. Environmental Research. 2010;110(8):778-85.
2. Ambient air pollution and low birthweight: a European cohort study (ESCAPE). The Lancet Respiratory Medicine. 2013;1(9):695-704.
3. Stieb DM, Chen L, Hystad P, Beckerman BS, Jerrett M, Tjepkema M, et al. A national study of the association between traffic-related air pollution and adverse pregnancy outcomes in Canada, 1999–2008. Environmental Research. 2016.
4. Barker DJ. The origins of the developmental origins theory. Journal of internal medicine. 2007;261(5):412-7.
5. Volk HE, Perera F, Braun JM, Kingsley SL, Gray K, Buckley J, et al. Prenatal air pollution exposure and neurodevelopment: A review and blueprint for a harmonized approach within ECHO. Environmental research. 2021;196:110320.
6. Feng S, Dan G, Liao F, Zhou F, Wang X. The health effects of ambient PM2.5 and potential mechanisms. Ecotoxicology and Environmental Safety. 2016;128:67-74.
7. Massa NR, Guangyun M, Xingyou Z, Xiumei H, Zhu C, Sampankanpanich SC, et al. Intrauterine Inflammation and Maternal Exposure to Ambient PM2.5 during Preconception and Specific Periods of Pregnancy: The Boston Birth Cohort. Environ Health Perspect. 2016;124(10):1608-15.
8. Vecoli C, Montano L, Andreassi MG. Environmental pollutants: genetic damage and epigenetic changes in male germ cells. Environmental Science and Pollution Research. 2016;23:23339-48.
9. Marcho C, Oluwayiose OA, Pilsner JR. The preconception environment and sperm epigenetics. Andrology. 2020;8(4):924-42.
10. Udagawa O, Furuyama A, Imai K, Fujitani Y, Hirano S. Effects of diesel exhaust-derived secondary organic aerosol (SOA) on oocytes: Potential risks to meiotic maturation. Reproductive Toxicology. 2018;75:56-64.
11. Zhang J-Y, Wu Q-J, Huang Y-H, Li J, Liu S, Chen Y-L, et al. Association between maternal exposure to ambient PM10 and neural tube defects: a case-control study in Liaoning Province, China. International Journal of Hygiene and Environmental Health. 2020;225:113453.

For peer review only - http://bmjopen.bmj.com/site/about/guidelines.xhtml

12. Li J, Liao J, Hu C, Bao S, Mahai G, Cao Z, et al. Preconceptional and the first trimester exposure to PM2. 5 and offspring neurodevelopment at 24 months of age: Examining mediation by maternal thyroid hormones in a birth cohort study. Environmental Pollution. 2021;284:117133.

13. Jo H, Eckel SP, Chen J-C, Cockburn M, Martinez MP, Chow T, et al. Gestational diabetes mellitus, prenatal air pollution exposure, and autism spectrum disorder. Environment international. 2019;133:105110.

14. Raz R, Roberts AL, Lyall K, Hart JE, Just AC, Laden F, et al. Autism spectrum disorder and particulate matter air pollution before, during, and after pregnancy: a nested case–control analysis within the Nurses' Health Study II cohort. Environmental health perspectives. 2015;123(3):264-70.

15. Grineski S, Alexander C, Renteria R, Collins TW, Bilder D, VanDerslice J, et al. Trimester-specific ambient PM2.5 exposures and risk of intellectual disability in Utah. Environmental Research. 2023;218:115009.

16. Shang L, Yang L, Yang W, Huang L, Qi C, Yang Z, et al. Effects of prenatal exposure to NO(2) on children's neurodevelopment: a systematic review and meta-analysis. Environ Sci Pollut Res Int. 2020;27(20):24786-98.

17. Blanc N, Liao J, Gilliland F, Zhang JJ, Berhane K, Huang G, et al. A systematic review of evidence for maternal preconception exposure to outdoor air pollution on Children's health. Environmental Pollution. 2022:120850.

18. Huang S, Mo T-T, Norris T, Sun S, Zhang T, Han T-L, et al. The CLIMB (Complex Lipids In Mothers and Babies) study: protocol for a multicentre, three-group, parallel randomised controlled trial to investigate the effect of supplementation of complex lipids in pregnancy, on maternal ganglioside status and subsequent cognitive outcomes in the offspring. BMJ open. 2017;7(10):e016637.

19. Harper A, Baker PN, Xia Y, Kuang T, Zhang H, Chen Y, et al. Development of spatiotemporal land use regression models for PM2. 5 and NO2 in Chongqing, China, and exposure assessment for the CLIMB study. Atmospheric Pollution Research. 2021;12(7):101096.

20. Chen B, Huang S, He J, He Q, Chen S, Liu X, et al. Sex-specific influence of prenatal air pollutant exposure on neonatal neurobehavioral development and the sensitive window. Chemosphere. 2020;254:126824.

For peer review only

For peer review only - http://bmjopen.bmj.com/site/about/guidelines.xhtml

21. Volk HE, Perera F, Braun JM, Kingsley SL, Gray K, Buckley J, et al. Prenatal air pollution exposure and neurodevelopment: A review and blueprint for a harmonized approach within ECHO. Environ Res. 2021;196:110320.

22. Ha S, Yeung E, Bell E, Insaf T, Ghassabian A, Bell G, et al. Prenatal and early life exposures to ambient air pollution and development. Environmental research. 2019;174:170-5.

23. Su X, Zhang S, Lin Q, Wu Y, Yang Y, Yu H, et al. Prenatal exposure to air pollution and neurodevelopmental delay in children: A birth cohort study in Foshan, China. Science of The Total Environment. 2022;816:151658.

24. Bennet L, Walker DW, Horne RS. Waking up too early–the consequences of preterm birth on sleep development. The Journal of physiology. 2018;596(23):5687-708.

25. Wang P, Zhao Y, Li J, Zhou Y, Luo R, Meng X, et al. Prenatal exposure to ambient fine particulate matter and early childhood neurodevelopment: A population-based birth cohort study. Science of The Total Environment. 2021;785:147334.

26. Chiu Y-HM, Hsu H-HL, Coull BA, Bellinger DC, Kloog I, Schwartz J, et al. Prenatal particulate air pollution and neurodevelopment in urban children: examining sensitive windows and sex-specific associations. Environment international. 2016;87:56-65.

27. Huang S, Mo T-T, Norris T, Sun S, Zhang T, Han T-L, et al. The CLIMB (Complex Lipids In Mothers and Babies) study: protocol for a multicentre, three-group, parallel randomised controlled trial to investigate the effect of supplementation of complex lipids in pregnancy, on maternal ganglioside status and subsequent cognitive outcomes in the offspring. BMJ Open. 2017;7(10):e016637.

28. Albert BB, Derraik JG, Xia Y-Y, Norris T, Zhang T, Han T-L, et al. Supplementation with milk enriched with complex lipids during pregnancy: a double-blind randomized controlled trial. Plos one. 2021;16(2):e0244916.

29. Zhao X, Xia Y, Zhang H, Baker PN, Norris T. Birth weight charts for a Chinese population: an observational study of routine newborn weight data from Chongqing. BMC Pediatr. 2019;19(1):426.

30. Shourong Y, Xuerong L, Zhiwei Y. The revising of the bayley scales of infant development (BSID) in China. Chin J Clin Psychol. 1993;2:71-5.

31. Chen Y-T, Zhang T, Chen C, Xia Y-Y, Han T-L, Chen X-Y, et al. Associations of early pregnancy BMI with adverse pregnancy outcomes and infant neurocognitive development. Scientific Reports. 2021;11(1):1-8.

For peer review only - http://bmjopen.bmj.com/site/about/guidelines.xhtml

32. Çelik P, Sucakli IA, Yakut HI. Which Bayley-III cut-off values should be used in different developmental levels? Turkish journal of medical sciences. 2020;50(4):764-70.

33. Guxens M, Garcia-Esteban R, Giorgis-Allemand L, Forns J, Badaloni C, Ballester F, et al. Air pollution during pregnancy and childhood cognitive and psychomotor development: six European birth cohorts. Epidemiology. 2014:636-47.

34. Lertxundi A, Baccini M, Lertxundi N, Fano E, Aranbarri A, Martínez MD, et al. Exposure to fine particle matter, nitrogen dioxide and benzene during pregnancy and cognitive and psychomotor developments in children at 15 months of age. Environment international. 2015;80:33-40.

35. Porta D, Narduzzi S, Badaloni C, Bucci S, Cesaroni G, Colelli V, et al. Air pollution and cognitive development at age 7 in a prospective Italian birth cohort. Epidemiology. 2016;27(2):228-36.

36. Kim E, Park H, Hong Y-C, Ha M, Kim Y, Kim B-N, et al. Prenatal exposure to PM10 and NO2 and children's neurodevelopment from birth to 24 months of age: Mothers and Children's Environmental Health (MOCEH) study. Science of the Total Environment. 2014;481:439-45.

37. Lin C-C, Yang S-K, Lin K-C, Ho W-C, Hsieh W-S, Shu B-C, et al. Multilevel analysis of air pollution and early childhood neurobehavioral development. International journal of environmental research and public health. 2014;11(7):6827-41.

38. Yorifuji T, Kashima S, Diez MH, Kado Y, Sanada S, Doi H. Prenatal exposure to traffic-related air pollution and child behavioral development milestone delays in Japan. Epidemiology. 2016;27(1):57-65.

39. Tozzi V, Lertxundi A, Ibarluzea JM, Baccini M. Causal effects of prenatal exposure to PM2. 5 on child development and the role of unobserved confounding. International Journal of Environmental Research and Public Health. 2019;16(22):4381.

40. Hurtado-Díaz M, Riojas-Rodríguez H, Rothenberg SJ, Schnaas-Arrieta L, Kloog I, Just A, et al. Prenatal PM2. 5 exposure and neurodevelopment at 2 years of age in a birth cohort from Mexico city. International journal of hygiene and environmental health. 2021;233:113695.

41. Lertxundi A, Andiarena A, Martínez MD, Ayerdi M, Murcia M, Estarlich M, et al. Prenatal exposure to PM2.5 and NO2 and sex-dependent infant cognitive and motor development. Environmental Research. 2019;174:114-21.

For peer review only - http://bmjopen.bmj.com/site/about/guidelines.xhtml

42. Iglesias-Vázquez L, Binter A-C, Canals J, Hernández-Martínez C, Voltas N, Ambros A, et al. Maternal exposure to air pollution during pregnancy and child's cognitive, language, and motor function: ECLIPSES study. Environmental Research. 2022;212:113501.

43. Umezawa M, Onoda A, Korshunova I, Jensen AC, Koponen IK, Jensen KA, et al. Maternal inhalation of carbon black nanoparticles induces neurodevelopmental changes in mouse offspring. Particle and Fibre Toxicology. 2018;15:1-18.

44. Monk C, Lugo-Candelas C, Trumpff C. Prenatal developmental origins of future psychopathology: mechanisms and pathways. Annual review of clinical psychology. 2019;15:317-44.

45. Xu R, Zhong Y, Li R, Li Y, Zhong Z, Liu T, et al. Association between exposure to ambient air pollution and semen quality: A systematic review and meta-analysis. Science of The Total Environment. 2023;870:161892.

46. Braun JM, Messerlian C, Hauser R. Fathers matter: why it's time to consider the impact of paternal environmental exposures on children's health. Current epidemiology reports. 2017;4:46-55.

47. Ji X, Meng X, Liu C, Chen R, Ge Y, Kan L, et al. Nitrogen dioxide air pollution and preterm birth in Shanghai, China. Environmental research. 2019;169:79-85.

48. Zhang Y, Wang J, Chen L, Yang H, Zhang B, Wang Q, et al. Ambient PM2. 5 and clinically recognized early pregnancy loss: A case-control study with spatiotemporal exposure predictions. Environment International. 2019;126:422-9.

49. Wu J-S, Liao X, Peng J, Huang X-L. Simulation and influencing factors of spatial distribution of PM2. 5 concentrations in Chongqing. Huan Jing ke Xue= Huanjing Kexue. 2015;36(3):759-67.

50. Lopuszanska U, Samardakiewicz M. The relationship between air pollution and cognitive functions in children and adolescents: a systematic review. Cognitive and Behavioral Neurology. 2020;33(3):157-78.

For peer review only

For peer review only - http://bmjopen.bmj.com/site/about/guidelines.xhtml

Figures

Figure 1 Flow chart of the study population in CLIMB

Figure 2 Study area and location of monitoring sites (OpenStreetMap contributors, 2015; https://data.nextgis.com/en/region/CN-50/).

For peer review only

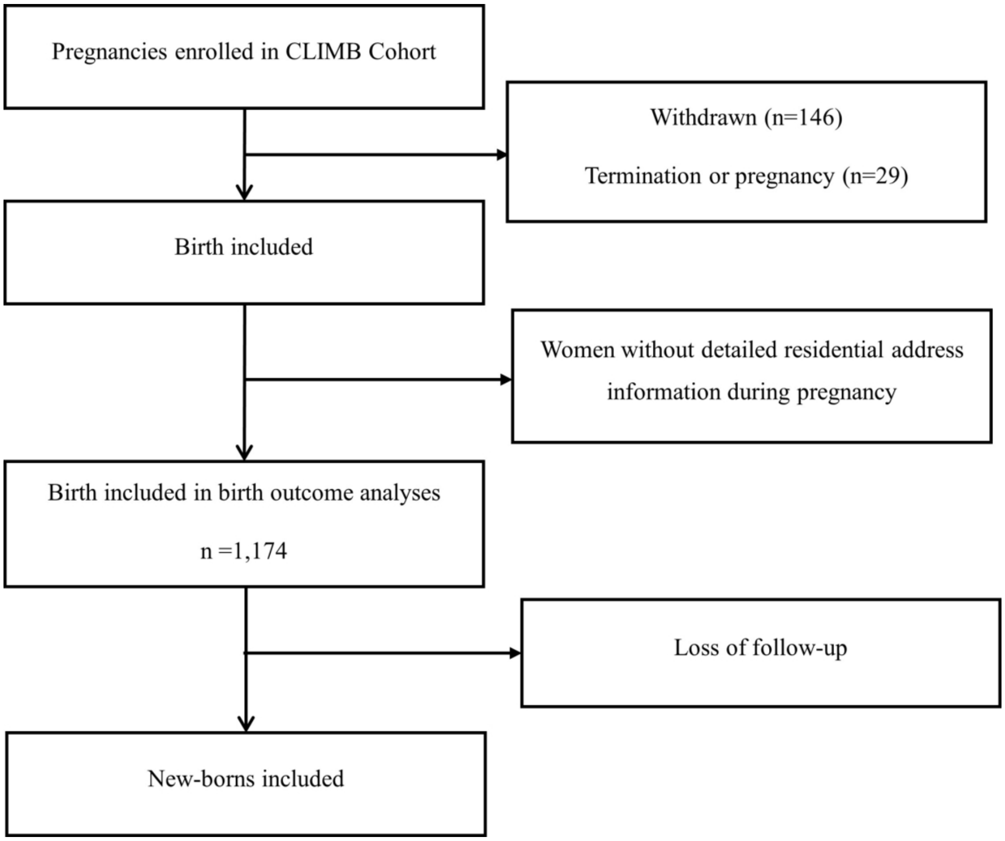

Figure 1 Flow chart of the study population in CLIMB

173x144mm (300 x 300 DPI)

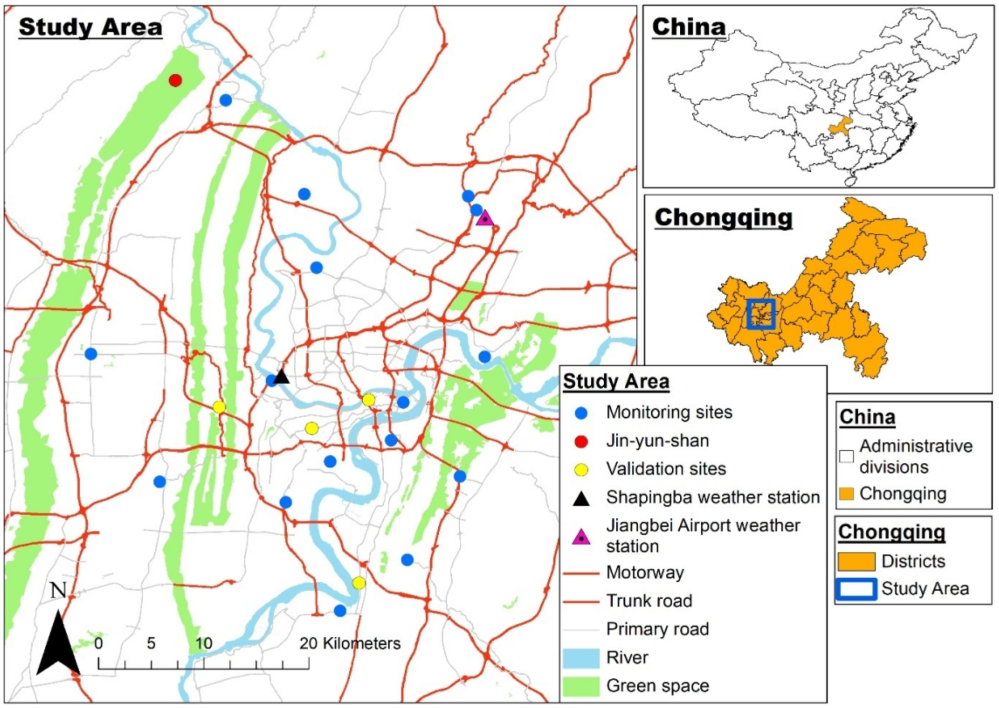

Figure 2 Study area and location of monitoring sites (OpenStreetMap contributors, 2015; https://data.nextgis.com/en/region/CN-50/).

159x112mm (220 x 220 DPI)

Associations of air pollution exposures in preconception and pregnancy with birth outcomes and infant neurocognitive development: analysis of the Complex Lipids in Mothers and Babies (CLIMB) prospective cohort in Chongqing, China

Yingxin Chen[1,2], Tao Kuang[3], Ting Zhang[4], Yutong Samuel Cai[1,2, 5], John Colombo[6], Alex Harper[7], Ting-Li Han[8], Yinyin Xia[9], John Gulliver[10], Anna L Hansell[1,2,5], Hua Zhang[8], Philip N Baker[7,8]

1: Centre for Environmental Health and Sustainability, University of Leicester, Leicester, UK

2: The National Institute of Health Research (NIHR) Health Protection Research Unit (HPRU) in Environmental Exposure and Health at the University of Leicester, Leicester, UK

3: Department of public health and management, Zunyi Medical and Pharmaceutical College, Zunyi, 563000, Guizhou, China

4. Stomatological Hospital of Chongqing Medical University, Chongqing, 401147, China

5: NIHR Leicester Biomedical Research Centre, Leicester General Hospital, Leicester, UK

6: Schiefelbusch Institute for Life Span Studies and Department of Psychology, University of Kansas, Lawrence, KS 66045, USA

7: College of Life Sciences, University of Leicester, Leicester, UK

8: Department of Obstetrics and Gynaecology, the First Affiliated Hospital of Chongqing Medical University, Chongqing, 400016, China

9: School of Public Health, Chongqing Medical University, Chongqing, 400016, China

10: Environmental and Exposure Sciences, Population Health Research Institute, St George's, University of London, London, UK

Correspondence to: Yinyin Xia. School of Public Health, Chongqing Medical University, Chongqing, 400016, China. Email: 100118@cqmu.edu.cn

For peer review only

Supplement

eTable 1 Mental Development Index (Chinese version)

智力量表

（※可偶尔观察到）

序号	月龄	条目	计分
1	0.1	对铃声反应	
2	0.1	抱起时安静	
3	0.1	对摇鼓声反应	
4	0.1	对尖声反应：（电灯开关）	
5	0.1	短暂地注视红环	
6	0.2	短暂地注视人	
7	0.4	稍长时间地注视红环	
8	0.5	眼的水平协调活动（红环）	
9	0.7	眼的水平向天活动（光）	
10	0.7	眼睛追随移动的人	
11	0.7	对说话声反应	
12	0.8	眼的垂直协调活动（光）	
13	0.9	发声一至两次	
14	1	眼的垂直协调活动（红环）	
15	1.2	眼的旋转协调活动（光）	
16	1.2	眼的旋转细条活动（光环）	
17	1.3	※自由环视周围	
18	1.5	社交笑：测试者谈话与微笑时	
19	1.6	眼转向红环	
20	1.6	眼转向光	
21	1.6	※发声至少四次	
22	1.7	期待性兴奋	
23	1.7	对面部的纸有反应	
24	1.9	能用视觉辨认母亲	
25	1.9	社交笑：测试者微笑与安静时	
26	2	※对测试者的微笑和说话有发声反应	
27	2.1	※用眼睛寻找声源（详细说明）	
28	2.2	※发出两种不同的声音	
29	2.2	对手的遮蔽眨眼	
30	2.2	对面孔的消失有反应	
31	2.4	注视方木	
32	2.6	从一物转看另一物	
33	2.6	眼睛追随铅笔	
34	2.7	对抱起有预感性的调节反应	
35	2.9	目光追随横过桌面的球	
36	2.9	头追随悬摆的环	

For peer review only

37	3.1	头追随逐渐消失的勺子	
38	3.2	操作红环	
39	3.3	简单地玩摇鼓	
40	3.4	※轻轻地抚摸桌沿	
41	3.4	※意识到陌生环境	
42	3.5	头转向铃声	
43	3.6	头转向摇鼓声	
44	3.6	※手碰手的玩耍	
45	3.6	将红环送入口中	
46	3.7	伸手够悬环	
47	3.8	看自己的手	
48	4.2	接近悬环（优势手）	
49	4.4	※发声时的姿态（描述）	
50	4.4	※主动抚摸桌沿	
51	4.4	接近镜像	
52	4.4	注意小糖丸	
53	4.6	伸手取方木	
54	4.7	喜欢嬉戏	
55	4.9	伸手时眼手协调	
56	4.9	拾起方木（优势手）	
57	5	保持两块方木	
58	5	持久地看红环	
59	5	头部跟着掉下的勺转动	
60	5	探索性地玩纸	
61	5	对镜像微笑	
62	5	坚持够东西	
63	5.1	在小床内重新找到摇鼓	
64	5.1	※辨别生人	
65	5.4	举起倒扣的茶杯	
66	5.5	※敲打玩耍	
67	5.5	探索性地玩细绳	
68	5.5	伸手取第二块方木	
69	5.6	※由一手向另一手传递物体	
70	5.8	※对产生响声感兴趣	
71	5.9	灵巧而直接地拾起方木	
72	6	※对镜像开玩笑	
73	6	用把柄举起茶杯	
74	6	寻找掉落的勺子	
75	6.1	牵拉细绳获取红环	
76	6.1	保留三块方木中的两块	
77	6.6	※发出四个不同的音节	
78	6.8	能配合玩游戏	
79	7	恰当地牵拉细绳获取红环	
80	7.1	玩摇铃，对细节感兴趣	
81	7.4	企图获得三块方木	

For peer review only - http://bmjopen.bmj.com/site/about/guidelines.xhtml

82	7.4	有目的地摇铃	
83	7.5	※选择性地倾听熟悉的词语	
84	8	※对 da-da 或类同词	
85	8.1	暴露玩具	
86	8.2	注意测试者的乱写	
87	8.3	将手指插入桩板洞中	
88	8.6	观看书中图画	
89	8.9	对他人的言语要求有反应	
90	9.1	拿起茶杯获得方木	
91	9.8	寻找盒子里面的东西	
92	10.3	遵照命令将方木放入茶杯（放入数）	
93	10.7	企图模仿乱写	
94	10.8	模仿用勺子搅拌	
95	10.9	遵照命令停止	
96	10.9	推动小汽车	
97	11	模仿地拍打哨娃	
98	11.1	※重复引入发笑的把戏	
99	11.2	解开裹着的方木	
100	11.2	将三块方木放入杯中	
101	11.4	※快速而不清的表达	
102	11.4	揭开兰盒子的盖	
103	11.5	翻开书页	
104	11.5	摇晃悬环的	
105	11.8	将骰子放入盒中（6 个）	
106	12	恰当地握持画笔	
107	12.2	模仿说单词（记录用过的词）	
108	12.4	重复地插一根桩钉	
109	12.5	用手势表达想要的东西	
110	12.9	自动乱写	
111	12.9	能说两个词	
112	13	搭两层塔	
113	13.1	出示鞋子或其他衣服或自己的玩具	
114	13.2	从瓶中移出小糖丸	
115	13.3	掺九块方木放入杯中	
116	14.3	※盖上圆盒	
117	14.4	兰色模板：放置一个圆形模块	
118	14.8	用棍子够取玩具	
119	15.4	搭三层塔	
120	15.7	在 70 秒钟内插完桩钉	
121	16.1	指出娃娃身体的各部分：三个部位以上	
122	16.3	粉红模板：放置圆形模块	
123	16.6	兰色模板：放置两个圆形模块	
124	17.2	用笔模仿画一划	
125	17.5	在 42 秒钟内插完桩钉	
126	17.6	说出一物名	

For peer review only

127	17.7	对娃娃执行指令（在通过部位打钩：椅、杯、鼻）	
128	18.1	用语言表达要求	
129	18.6	不用于一划的乱写？	
130	18.8	兰色模板：放置两个圆快和方块	
131	18.8	指出三幅画	
132	19.1	能说两个单词的句子	
133	19.2	说出一副画名	
134	19.2	说出两幅画名	
135	19.3	找出两物	
136	19.8	在 30 秒钟内插完桩钉	
137	20.4	粉红模板：完成	
138	20.4	搭六层塔	
139	20.5	兰色模板，放置六个模块	
140	21	指出五副画	
141	21.1	说出三物名	
142	21.2	勉强合格地安装破娃娃	
143	21.2	区别两物：杯、盘、盒	
144	22.8	辨认钟表：第四张图 1，2，3，4，5	
145	22.9	说出三幅画名	
146	23.8	粉红模板（反转）	
147	24.3	近似地安装破娃娃	
148	24.6	区别三物：杯、盘、盒	
149	24.7	兰色模板，在 150 秒钟内完成	
150	25	搭八层塔	
151	25.1	指出七副画	
152	25.1	用方木搭火车	
153	25.7	说出五副画名	
154	26.3	模仿笔划：垂直线和水平线	
155	27.1	辨认钟表：第 2 张图	
156	27.6	理解两个方位词	
157	28	在 22 秒钟内插完桩钉	
158	28.5	兰色模板：90 秒钟内完成	
159	29.5	折纸	
160	29.6	兰色模板：60 秒钟内完成	
161	30+	正确安装破娃娃	
162	30+	“—”的概念	
163	30+	理解三个方位词	

For peer review only

eTable 2 Psychomotor Development Index (Chinese version)

运动量表

（※可偶尔观察到，△可在施测智力量表时观察到）

序号	月龄	条目	计分
1	0.1	抱起靠肩时抬头	
2	0.1	抱起靠肩时调整姿势	
3	0.1	侧头	
4	0.1	爬起	
5	0.8	△保留红环	
6	0.8	※伸臂玩耍	
7	0.8	※踢腿玩耍	
8	0.8	头起竖起：垂直位	
9	1.6	头部稳定地竖起	
10	1.7	抬头（背悬位）	
11	1.8	由侧卧转向仰卧	
12	2.2	在俯卧位时用双臂撑起自己	
13	2.2	支撑下坐起	
14	2.5	保持头部稳定	
15	2.6	※双手张开占优势	
16	3.3	头平衡	
17	3.4	※尺侧一手掌抓握方木	
18	3.5	轻度支撑坐位	
19	4.3	※由仰卧转向侧卧	
20	4.7	努力想坐起	
21	5.0	部分的拇指相对（桡侧一手掌）拾起方木	
22	5.1	独坐片刻	
23	5.1	※单手抽取	
24	5.2	※转腕	
25	5.2	牵拉坐起	
26	5.6	△试图获取小糖丸	
27	5.7	独立 30 秒钟或以上	
28	5.8	由仰卧转向俯卧	
29	6.2	稳定地独坐	
30	6.5	独坐时协调好	
31	6.6	※舀起小糖丸	
32	6.6	△完全的拇指相对拾起方木	
33	7	早期跨步运动	
34	7.5	牵拉站起	
35	7.6	※不完全的拇指相对抓糖丸	
36	7.6	走路之前的行进方式（俯卧、手膝、手足、其他）	
37	8.3	使两个勺子或方木在中线相碰	
38	8.5	跨步运动	

39	8.6	自己坐起	
40	8.6	借助家具站起	
41	8.9	精细地抓糖丸（灵巧地钳夹）	
42	9.6	拍手（中线技巧）	
43	9.8	坐下	
44	10	扶助下行走	
45	11.1	独站	
46	12	投球	
47	12.1	独走	
48	12.4	起立 I	
49	13.2	扶助下右足独站	
50	13.7	扶助下左足独站	
51	14.1	侧身走	
52	14.5	扶助上楼梯	
53	14.7	倒退走	
54	15.1	扶助下楼梯	
55	17.6	试图站在行木上	
56	18.7	左足独站	
57	19.3	单足踏在行木上走	
58	19.9	起立 II	
59	20.1	右足独站	
60	21.1	走直线：大致方向	
61	23.1	行木：双足站立	
62	24	踮脚走几步	
63	24.3	独自上楼梯：双足	
64	24.4	双足跳离地面	
65	25.3	独自下楼梯	
66	25.6	行木：企图跨步	
67	25.6	倒行两米半	
68	25.7	自第一级台阶下跳下	
69	29.2	自第二级台阶下跳下	
70	29.8	踮脚走两米半	
71	29.9	跳远：10 至 35cm（记录距离）	
72	30+	起立：III	
73	30+	上楼梯：双足交替向前	
74	30+	行木：交替步伐走部分路程	
75	30+	保持双足走在直线上（两米半）	
76	30+	跳远：35cm 至 60cm	
77	30+	跳过：5cm 高的绳子	
78	30+	跳远：60cm 至 85cm	
79	30+	独脚跳两次以上	
80	30+	下楼梯：双足交替向前	
81	30+	跳过 20cm 高的绳子	

For peer review only

For peer review only

eTable 3 Distributions of PM2.5 and NO2 exposure level in 90 days prior to conception, each trimester (T1, T2, and T3) and combined across whole pregnancy period (WP) (n = 1,174)

	Estimated exposure (μg/m³)						
	N	Minimum	25th percentile	Mean ± SD	Median ± IQR	75th percentile	Maximum
Estimated exposure to $PM_{2.5}$							
90 days prior to conception	1,174	38.17	44.00	52.91 ± 10.99	48.43 ± 18.07	62.06	80.53
First trimester	1,174	37.26	43.77	52.07 ± 10.98	47.26 ± 17.31	61.08	82.41
Second trimester	1,174	38.46	47.57	58.64 ± 12.21	57.97 ± 19.62	67.19	90.02
Third trimester	1,174	37.03	47.25	61.83 ± 16.04	58.82 ± 28.7	75.95	96.48
Total pregnancy	1,174	46.69	54.85	57.48 ± 3.97	57.31 ± 5.76	60.61	66.98
Estimated exposure to NO_2							
90 days prior to conception	1,174	25.86	45.49	49.59 ± 6.34	49.94 ± 8.27	53.76	70.48
First trimester	1,174	20.81	44.60	48.8 ± 6.43	48.92 ± 8.51	53.10	69.31
Second trimester	1,174	28.93	47.18	50.98 ± 6.23	51.20 ± 7.72	54.90	70.42
Third trimester	1,174	20.57	47.20	51.79 ± 6.78	52.45 ± 9.47	56.67	75.12
Total pregnancy	1,174	27.50	47.89	50.52 ± 5.08	50.46 ± 5.51	53.40	67.53

eTable 4 Pearson's correlations of $PM_{2.5}$ and NO_2 between each of the five different pregnancy time periods (N = 1,174)

Estimated exposure to		$PM_{2.5}$					NO_2				
		90 days prior to conception	First trimester	Second trimester	Third trimester	Total pregnancy	90 days prior to conception	First trimester	Second trimester	Third trimester	Total pregnancy
$PM_{2.5}$	90 days prior to conception	1									
	First trimester	-0.065	1								
	Second trimester	-0.779	-0.2012	1							
	Third trimester	0.288	-0.7613	-0.1688	1						
	Total pregnancy	-0.534	-0.2709	0.6838	0.3858	1					
NO_2	90 days prior to conception	0.6383	0.0684	-0.4588	0.3714	0.0376	1				
	First trimester	0.1537	0.6352	-0.0159	-0.4927	-0.0633	0.5545	1			
	Second trimester	-0.431	0.0714	0.6269	-0.0133	0.7251	0.3345	0.5399	1		
	Third trimester	0.3027	-0.5213	0.0528	0.6817	0.4432	0.7149	0.2159	0.5145	1	
	Total pregnancy	0.0057	0.0781	0.2862	0.0737	0.4779	0.6786	0.7435	0.8755	0.7331	1

For peer review only

eTable 5 Associations between $PM_{2.5}$ and NO_2 exposure in different pregnancy periods and adverse birth outcomes (unadjusted models)

Per IQR increase in		Mean difference		Odd ratios			
		Birth weight, grams (95% CI) (N=941)	Birth length, cm (95% CI) (N=927)	PTB (case: 33) (95% CI) (N=945)	LBW (case: 30) (95% CI) (N=945)	LGA (case: 108) (95% CI) (N=945)	SGA (case: 84) (95% CI) (N=945)
Estimated exposure to $PM_{2.5}$	90 days prior to conception	9.28 (-31.26, 49.83)	-0.09 (-0.27, 0.09)	0.98 (0.56, 1.74)	1.35 (0.77, 2.36)	1.2 (0.87, 1.64)	0.98 (0.68, 1.41)
	First trimester	21.95 (-16.90, 60.80)	0.14 (-0.03, 0.31)	0.98 (0.57, 1.70)	1 (0.57, 1.77)	0.97 (0.71, 1.33)	0.78 (0.54, 1.13)
	Second trimester	-18.21 (-57.78, 21.37)	0.04 (-0.13, 0.21)	0.85 (0.48, 1.50)	0.61 (0.32, 1.15)	0.92 (0.67, 1.27)	1.33 (0.94, 1.89)
	Third trimester	-37.38 (-81.43, 6.68)	**-0.32 (-0.51, -0.13)**	1.35 (0.74, 2.47)	1.51 (0.80, 2.85)	1.08 (0.76, 1.54)	1.12 (0.76, 1.66)
	Total pregnancy	-20.02 (-55.69, 15.65)	-0.1 (-0.26, 0.05)	0.81 (0.49, 1.33)	0.69 (0.41, 1.16)	1 (0.75, 1.34)	1.2 (0.87, 1.66)
Estimated exposure to NO_2	90 days prior to conception	-13.23 (-45.50, 19.03)	-0.12 (-0.26, 0.02)	1.2 (0.76, 1.89)	1.62 (0.99, 2.65)	1.21 (0.93, 1.57)	1.24 (0.92, 1.66)
	First trimester	0.3 (-32.36, 32.96)	0.08 (-0.06, 0.22)	1.01 (0.64, 1.60)	1.15 (0.71, 1.86)	1.17 (0.90, 1.52)	1.27 (0.94, 1.71)
	Second trimester	-22.85 (-53.39, 7.70)	-0.04 (-0.17, 0.09)	1.11 (0.72, 1.70)	1.08 (0.69, 1.69)	1.06 (0.83, 1.36)	**1.46 (1.10, 1.93)**
	Third trimester	-32.72 (-67.16, 1.72)	**-0.16 (-0.32, -0.01)**	1.13 (0.69, 1.85)	1.35 (0.80, 2.28)	1.24 (0.94, 1.65)	**1.58 (1.14, 2.18)**
	Total pregnancy	-16.58 (-43.35, 10.20)	-0.03 (-0.15, 0.09)	1.03 (0.71, 1.50)	1.13 (0.76, 1.69)	1.16 (0.93, 1.44)	**1.44 (1.13, 1.85)**

All significant findings in the table are bold.

For peer review only

eTable 6 Associations between $PM_{2.5}$ and NO_2 exposure in different pregnancy periods and adverse birth outcomes (co-exposure models)

Per IQR increase in		Mean difference		Odd ratios			
		Birth weight, grams (95% CI) (N=941)	Birth length, cm (95% CI) (N=927)	PTB (case: 33) (95% CI) (N=945)	LBW (case: 30) (95% CI) (N=945)	LGA (case: 108) (95% CI) (N=945)	SGA (case: 84) (95% CI) (N=945)
Estimated exposure to $PM_{2.5}$	90 days prior to conception	75.00 (-9.86, 159.86)	0.23 (-0.14, 0.60)	0.98 (0.56, 1.89)	0.41 (0.14, 1.22)	1.14 (0.55, 2.40)	1.18 (0.48, 2.92)
	First trimester	19.59 (-71.23, 110.41)	0.00 (-0.40, 0.39)	0.97 (0.26, 3.65)	0.66 (0.14, 3.05)	0.67 (0.32, 1.42)	0.73 (0.28, 1.93)
	Second trimester	-25.62 (-104.32, 53.09)	0.08 (-0.26, 0.42)	1.34 (0.37, 4.86)	0.94 (0.21, 4.21)	0.83 (0.42, 1.62)	0.69 (0.34, 1.40)
	Third trimester	13.77 (-72.33, 99.86)	-0.2 (-0.58, 0.17)	1.12 (0.31, 4.07)	0.94 (0.26, 3.35)	1.00 (0.48, 2.12)	0.57 (0.26, 1.23)
	Total pregnancy	21.13 (-36.41, 78.67)	0.02 (-0.23, 0.27)	0.73 (0.33, 1.61)	0.52 (0.24, 1.15)	0.98 (0.60, 1.61)	**0.55 (0.32, 0.96)**
Estimated exposure to NO_2	90 days prior to conception	-18.63 (-64.02, 26.76)	-0.09 (-0.29, 0.10)	1.24 (0.61, 2.49)	1.3 (0.64, 2.64)	1.27 (0.84, 1.90)	1.39 (0.90, 2.15)
	First trimester	-14.53 (-61.15, 32.09)	0.05 (-0.16, 0.25)	0.91 (0.45, 1.83)	1.14 (0.55, 2.37)	1.33 (0.89, 2.00)	**1.70 (1.07, 2.69)**
	Second trimester	-14.46 (-57.45, 28.54)	-0.08 (-0.26, 0.11)	1.22 (0.63, 2.36)	1.36 (0.68, 2.71)	1.27 (0.87, 1.87)	1.50 (1.00, 2.24)
	Third trimester	-13.13 (-64.87, 38.62)	0.04 (-0.18, 0.27)	0.77 (0.35, 1.67)	0.97 (0.43, 2.16)	1.41 (0.90, 2.23)	**1.77 (1.08, 2.91)**
	Total pregnancy	-15.02 (-49.33, 19.30)	0.00 (-0.15, 0.15)	1.08 (0.64, 1.80)	1.28 (0.75, 2.18)	1.21 (0.90, 1.63)	**1.60 (1.15, 2.23)**

All significant findings in the table are bold.

Models adjusted for maternal age at enrolment, infant's sex, maternal BMI at 11–14 weeks' gestation, primiparity, monthly household income level, and season of births, and adjusted for the other air pollutant

For peer review only

eTable 7 Comparison of major confounders and outcomes for those with missing and non-missing outcome data and non-missing confounder data

	Excluded from analyses* (n=256)	Included in analyses (n = 1,017)	p-value
**Maternal age, mean ± SD**	28.47 ± 0.22	28.78 ± 0.11	0.22
**Gestational week, mean ± SD**	39.34 ± 0.12	39.39 ± 0.04	0.69
**Maternal BMI (kg/m^2), mean ± SD**	21.13 ± 0.17	21.56 ± 0.09	**0.03**
**Infant's sex**			
Female, n (%)	136 (53.33)	527 (51.87)	0.676
Male, n (%)	119 (46.67)	489 (48.13)	
**Primiparity**			0.583
Yes, n (%)	226 (22.22)	791 (77.78)	
No, n (%)	61 (23.83)	195 (76.17)	
**Season**			**0.00**
Spring (Mar-May), n (%)	74 (32.46)	337 (35.62)	
Summer (Jun-Aug), n (%)	74 (32.46)	189 (19.98)	
Autumn (Sep-Nov), n (%)	55 (24.12)	143 (15.12)	
Winter (Dec-Feb), n (%)	25 (10.96)	277 (29.28)	

*Excluded from analyses due to missing cognitive outcome data

For peer review only

BMJ Open

Associations of air pollution exposures in preconception and pregnancy with birth outcomes and infant neurocognitive development: analysis of the Complex Lipids in Mothers and Babies (CLIMB) prospective cohort in Chongqing, China

Journal:	*BMJ Open*
Manuscript ID	bmjopen-2023-082475.R2
Article Type:	Original research
Date Submitted by the Author:	08-May-2024

Complete List of Authors:	Chen, Yingxin; University of Leicester, Centre for Environmental Health and Sustainability<br>Kuang, Tao; Zunyi Medical and Pharmaceutical College, Department of public health and management<br>Zhang, Ting; Stomatological Hospital of Chongqing Medical University<br>Cai, Samuel; University of Leicester, Department of Health Sciences<br>Colombo, John ; University of Kansas<br>Harper, Alex; University of Leicester<br>Han, Ting-Li; University of Auckland Liggins Institute; Canada - China - New Zealand Joint Laboratory of Maternal and Fetal Medicine<br>xia, yinyin; Chongqing Medical University<br>Gulliver, John; St George's University of London<br>Hansell, Anna ; University of Leicester, Centre for Environmental Health and Sustainability<br>Zhang, Hua; The First Affiliated Hospital of Chongqing Medical University, Department of Obstetrics and Gynaecology<br>Baker, Philip ; University of Leicester, College of Medicine

**Primary Subject Heading**:	Epidemiology
Secondary Subject Heading:	Obstetrics and gynaecology
Keywords:	China, Developmental neurology & neurodisability < PAEDIATRICS, Child & adolescent psychiatry < PSYCHIATRY, EPIDEMIOLOGIC STUDIES, EPIDEMIOLOGY, Maternal medicine < OBSTETRICS

SCHOLARONE™
Manuscripts

BMJ

I, the Submitting Author has the right to grant and does grant on behalf of all authors of the Work (as defined in the below author licence), an exclusive licence and/or a non-exclusive licence for contributions from authors who are: i) UK Crown employees; ii) where BMJ has agreed a CC-BY licence shall apply, and/or iii) in accordance with the terms applicable for US Federal Government officers or employees acting as part of their official duties; on a worldwide, perpetual, irrevocable, royalty-free basis to BMJ Publishing Group Ltd ("BMJ") its licensees and where the relevant Journal is co-owned by BMJ to the co-owners of the Journal, to publish the Work in this journal and any other BMJ products and to exploit all rights, as set out in our licence.

The Submitting Author accepts and understands that any supply made under these terms is made by BMJ to the Submitting Author unless you are acting as an employee on behalf of your employer or a postgraduate student of an affiliated institution which is paying any applicable article publishing charge ("APC") for Open Access articles. Where the Submitting Author wishes to make the Work available on an Open Access basis (and intends to pay the relevant APC), the terms of reuse of such Open Access shall be governed by a Creative Commons licence – details of these licences and which Creative Commons licence will apply to this Work are set out in our licence referred to above.

Other than as permitted in any relevant BMJ Author's Self Archiving Policies, I confirm this Work has not been accepted for publication elsewhere, is not being considered for publication elsewhere and does not duplicate material already published. I confirm all authors consent to publication of this Work and authorise the granting of this licence.

For peer review only

Associations of air pollution exposures in preconception and pregnancy with birth outcomes and infant neurocognitive development: analysis of the Complex Lipids in Mothers and Babies (CLIMB) prospective cohort in Chongqing, China

Yingxin Chen[1,2], Tao Kuang[3], Ting Zhang[4], Yutong Samuel Cai[1,2, 5], John Colombo[6], Alex Harper[7], Ting-Li Han[8], Yinyin Xia[9], John Gulliver[10], Anna L Hansell[1,2,5], Hua Zhang[8], Philip N Baker[7,8]

1: Centre for Environmental Health and Sustainability, University of Leicester, Leicester, UK

2: The National Institute of Health Research (NIHR) Health Protection Research Unit (HPRU) in Environmental Exposure and Health at the University of Leicester, Leicester, UK

3: Department of public health and management, Zunyi Medical and Pharmaceutical College, Zunyi, 563000, Guizhou, China

4. Stomatological Hospital of Chongqing Medical University, Chongqing, 401147, China

5: NIHR Leicester Biomedical Research Centre, Leicester General Hospital, Leicester, UK

6: Schiefelbusch Institute for Life Span Studies and Department of Psychology, University of Kansas, Lawrence, KS 66045, USA

7: College of Life Sciences, University of Leicester, Leicester, UK

8: Department of Obstetrics and Gynaecology, the First Affiliated Hospital of Chongqing Medical University, Chongqing, 400016, China

9: School of Public Health, Chongqing Medical University, Chongqing, 400016, China

10: Environmental and Exposure Sciences, Population Health Research Institute, St George's, University of London, London, UK

Correspondence to:

Yinyin Xia. School of Public Health, Chongqing Medical University, Chongqing, 400016, China.

Email: 100118@cqmu.edu.cn

For peer review only

For peer review only - http://bmjopen.bmj.com/site/about/guidelines.xhtml

Abstract

Objectives: To investigate the associations of traffic-related air pollution exposures in early pregnancy with birth outcomes and infant neurocognitive development.

Design: Cohort study.

Setting: Eligible women attended six visits in the maternity clinics of two centres, the First Affiliated Hospital of Chongqing Medical University and Chongqing Health Centre for Women and Children.

Participants: Women who were between 20 and 40 years of age and were at 11–14 weeks gestation with a singleton pregnancy were eligible for participation. Women were excluded if they had a history of premature delivery before 32 weeks of gestation, maternal milk allergy or aversion, or severe lactose intolerance. 1,273 pregnant women enrolled in 2015-2016 and 1,174 live births were included in this analysis.

Exposures: Air pollution concentrations at their home addresses, including particulate matter (PM) with diameter ≤2.5μm ($PM_{2.5}$) and nitrogen dioxide (NO_2), during pre-conception and each trimester period were estimated using land-use regression models.

Outcome measures: Birth outcomes (i.e., birthweight, birth length, preterm birth (PTB), low birth weight (LBW), large for gestational age (LGA) and small for gestational age (SGA) status) and neurodevelopment outcomes measured by the Chinese version of Bayley Scales of Infant Development (CBSID).

Results: An association between SGA and per-interquartile range (IQR) increases in NO_2 was found in the first trimester (odds ratio (OR): 1.57, 95% confidence interval (CI): 1.06, 2.32) and during the whole pregnancy (OR: 1.33, 99% CI: 1.01, 1.75). Both $PM_{2.5}$ and NO_2 exposure in the 90 days prior to conception were associated with lower Psychomotor Development Index (PDI) scores (β: -6.15, 95% CI: -8.84, -3.46; β: -2.83, 95% CI: -4.27, -1.39, respectively). Increased NO_2 exposure was associated with an increased risk of psychomotor development delay (PDD) during different trimesters of pregnancy.

Conclusions: Increased exposures to NO_2 during pregnancy were associated with increased risks of SGA and psychomotor development delay, while increased exposures to both $PM_{2.5}$ and NO_2 pre-conception were associated with adverse psychomotor development outcomes at 12 months of age.

For peer review only - http://bmjopen.bmj.com/site/about/guidelines.xhtml

57

58 **Keywords:** Air pollution; birth outcomes; child cognition

For peer review only

For peer review only - http://bmjopen.bmj.com/site/about/guidelines.xhtml

Article summary

Strengths and limitations of this study

- This study uniquely explored the impacts of both pre-conception and prenatal exposure to $PM_{2.5}$ and NO_2 on neurodevelopmental outcomes in young infants, within an urban environment characterized by relatively high air pollution levels.
- We developed an LUR model to capture spatial and temporal variations of air pollution at individual level to reduce exposure misclassification.
- Our sample size was relatively small, limiting the statistical power to assess several outcomes.
- We defined exposure windows for clinically-defined trimesters; sensitive periods may be shorter or longer than 3 months, or may exist in the overlap of multiple trimesters.

For peer review only

For peer review only - http://bmjopen.bmj.com/site/about/guidelines.xhtml

INTRODUCTION

Air pollution is a major environmental factor that has been linked to a range of adverse health outcomes in children. Maternal exposure to air pollutants during pregnancy, especially particulate matter (PM) with diameter ≤2.5μm ($PM_{2.5}$) and nitrogen dioxide (NO_2), has been found to be associated with adverse birth outcomes, including pre-term birth (PTB)(1), term low birth weight (TLBW) (2), and small for gestational age (SGA) status (3). According to the developmental origins of health and disease (DOHaD) hypothesis, prenatal exposures to air pollution may lead to adverse birth outcomes and subsequently increase the susceptibility to the development of certain diseases later in life (4). A number of epidemiological studies have linked prenatal air pollution exposure with neurodevelopmental disorders such as autism spectrum disorder (ASD), attention deficit hyperactivity disorder (ADHD), and cognitive impairment (5). Although the underlying biological mechanisms are still unclear, some studies indicated that prenatal air pollution exposure may induce systemic oxidative stress that triggers intrauterine inflammation, leading to damage to several fetal organs, including the brain (6, 7).

It is also unclear that whether the adverse effects of air pollution may start earlier before conception. Three months before conception was considered as a critical developmental window for gametogenesis. Air pollution exposure during the three-month preconception period may have adverse effects on gametogenesis of sperm (8, 9) and ova cells (10). Exposures to $PM_{2.5}$ in preconception period have been associated with various neurodevelopmental outcomes, such as neural tube defects (11), lower psychomotor development scores (12), higher risk of ASD (13, 14), and higher risk of intellectual disability (15). Further research is required due to inconsistencies across studies in terms of studied health outcomes and exposure levels of air pollution (12). Additionally, while there is growing evidence for the effects of preconception $PM_{2.5}$ exposure on the risk of adverse neurodevelopmental outcomes, no study to date has examined the effects of preconception NO_2 exposure. Exposure to NO_2 during pregnancy may be linked to compromised neural development in children, particularly affecting fine psychomotor skills (16). Studying $PM_{2.5}$ along with NO_2 may allow us to explore how multiple pollutants affect birth outcomes and infant neurocognitive development independently and jointly. Moreover, both $PM_{2.5}$ and NO_2 are regulated traffic-related air pollutants in many countries. Understanding their impacts on birth and infant neurocognitive development can provide valuable insights for policymakers and public health authorities to develop effective air quality regulations and interventions.

For peer review only - http://bmjopen.bmj.com/site/about/guidelines.xhtml

Many studies have reported the effects of prenatal exposure to air pollution on neurodevelopmental function in children. However, the reported associations vary, due to the heterogeneous assessments of air pollution and neurodevelopmental outcomes (5, 17).

The current study leveraged the Complex Lipids in Mothers and Babies (CLIMB) cohort, a prospective birth cohort recruited in Chongqing, China (18), with trimester-specific maternal $PM_{2.5}$ and NO_2 air pollution exposure derived from a spatio-temporal land use regression (LUR) model (19). The aim of this analysis was to examine the associations between $PM_{2.5}$ and NO_2 exposures during pre- and during pregnancy, with birth and infant neurocognitive development outcomes at 12 months of age.

A key aspect in all studies like this one is the accuracy of documenting exposure; a recent Chinese study determined air pollution exposure based on data from the nearest monitoring station (20) may not reflect the fine temporal and spatial variability of pollutant exposures among participants. Our study employed common air pollution exposure models based on advanced geographic information systems (GIS), to address some of the limitations of previous studies (21).

In addition, the timing of exposure is also critical in determining the effects of exposure on developmental outcomes. Indeed, the evidence from previous studies on the sensitive time windows for exposure pre- and during pregnancy remains inconclusive. Some studies have indicated that the early-to-mid pregnancy phase may be a critical period in terms of the impact of air pollution on neurodevelopment(22, 23). Early pregnancy is particularly important for neurogenesis and neuromigration, making it a susceptible period (24). However, some studies reported stronger associations for middle or late pregnancy (20, 25, 26). More studies identifying critical periods are needed to enhance our understanding of how pre-conception and prenatal air pollution exposure affect neurodevelopment. With this cohort, we are able to examine the effects of exposure pre-conception, at each trimester, and the entire pregnancy.

For peer review only - http://bmjopen.bmj.com/site/about/guidelines.xhtml

METHODS

Study population

Participant recruitment in the CLIMB cohort has been described previously (27). In brief, women who were between 20 and 40 years of age and were at 11–14 weeks gestation with a singleton pregnancy were eligible for participation. Women were excluded if they had a self-stated history of premature delivery before 32 weeks of gestation, maternal milk allergy or aversion, or severe lactose intolerance.

From September 2015 to November 2016, a total of 1,500 women were recruited into the cohort. Participants attended six visits at the First Affiliated Hospital of Chongqing Medical University and Chongqing Health Centre for Women and Children: 11–14 weeks' gestation (visit 1), 22–28 week's gestation (visit 2), 32–34 week's gestation (visit 3), at birth (visit 4), 6 weeks postnatal (visit 5), and 12 months postnatal (visit 6).

Women who withdrew from the study (n = 146), terminated their pregnancy (n = 29), miscarried (n = 12) or were lost to follow-up (n = 40) were excluded from the analysis, leaving a sample size of 1,273 women. Analyses were restricted to mothers whose detailed residential addresses during pregnancy were known (**Figure 1**). A total of 1,174 live births were thus included in the pregnancy and neonatal outcomes analysis. Subsequently, at 1 year follow-up, 946 children were included in the analysis of neurodevelopment outcomes.

Study setting

The study area focused on the urban center of the Chinese municipality of Chongqing (**Figure 2**). The terrain of Chongqing is predominantly hilly and mountainous, with the core area located in a synclinal valley at the confluence of the Yangtze River and the Jialing River (28). The urban core of Chongqing, our study area, has a population of approximately 6.52 million people, a land area of 5,472 square kilometers, and 4.62 million vehicles (29). It shows a higher population density of approximately 1,191 people per square kilometer and a lower number of motor vehicles of 0.71 per capita. The urban core of Chongqing used to have multiple old industries with higher NO_2 and $PM_{2.5}$ emissions, including the Chongqing Iron and Steel Company in Dadukou District and the Chongqing Thermal Power Plant in Jiulongpo District, both of which have been relocated to rural areas in Chongqing. The main sources of pollution in the area now include traffic-related emissions, construction activities, and anthropogenic sources such as outdoor grilling and emissions from food establishments (30). The coverage

For peer review only - http://bmjopen.bmj.com/site/about/guidelines.xhtml

160 rate of urban population with access to gas in Chongqing was 95.34% (29), suggesting a low reliance on biomass cookstoves in urban areas.

162 **Exposure assessment**

163 The address of participants was collected at the first visit. Exposure assessment based on spatiotemporal land use regression (LUR) models for $PM_{2.5}$ and NO_2 were developed for the study region. A description of the methodology of exposure modelling has been reported previously (19). Briefly, the models included both spatial and temporal components of exposure. $PM_{2.5}$ and NO_2 concentration data were collected from 17 routine monitoring sites operated by the Chongqing Environmental Monitoring Center in 2015-2016. For the spatial component of models, we calculated annual average concentrations of each pollutant in 2015, and fit linear regression models using five groups of geographic data (road network, land use, topography, vegetation, and population density) as spatial predictor variables. For the temporal component of models, we calculated the residuals from the spatial component at each monitoring site on a daily basis by subtracting the predicted annual average concentration from the observed daily average concentrations measured in 2015 and 2016, and then fitted generalised additive models (GAM) using seven groups of meteorological data (temperature, amount of rainfall, rainfall events, relative humidity, horizontal visibility, wind direction and wind speed) as temporal predictor variables. The meteorological variables were used to account for the influence of weather on the change in air pollution concentration over time. To account for the remaining spatial autocorrelation, the smoothed terms of longitude and latitude were fit to spatiotemporal residuals which were calculated by subtracting the sum of the spatial temporal predictions from the measured daily average concentrations in 2015 and 2016. The performance of the $PM_{2.5}$ spatiotemporal models was good (Correlation (COR)-R^2: 0.72) and the NO_2 spatiotemporal model was low (COR-R^2: 0.39) when providing concentration estimates in absolute terms.

185 Combining the family address coordinates of each pregnant woman and the gestation period of the pregnancy (calculated from the date of last menstrual period to the date of delivery), we used this spatiotemporal model to estimate the average exposure of each pregnant woman in 90 days prior to pregnancy (90D), first trimester (T1), second trimester (T2), third trimester (T3) and whole pregnancy period (WP), respectively.

190 **Outcomes**

191 ***Birth outcomes***

For peer review only - http://bmjopen.bmj.com/site/about/guidelines.xhtml

Birth outcomes were determined by experienced obstetricians and abstracted from the medical records. Birth outcomes included: birthweight (in grams), birth length (in centimetres), PTB, low birth weight (LBW), large for gestational age (LGA) and SGA status (31). PTB was defined as delivery before 37 weeks. LBW was defined as weighing less than 2500 g at birth. LGA and SGA were indicated by birth weight greater than and less than the 90th and 10th percentile within this study for the gestational age by sex respectively (32). Term low birth weight was not considered due to a small sample size of only 8 cases.

Neurodevelopment outcomes

The Chinese version of Bayley Scales of Infant Development (CBSID) was used to assess mental and psychomotor development for infants in this study. The CBSID is appropriate for evaluation of infants from 2–30 months old (33) and takes into consideration each infant's age in days. Infants were assessed at around 12 months (range from 11 months and 15 days to 12 months and 15 days) by a trained examiner, with ages corrected for preterm birth. These scales have been formally adapted to the Chinese language and locally standardized to become culturally appropriate, with two main indexes: the Mental Development Index (MDI) and the Psychomotor Development Index (PDI). The MDI component comprised 163 items and assessed age-appropriate items related to cognitive functioning, personal and social development, and language development (see **eTable 1** in the Supplement). The PDI component comprised 81 items and assessed age-appropriate fine and gross motor skills (see **eTable 2** in the Supplement). The test provided raw scores for mental and psychomotor development that were converted to standardized (in terms of age in days) MDI and PDI scores, based on norms for the Chinese population. As with other forms of the Bayley test these index scores have a mean of 100, and a standard deviation of 15, with a lower score reflecting poorer performance (34). If an infant refused to cooperate with the examiners to finish the task, a second assessment was arranged within two weeks. If the infant could not cooperate at the second BSID assessment, their data were classified as missing. In addition to the continuous scores, we define mental developmental delay (MDD) and psychomotor developmental delay (PDD) if the score is less than 85 (35).

Covariates

Socio-demographic data were collected through interviews by trained nurses. The following potential confounders were identified: maternal age at enrolment (in years), infant sex (male/female), maternal BMI at 11–14 weeks' gestation (kg/m^2), parity (Yes/No), monthly

For peer review only - http://bmjopen.bmj.com/site/about/guidelines.xhtml

household income level (categorized as: <2,000 yuan, 2,000–7,000 yuan, 7,000–10,000 yuan, or >10,000 yuan), season of birth (categorized as: Spring (Mar-May), Summer (Jun-Aug), Autumn (Sep-Nov) or Winter (Dec-Feb)). Season of birth was taken into consideration because air pollution and related environmental factors, such as temperature and humidity, may vary across different seasons (i.e., air pollution levels tend to be higher during winter). Some studies suggest that the season of birth may indirectly influence cognitive function through factors such as seasonal differences in food availability affecting maternal nutrition during pregnancy, sunlight exposure impacting maternal vitamin D levels, and children's early-life indoor and outdoor activities. Marital status (Single/married) and smoking or drinking during pregnancy (Yes/no) were not taken into account in this analysis because of the homogeneity of the study population (i.e., 98.6% women were married and 99.6% women reported not smoking or drinking alcohol during pregnancy). We did not adjust dietary supplements during pregnancy because all pregnant women routinely take folic acid in this cohort.

Statistical analyses

Data were described in terms of mean ± SD or median (IQR) for continuous variables, or as percentages for categorical variables. Modelled $PM_{2.5}$ and NO_2 exposure levels in 90D, T1, T2, T3 and WP were considered separately. We examined the Spearman correlation between each of the exposures in the different pregnancy periods. For birth outcomes, multivariable linear regression was used for continuous outcomes (e.g., birth weight and birth length) to estimate β coefficient and their 95% confidence intervals (CIs) and multivariable logistic regression for binary outcomes (e.g., PTB, LBW, LGA and SGA status) to estimate odds ratio (OR) and 95% CIs. For mental and psychomotor development (e.g., MDI and PDI scores), multivariable linear regression models were fit to estimate β coefficient and their 95% CIs. We also conducted multivariable logistic regression analysis for binary neurodevelopment outcomes (i.e., MDD and PDD). Models were adjusted for maternal age at enrolment, infant sex, maternal BMI at 11–14 weeks gestation, primiparity, monthly household income level, and season of birth. We also ran co-exposure models to estimate associations of one air pollutant whilst additionally adjusting for the other air pollutant (i.e., $PM_{2.5}$ effects in T1 adjusted for NO_2 in T1). Effect estimates are reported for each IQR increase of $PM_{2.5}$ and NO_2. All analyses were performed using STATA version 17. A p-value of <0.05 was considered statistically significant to address multiple comparisons in the analyses.

Patient and public involvement

For peer review only - http://bmjopen.bmj.com/site/about/guidelines.xhtml

None.

For peer review only

For peer review only - http://bmjopen.bmj.com/site/about/guidelines.xhtml

RESULTS

Study participants

Participant characteristics are presented in **Table 1**. Of those participating women, the mean age was 28.7 years and mean BMI was 21.5 kg/m^2. 98.0% of women were of Han ethnicity, 77.9% were primiparous, and 67.6% had completed tertiary education. 33 (2.8%), 30 (2.6%), 108 (9.2%), 84 (7.2%) of the 1,174 births considered in this analysis were classified as PTB, LBW, LGA and SGA, respectively. For those 946 children who completed the BSID test, the mean MDI score was 94.7 (SD: 17.7) and the mean PDI score was 87.4 (SD: 14.9). The proportions of participants with MDD (MDI <85) and PDD (PDI < 85) were 27.1% and 42.4%, respectively.

Exposure assessment

Median $PM_{2.5}$ exposure concentrations were 57.31 µg/m^3 (IQR: 5.76) and median NO_2 exposure levels were 50.46 µg/m^3 (IQR: 5.51) during the whole pregnancy period (**eTable 3 in the Supplement**). For $PM_{2.5}$, the concentration in the pre-conception and T1 were considerably lower than other periods, close to 10 µg/m^3. The between-trimester and 90D values for NO_2 were generally moderately correlated (Pearson's r > 0.5). The correlation coefficients of $PM_{2.5}$ were more variable between time periods reflecting the high variability of $PM_{2.5}$ concentrations, with values ranging from -0.78 to +0.68. Correlations between $PM_{2.5}$ and NO_2 in the same pregnancy period were moderately correlated (Pearson's r ~0.6, **eTable 4 in the Supplement**).

For peer review only - http://bmjopen.bmj.com/site/about/guidelines.xhtml

For peer review only

Table 1. Characteristics of study sample in the CLIMB cohort (N = 1,174)

Characteristic of mother	N	n (%) / mean ± SD
Maternal age (Years)	1,174	28.7 ± 3.5
BMI (kg/m^2)	1,174	21.5 ± 2.9
Han ethnicity (%)	1,174	
Yes		1,151 (98.0%)
No		23 (2.0%)
Marital status (%)	1,174	
Single		16 (1.4%)
Married		1,158 (98.6%)
Primiparity (%)	1,174	
Yes		914 (77.9%)
No		260 (22.1%)
History of miscarriage or abortion (%)	1,174	
Yes		553 (47.1%)
No		621 (52.9%)
Smoking/drinking during pregnancy (%)	1,174	
Yes		5 (0.4%)
No		1,169 (99.6%)
Education level	946	
Low: High school or below		306 (32.3%)
High: College/uni or above		640 (67.6%)
Job	946	
Full-time		762 (80.5%)
Housewife		82 (8.7%)
Others		102 (10.8%)
Household income (Monthly)	946	
<2000 RMB		186 (19.7%)
2000-4000 RMB		329 (34.8%)
4000-7000 RMB		292 (30.9%)
7000-10000 RMB		139 (14.7%)

Characteristic of child	N	n (%) /mean ± SD
Gestational week (week)	1,174	39.4 ± 1.5
Birth weight (g)	1,165	3314.4 ± 428.8
Birth length (cm)	1,149	49.7 ± 1.9
New born sex	1,172	
Female		561 (47.9%)
Male		611 (52.1%)
Birth outcomes		
Preterm birth (PTB)	1,174	
Yes		33 (2.8%)
No		1,141 (97.2%)
Low birth weight (LBW)	1,174	
Yes		30 (2.6%)
No		1,141 (97.2%)
Large for gestational age (LGA)	1,174	
Yes		108 (9.2%)
No		1,066 (90.8%)
Small for gestational age (SGA)	1,174	
Yes		84 (7.2%)
No		1,090 (92.8%)
BSID test	946	
MDI score		94.7 ± 17.7
PDI score		87.4 ± 14.9
Mental development	946	
Delay (MDI < 85)		276 (27.1%)
Normal (MDI ≥ 85)		741 (72.9%)
Psychomotor Development	946	
Delay (PDI < 85)		431 (42.4%)
Normal (PDI ≥ 85)		586 (57.6%)
Season of birth	1,174	
Spring (Mar-May)		411 (35.01%)
Summer (Jun-Aug)		263 (22.40%)
Autumn (Sep-Nov)		198 (16.87%)
Winter (Dec-Feb)		302 (25.72%)

Association with birth outcomes

In the unadjusted models (**eTable 5 in the Supplement**), higher exposure concentrations of $PM_{2.5}$ in T3 were significantly associated with lower birth length (β: -0.32, 95% CI: -0.51, -0.13; per IQR increase). We also observed increased NO_2 in T3 were significantly associated with lower birth length (β: -0.16, 95% CI: -0.32, -0.01; per IQR). A risk between SGA and increases in NO_2 (per IQR) was found in T2 (OR: 1.46, 95% CI: 1.10, 1.93), T3 (OR: 1.58,

For peer review only

For peer review only - http://bmjopen.bmj.com/site/about/guidelines.xhtml

287 95% CI: 1.14, 2.18) and in the whole pregnancy period (OR: 1.44, 95% CI: 1.13, 1.85). We
288 observed no evidence of associations of NO_2 with overall birth weight, birth length and other
289 adverse birth outcomes (e.g., PTB, LBW, and LGA).

290 In the adjusted models (**Table 2**), we found increased effect size for NO_2 and SGA in T2
291 (OR: 1.57, 95% CI: 1.06, 2.32), and slightly reduced effects size for NO_2 and SGA in the
292 whole pregnancy period (OR: 1.33, 95% CI: 1.01, 1.75) compared with the unadjusted
293 model. We observed no evidence of associations with birth length in the adjusted models.
294 After co-adjustment for $PM_{2.5}$ (**see eTable 6 in the Supplement**), the association of NO_2
295 with SGA was also found in T1 (OR: 1.70, 95% CI: 1.07, 2.69), T3 (OR: 1.77, 95% CI: 1.08,
296 2.91) and in the whole pregnancy period (OR: 1.60, 95% CI: 1.15, 2.23).

For peer review only - http://bmjopen.bmj.com/site/about/guidelines.xhtml

Table 2. Associations between $PM_{2.5}$ and NO_2 exposure in different pregnancy periods and adverse birth outcomes (adjusted models)

Per IQR increase in		Mean difference		Odds ratios			
		Birth weight, grams (95% CI) (N=941)	Birth length, cm (95% CI) (N=927)	PTB (case: 33) (95% CI) (N=945)	LBW (case: 30) (95% CI) (N=945)	LGA (case: 108) (95% CI) (N=945)	SGA (case: 84) (95% CI) (N=945)
Estimated exposure to $PM_{2.5}$	90 days prior to conception	59.73 (-16.52, 135.98)	0.15 (-0.176, 0.48)	0.24 (0.06, 1.00)	0.49 (0.18, 1.29)	1.40 (0.72, 2.71)	1.66 (0.75, 3.68)
	First trimester	6.21 (-73.79, 86.20)	0.04 (-0.308, 0.388)	0.88 (0.28, 2.80)	0.76 (0.21, 2.81)	0.86 (0.45, 1.67)	1.33 (0.58, 3.04)
	Second trimester	-37.64 (-107.73, 32.44)	0.02 (-0.283, 0.326)	1.62 (0.53, 4.96)	1.34 (0.38, 4.68)	1.00 (0.55, 1.83)	0.94 (0.50, 1.76)
	Third trimester	4.20 (-73.17, 81.57)	-0.17 (-0.509, 0.162)	0.92 (0.29, 2.90)	0.92 (0.30, 2.85)	1.29 (0.65, 2.53)	0.83 (0.42, 1.66)
	Total pregnancy	8.01 (-41.10, 57.11)	0.02 (-0.198, 0.230)	0.77 (0.38, 1.54)	0.62 (0.31, 1.25)	1.15 (0.75, 1.77)	0.84 (0.52, 1.35)
Estimated exposure to NO_2	90 days prior to conception	-1.03 (-41.88, 39.81)	-0.04 (-0.215, 0.139)	0.84 (0.45, 1.57)	1.04 (0.54, 1.98)	1.31 (0.91, 1.88)	1.45 (0.99, 2.12)
	First trimester	-9.78 (-50.84, 31.28)	0.04 (-0.133, 0.223)	0.90 (0.49, 1.65)	1.03 (0.56, 1.91)	1.21 (0.85, 1.72)	**1.57 (1.06, 2.32)**
	Second trimester	-20.82 (-59.11, 17.47)	-0.06 (-0.222, 0.112)	1.31 (0.73, 2.34)	1.34 (0.75, 2.40)	1.21 (0.86, 1.70)	1.36 (0.95, 1.95)
	Third trimester	-9.50 (-56.00, 36.99)	-0.01 (-0.213, 0.191)	0.79 (0.40, 1.59)	0.95 (0.47, 1.94)	1.42 (0.94, 2.13)	1.51 (0.97, 2.36)
	Total pregnancy	-8.45 (-37.73, 20.83)	0.00 (-0.125, 0.130)	0.97 (0.62, 1.51)	1.04 (0.66, 1.64)	1.20 (0.93, 1.56)	**1.33 (1.01, 1.75)**

All significant findings in the table are bold.

Models adjusted for maternal age at enrolment, infant's sex, maternal BMI at 11–14 weeks' gestation, primiparity, monthly household income level, and season of births.

For peer review only

For peer review only - http://bmjopen.bmj.com/site/about/guidelines.xhtml

Association with infant neurodevelopment outcomes

In unadjusted models, $PM_{2.5}$ exposure in the 90 days prior to conception was associated with lower MDI and PDI scores in offspring (β: -3.54, 95% CI: -5.37, -1.71; β: -3.42, 95% CI: -4.96, -1.89) (**Table 3**). We also observed an unexpected positive association between $PM_{2.5}$ exposures in second trimester with MDI (β: 4.21, 95% CI: 2.43, 6.00) and PDI (β: 2.63, 95% CI: 1.12, 4.14). Exposure to NO_2 was associated with lower MDI (-1.90, 95% CI: -3.36, -0.44) and PDI in the 90 days prior to conception (-2.86, 95% CI: -4.08, -1.65). NO_2 exposure was also associated with lower PDI scores in T3 (-1.97, 95% CI: -3.29, -0.65) and in the whole pregnancy periods (-1.08, 95% CI: -2.11, -0.05). We did not observe any association between NO_2 and MDI in any pregnancy periods.

In the adjusted models (Table 3), we found $PM_{2.5}$ exposure in the 90 days prior to conception was associated with lower PDI scores (β: -6.15, 95% CI: -8.84, -3.46). Similarly, there was also a significant association of increased NO_2 exposure and lower PDI score in the 90 days prior to conception (β: -2.83, 95% CI: -4.27, -1.39), T1 (β: -1.91, 95% CI: -3.37, -0.46), T3 (β: -1.92, 95% CI: -3.57, -0.26) and whole pregnancy period (β: -1.15, 95% CI: -2.19, -0.11). The positive association between $PM_{2.5}$ exposures in second trimester with PDI (β: 3.76, 95% CI: 1.27, 6.24) remained. We did not observe any association with MDI in any pregnancy periods.

In the co-exposure models (**Table 3**), $PM_{2.5}$ exposure in the 90 days prior to conception was associated with lower PDI scores (β: -4.74, 95% CI: -7.73, -1.75). We also observed a positive association between $PM_{2.5}$ exposures in second trimester with PDI (β: 5.51, 95% CI:2.73, 8.28). Exposure to NO_2 was significantly associated with lower PDI in 90D (β: -1.72, 95% CI: -3.31, -0.12), T1 (β: -1.80, 95% CI: -3.46, -0.15), T2 (β: -2.11, 95% CI: -3.63, -0.60), T3 (β: -1.92, 95% CI: -3.76, -0.09) and whole pregnancy period (β: -1.68, 95% CI: -2.89, -0.46).

For peer review only

For peer review only - http://bmjopen.bmj.com/site/about/guidelines.xhtml

Table 3. Associations between $PM_{2.5}$ and NO_2 exposure in different pregnancy periods and continuous BSID scores

Per IQR increase in		Crude models		Adjusted models*		Co-exposure models**	
		MDI (95% CI) (N=946)	PDI 95% CI (N=946)	MDI (95% CI) (N=945)	PDI 95% CI (N=945)	MDI (95% CI) (N=945)	PDI 95% CI (N=945)
Estimated exposure to $PM_{2.5}$	90 days prior to conception	**-3.54 (-5.37, -1.71)**	**-3.42 (-4.96, -1.89)**	-1.98 (-5.19, 1.23)	**-6.15 (-8.84, -3.46)**	-1.73 (-5.30, 1.85)	**-4.74 (-7.73, -1.75)**
	First trimester	-1.07 (-2.93, 0.79)	0.04 (-1.52, 1.61)	-1.66 (-5.02, 1.70)	-2.11 (-4.95, 0.73)	-2.84 (-6.65, 0.97)	-0.45 (-3.67, 2.76)
	Second trimester	**4.21 (2.43, 6.00)**	**2.63 (1.12, 4.14)**	3.79 (0.85, 6.73)	**3.76 (1.27, 6.24)**	4.19 (0.89, 7.49)	**5.51 (2.73, 8.28)**
	Third trimester	-1.43 (-3.41, 0.55)	**-1.76 (-3.42, -0.10)**	-2.73 (-5.99, 0.53)	-1.37 (-4.12, 1.39)	-3.84 (-7.46, -0.22)	0.04 (-3.02, 3.09)
	Total pregnancy	**1.64 (0.06, 3.21)**	0.5 (-0.82, 1.83)	-0.27 (-2.34, 1.80)	0.23 (-1.52, 1.98)	-0.85 (-3.28, 1.57)	1.69 (-0.35, 3.73)
Estimated exposure to NO_2	90 days prior to conception	**-1.90 (-3.36, -0.44)**	**-2.86 (-4.08, -1.65)**	-0.72 (-2.43, 1.00)	**-2.83 (-4.27, -1.39)**	-0.31 (-2.22, 1.60)	**-1.72 (-3.31, -0.12)**
	First trimester	-0.08 (-1.57, 1.42)	-1.17 (-2.43, 0.08)	0.59 (-1.14, 2.32)	**-1.91 (-3.37, -0.46)**	1.28 (-0.68, 3.24)	**-1.80 (-3.46, -0.15)**
	Second trimester	**1.81 (0.41, 3.22)**	0.00 (-1.18, 1.18)	0.56 (-1.05, 2.17)	-0.75 (-2.11, 0.61)	-0.48 (-2.28, 1.33)	**-2.11 (-3.63, -0.60)**
	Third trimester	0.04 (-1.54, 1.62)	**-1.97 (-3.29, -0.65)**	0.51 (-1.45, 2.47)	**-1.92 (-3.57, -0.26)**	1.52 (-0.66, 3.69)	**-1.92 (-3.76, -0.09)**
	Total pregnancy	0.67 (-0.56, 1.89)	**-1.08 (-2.11, -0.05)**	0.41 (-0.83, 1.64)	**-1.15 (-2.19, -0.11)**	0.67 (-0.77, 2.12)	**-1.68 (-2.89, -0.46)**

All significant findings in the table are bold.

*Models adjusted for maternal age at enrolment, infant's sex, maternal BMI at 11–14 weeks' gestation, primiparity, monthly household income level, and season of births.

**Models adjusted for maternal age at enrolment, infant's sex, maternal BMI at 11–14 weeks' gestation, primiparity, monthly household income level, and season of births, and adjusted for the other air pollutant.

For peer review only

For peer review only - http://bmjopen.bmj.com/site/about/guidelines.xhtml

In the adjusted model, the risk of PDD was found to increase by 112% and 42% with each per-IQR increase in $PM_{2.5}$ (OR: 2.12, 95% CI: 1.45, 3.11) and NO_2 (OR: 1.42, 95% CI: 1.16, 1.75) in the 90 days prior to conception (**Table 4**). There was also a significant association between increased NO_2 exposure and the risk of PDD in T1 (OR: 1.29, 95% CI: 1.05, 1.58), T3 (OR: 1.27, 95% CI: 1.01, 1.60), and the whole pregnancy period (OR: 1.17, 95% CI: 1.02, 1.36). We did not observe any association with MDD in any pregnancy periods.

For peer review only

For peer review only - http://bmjopen.bmj.com/site/about/guidelines.xhtml

Table 4. Associations between $PM_{2.5}$ and NO_2 exposure in different pregnancy periods and mental and psychomotor developmental delay

Per IQR increase in		Crude models		Adjusted models*		Co-exposure models**	
		MDD (95% CI) (N=946)	PDD (95% CI) (N=946)	MDD (95% CI) (N=945)	PDD (95% CI) (N=945)	MDD (95% CI) (N=945)	PDD (95% CI) (N=945)
Estimated exposure to $PM_{2.5}$	90 days prior to conception	**1.45 (1.16, 1.83)**	**1.49 (1.20, 1.83)**	0.95 (0.64, 1.42)	**2.12 (1.45, 3.11)**	0.97 (0.63, 1.51)	**1.78 (1.17, 2.71)**
	First trimester	1.05 (0.83, 1.33)	1.04 (0.84, 1.28)	1.14 (0.73, 1.79)	1.42 (0.96, 2.11)	1.35 (0.80, 2.25)	1.16 (0.74, 1.82)
	Second trimester	**0.63 (0.49, 0.80)**	**0.77 (0.63, 0.95)**	0.81 (0.54, 1.22)	0.72 (0.51, 1.02)	0.83 (0.52, 1.31)	**0.57 (0.38, 0.85)**
	Third trimester	1.23 (0.96, 1.58)	1.19 (0.95, 1.49)	1.25 (0.82, 1.90)	1.17 (0.80, 1.70)	1.39 (0.87, 2.23)	0.98 (0.64, 1.49)
	Total pregnancy	0.84 (0.69, 1.03)	0.98 (0.82, 1.18)	1.07 (0.82, 1.39)	1.07 (0.84, 1.35)	1.17 (0.86, 1.59)	0.90 (0.68, 1.20)
Estimated exposure to NO_2	90 days prior to conception	1.20 (0.99, 1.45)	**1.41 (1.19, 1.67)**	0.97 (0.77, 1.21)	**1.42 (1.16, 1.75)**	0.97 (0.76, 1.25)	1.24 (0.99, 1.56)
	First trimester	0.97 (0.80, 1.17)	1.18 (0.99, 1.40)	0.91 (0.72, 1.13)	**1.29 (1.05, 1.58)**	0.84 (0.65, 1.09)	1.24 (0.99, 1.57)
	Second trimester	**0.79 (0.66, 0.95)**	1.04 (0.88, 1.22)	0.94 (0.76, 1.15)	1.14 (0.95, 1.38)	0.98 (0.77, 1.24)	**1.31 (1.06, 1.63)**
	Third trimester	1.04 (0.85, 1.28)	**1.25 (1.04, 1.50)**	0.94 (0.73, 1.21)	**1.27 (1.01, 1.60)**	0.86 (0.65, 1.14)	1.28 (0.99, 1.65)
	Total pregnancy	0.92 (0.79, 1.07)	**1.16 (1.01, 1.33)**	0.94 (0.80, 1.11)	**1.17 (1.02, 1.36)**	0.9 (0.75, 1.08)	**1.21 (1.02, 1.43)**

All significant findings in the table are bold.
*Models adjusted for maternal age at enrolment, infant's sex, maternal BMI at 11–14 weeks' gestation, primiparity, monthly household income level, and season of births.
**Models adjusted for maternal age at enrolment, infant's sex, maternal BMI at 11–14 weeks' gestation, primiparity, monthly household income level, and season of births, and adjusted for the other air pollutant.

For peer review only

DISCUSSION

We analyzed associations between modelled $PM_{2.5}$ and NO_2 pre- and during pregnancy with birth and neurodevelopment outcomes in singleton children born in a south-western metropolis of China in 2015-16. We found the likelihood of SGA increased by 33% per IQR higher exposure to NO_2 in the whole pregnancy periods after adjusted for maternal age at enrolment, infant's sex, maternal BMI at 11–14 weeks' gestation, primiparity, monthly household income level, and season of births and $PM_{2.5}$. For childhood cognitive development, increased exposure to $PM_{2.5}$ and NO_2 in the 90 days prior to conception were both associated with lower PDI scores, with the effect size per IQR being higher for $PM_{2.5}$ than for NO_2. Increased NO_2 exposure was associated with an increased risk of PDD during different trimesters of pregnancy.

Many studies from other geographic areas, including Europe (36-38), the United States (22, 26), and Asia (23, 39-41), have found significant associations between prenatal air pollution exposure and a variety of adverse neurodevelopmental outcomes. Our finding of a negative association between prenatal NO_2 air pollution exposure and infant neurocognitive development is consistent with these reports. A recent Chinese birth cohort study of 15,778 child-mother pairs in Foshan reported that maternal NO_2 exposure during pregnancy was associated with an increased risk of suspected developmental delay (OR: 1.06, 95% CI: 0.94, 1.19) measured by a five-domain scale and developmental quotient (DQ) (23). A birth cohort study of 520 mother-child pairs in South Korea reported that maternal NO_2 exposure during pregnancy was associated with impairment of psychomotor development ($\beta = -1.30$, $p = 0.05$) but – as in the present study - not with cognitive function ($\beta = -0.84$, $p = 0.20$) (39). However, results from previous research varied by air pollutants. For example, a Chinese study of 1193 mother-newborn pairs in Changsha found significant associations between $PM_{2.5}$ exposure in trimester two and lower neurobehavioral developmental scores, while other air pollutants such as PM_{10}, carbon monoxide (CO), and Sulfur dioxide (SO_2) had null or even reverse associations. In this study, we observed that the negative effect of NO_2 exposure during pregnancy on PDI is significant at 5% level; this negative effect of NO_2 still remained after adjustment for $PM_{2.5}$. This heterogeneity may relate to the temporality of exposure assessment, types of outcome assessment instruments or evaluators, and the levels of air pollution. In addition, air pollution mixtures may have differed among the study regions, thus there are several potential explanations for the heterogeneity of the findings. We also observed negative correlations between certain exposures, indicating the need to consider potential collinearity in our two-pollutant models. In Chongqing, a major industrial city in southwest China, air pollution may

For peer review only - http://bmjopen.bmj.com/site/about/guidelines.xhtml

come from industrial and traffic emissions, construction activities, and dust, and negative correlations may occur if different sources contribute disproportionately to each pollutant. Their correlations may also be affected by seasonal changes and variations in weather patterns. Future research should also explore the impact of source-specific air pollution on children's cognitive health.

To date, most studies on prenatal air pollution exposure and child neurodevelopment have been conducted in developed countries with relatively low levels of air pollution. In this study, the level of air pollution was higher (median $PM_{2.5}$: 57.31 μg/m^3, IQR: 5.76; median NO_2: 50.46 μg/m^3, IQR: 5.51) compared to studies in developed countries such as Europe and the United States. In a multi-centre European cohort, the mean $PM_{2.5}$ and NO_2 exposure concentration during pregnancy were 13.4 μg/m^3 and 11.5 μg/m^3 (36). Researchers found that the psychomotor development score significantly decreased by 0.68 points (95% CI: -1.25, -0.11) for every 10 μg/m^3 increase in NO_2, and there was also a non-significant decrease of 1.64 points (95% CI: -3.47, 0.18) for every 5 μg/m^3 increase in $PM_{2.5}$ during pregnancy (36). Factors such as the types of pollutants and concentrations may differ between China and other regions with a lower air pollution level, leading to variations in the observed effects.

For peer review only

Contrary to expectations**,** we found significant positive associations between prenatal exposure to $PM_{2.5}$ air pollution in the second trimester and PDI. However, no association was observed between $PM_{2.5}$ exposures in the second trimester and the risk of PDD. Given the existing literature and the conflicted observation here, we believe that this is likely to be spurious/sample specific. Some plausible explanations include the uneven distribution of PDI scores, the potentially inappropriate selection of the cut-off value of 85 (which may not effectively discriminate between groups), or the possibility that the observed outcome occurred by chance. Several epidemiological studies have reported associations between prenatal exposure to high levels of $PM_{2.5}$ and lower neurodevelopment in children ranging in age from 6 months to 6 years (12, 37, 42-44). In agreement with our findings, a multi-centre cohort study from six European countries investigated the effects of prenatal exposure to multiple air pollutants including $PM_{2.5}$, PM_{10}, coarse particles, NO_2 and nitrogen oxides (NOx) among 9482 children between 1 and 6 years; the authors found nonsignificant positive associations between prenatal $PM_{2.5}$ exposure and normal neurodevelopment (β: 1.64, 95% CI: −3.47, 0.18; per 5 μg/m^3 increase in $PM_{2.5}$) (36). Similarly, another study examining the effects of multiple pollutant exposures on early childhood cognition at 40 days of age in a highly exposed area of Spain also found PM_{10}, PM_{coarse}, $PM_{2.5absorbance}$, NO_2, NO_x, and Ozone (O_3) were linked to lower

For peer review only - http://bmjopen.bmj.com/site/about/guidelines.xhtml

motor function in children, except for $PM_{2.5}$ (45). The inconsistent findings could be because of heterogeneity between studies in terms of exposure (e.g., exposure assessment methods used, $PM_{2.5}$ exposure levels, or composition of $PM_{2.5}$).

The prevalence of MDD and PDD in our study is higher than in other studies that also used the CBSID to report developmental delay rates, which were at 17% (46), 15.78% (47), and 13.68% (48). This may be attributed to the younger age of infants in our study, which were assessed at around 12 months, compared to most studies assessed at around 24 months. A Chinese study and a South Korean study also found lower scores on the MDI and PDI in 1-year-old children (49, 50). Aside from the conflicting findings regarding prenatal $PM_{2.5}$ exposure and neurodevelopmental outcomes, results regarding the most potential sensitive time windows before and during pregnancy are also inconclusive. Some studies suggested that early-to-mid pregnancy might be a potential sensitive period (22, 23), while other studies found stronger associations for middle-to- late pregnancy, thus results are equivocal (20, 25, 26).

The potential biological mechanisms by which air pollution could affect neurodevelopment are not yet clearly understood. There is evidence suggesting that exposure to prenatal $PM_{2.5}$ could potentially induce maternal immune activation during pregnancy (51). Higher levels of cytokines or reactive oxygen species may potentially interfere fetal neurodevelopment through three mechanisms: crossing the placental barrier into the fetal body, inducing fetal immune dysregulation, and contributing to inadequate placental perfusion that affects nutritional processes and oxygenation of maternal blood (52). More research is needed to investigate trimester effects of air pollution on neurodevelopment and provide better understanding on the underlying biological mechanisms. Our study is the first to consider an exposure window 90 days prior to conception for NO_2. A novel observation is that effects of NO_2 or $PM_{2.5}$ air pollution on child cognition can be seen at least 90 days prior to conception, representing a potentially vulnerable periods in relation to air pollution on neurodevelopment. Similar results were found in previous study recruited 1329 mother-child pairs in Wuhan, China (12). This study reported a higher level of $PM_{2.5}$ during preconception (Median: 76.1 μg/m^3) and in the first trimester (Median: 82.3 μg/m^3). This study found for each doubling of $PM_{2.5}$ exposure during preconception, children's PDI scores was reduced by 6.15 (95% CI: -8.84, -3.46) points. A potential explanation is that preconception air pollution exposures induce genetic and epigenetic alterations in sperm, that increase the risk of adverse health outcomes in offspring (53, 54). To date, all studies examined the effect of maternal preconception exposure while

For peer review only - http://bmjopen.bmj.com/site/about/guidelines.xhtml

omitting paternal exposures (17). Future studies should consider the effect of preconception paternal exposure in relation to childhood health outcomes.

This study has several strengths. We developed an LUR model to capture spatial and temporal variations of air pollution at individual level to reduce exposure misclassification if using monitoring stations. This is a novel study to investigate both pre-conception and prenatal $PM_{2.5}$ and NO_2 exposure with neurodevelopment outcomes among young infants, in the context of a relatively high air pollution urban environment. The exposure levels in our study were similar as those in comparable urban areas in Chinese cities. A study in Shanghai, China reported an average NO_2 exposure during pregnancy from 2014 to 2015, predicted by the LUR model, of 48.23 μg/m^3 (Mean $PM_{2.5}$ in our study: 50.52 μg/m^3) (55). Similarly, a study in Tianjin found the annual average $PM_{2.5}$ exposure to be 62 μg/m^3 in 2017 (Mean NO_2 in our study: 57.48 μg/m^3) (56). Wu et al. developed a LUR model for $PM_{2.5}$ in the main urban area of Chongqing (57). This model predicted an annual average $PM_{2.5}$ concentration of 40.6 μg/m^3 (57), whereas our prediction is higher at 55.9 μg/m^3 (19). This difference can be attributed to the temporal variations. Wu et al. used monitoring data from 2013, while we utilized data from 2015. It could be considered that our GAM model, with its temporal component, could explain temporal variations and is more suitable for pregnancy-specific exposure estimates.

A major limitation of this study was that our sample size was relatively small, limiting the statistical power to assess several outcomes, although the higher exposures in Chongqing than in some other studies may increase probability of detecting effects. In terms of limitations, due to a lack of information on participant time-activity patterns, exposure estimates in this study refer only to ambient concentrations at home addresses, and no other activity spaces (e.g., indoor, workplace, commuting) were considered. We may have thus underestimated total air pollution exposure. Second, we defined exposure windows for clinically-defined trimesters; sensitive periods may be shorter or longer than 3 months, or they may exist in the overlap of multiple trimesters. However, we were unable to investigate the sensitive time windows using established methods such as distributed lag non-linear models due to the lack of highly time-resolved air pollution estimates. Third, the performance of the NO_2 spatiotemporal model was low (COR-R^2: 0.39), which may introduce exposure misclassification and therefore bias in the coefficients. It may lead to underestimation of the association if the NO_2 spatiotemporal model inadequately represents the true variability in NO_2 levels. Or conversely, it could overestimate the association between NO_2 exposure and the outcome if the model fails to account for certain factors or inaccurately estimates NO_2 levels. Finally, we were unable to include some other air

For peer review only - http://bmjopen.bmj.com/site/about/guidelines.xhtml

pollutants such as polycyclic aromatic hydrocarbons (PAH), black carbon (BC) and Ozone, which have bene found particular harmful to neurodevelopment in children (58). Although we have accounted for most of the important confounders in this study, unfortunately, we did not collect information on the feeding patterns of infants. This may undermine the validity and reliability of our findings.

CONCLUSION

This study provides evidence for an association between NO_2 exposure prior to- and during pregnancy with birth and neurodevelopmental outcomes in a birth cohort in Chongqing, China. Exposure to NO_2 and $PM_{2.5}$ exposure before pregnancy was associated with a lower psychomotor development score. Increased NO_2 exposure was linked to a risk of psychomotor development delay during various pregnancy trimesters.

Declarations

Ethics approval and consent to participate

Ethical approval for this study was granted by the Ethics Committee of Chongqing Medical University (#2014034). The participants provided their written informed consent to participate in this study. Written informed consent was obtained from the individual(s) for the publication of any potentially identifiable images or data included in this article.

Data availability statement

The data that support the findings of this study are available from Chongqing Medical University but restrictions apply to the availability of these data, which were used under license for the current study, and so are not publicly available. Data are however available from the authors upon reasonable request and with permission of Chongqing Medical University.

Competing interests

The authors declare that they have no conflicts of interests.

Funding

For peer review only

This work was supported by the National Natural Science Foundation of China (No. 81971406, 82271715), The 111 Project (Yuwaizhuan (2016)32), Chongqing Science & Technology Bureau (CSTB2022NSCQ-MSX1680), Youth Innovation Team Development Support Program of Chongqing Medical University (W0083), and Smart Medicine Research Project of Chongqing Medical University (No. ZHYX202103), Zunyi science and technology plan project (Zunshikehe HZ (2022)153). The research was supported by National Institute for Health Research (NIHR) Health Protection Research Unit in Environmental Exposures and Health, a partnership between UK Health Security Agency, the Health and Safety Executive and the University of Leicester and by the NIHR Leicester Biomedical Research Centre (BRC). The views expressed are those of the author(s) and not necessarily those of the NIHR, the Department of Health and Social Care or UK Health Security Agency.

Contributors

Y. X., T.L.H., H.Z. and PNB. conceived and designed research; T.Z., Y.X. and H.Z. recruited the patients and collected the samples; T.K., A.H., and J.G. constructed the air pollution model; Y.C. analyzed, interpreted the data and prepared the figures; Y.C and T.K were major contributors in writing the manuscript text; YSC, JC, TLH, YX, ALH, and PNB substantively revised the manuscript; All authors read and approved the final manuscript.

Acknowledgements

The authors would like to acknowledge the clinical research staff who recruited subjects and facilitated sample collection, the women and their families who participated in the CLIMB study, and Jamie de Seymour, the leading author for diet pattern analysis in the CLIMB cohort, for her help and advice during the analysis process.

References

1. Llop S, Ballester F, Estarlich M, Esplugues A, Rebagliato M, Iniguez C. Preterm birth and exposure to air pollutants during pregnancy. Environmental Research. 2010;110(8):778-85.

2. Ambient air pollution and low birthweight: a European cohort study (ESCAPE). The Lancet Respiratory Medicine. 2013;1(9):695-704.

3. Stieb DM, Chen L, Hystad P, Beckerman BS, Jerrett M, Tjepkema M, et al. A national study of the association between traffic-related air pollution and adverse pregnancy outcomes in Canada, 1999–2008. Environmental Research. 2016.

For peer review only

4. Barker DJ. The origins of the developmental origins theory. Journal of internal medicine. 2007;261(5):412-7.

5. Volk HE, Perera F, Braun JM, Kingsley SL, Gray K, Buckley J, et al. Prenatal air pollution exposure and neurodevelopment: A review and blueprint for a harmonized approach within ECHO. Environmental research. 2021;196:110320.

6. Feng S, Dan G, Liao F, Zhou F, Wang X. The health effects of ambient PM2.5 and potential mechanisms. Ecotoxicology and Environmental Safety. 2016;128:67-74.

7. Massa NR, Guangyun M, Xingyou Z, Xiumei H, Zhu C, Sampankanpanich SC, et al. Intrauterine Inflammation and Maternal Exposure to Ambient PM2.5 during Preconception and Specific Periods of Pregnancy: The Boston Birth Cohort. Environ Health Perspect. 2016;124(10):1608-15.

8. Vecoli C, Montano L, Andreassi MG. Environmental pollutants: genetic damage and epigenetic changes in male germ cells. Environmental Science and Pollution Research. 2016;23:23339-48.

9. Marcho C, Oluwayiose OA, Pilsner JR. The preconception environment and sperm epigenetics. Andrology. 2020;8(4):924-42.

10. Udagawa O, Furuyama A, Imai K, Fujitani Y, Hirano S. Effects of diesel exhaust-derived secondary organic aerosol (SOA) on oocytes: Potential risks to meiotic maturation. Reproductive Toxicology. 2018;75:56-64.

11. Zhang J-Y, Wu Q-J, Huang Y-H, Li J, Liu S, Chen Y-L, et al. Association between maternal exposure to ambient PM10 and neural tube defects: a case-control study in Liaoning Province, China. International Journal of Hygiene and Environmental Health. 2020;225:113453.

12. Li J, Liao J, Hu C, Bao S, Mahai G, Cao Z, et al. Preconceptional and the first trimester exposure to PM2. 5 and offspring neurodevelopment at 24 months of age: Examining mediation by maternal thyroid hormones in a birth cohort study. Environmental Pollution. 2021;284:117133.

13. Jo H, Eckel SP, Chen J-C, Cockburn M, Martinez MP, Chow T, et al. Gestational diabetes mellitus, prenatal air pollution exposure, and autism spectrum disorder. Environment international. 2019;133:105110.

14. Raz R, Roberts AL, Lyall K, Hart JE, Just AC, Laden F, et al. Autism spectrum disorder and particulate matter air pollution before, during, and after pregnancy: a nested case–control analysis within the Nurses' Health Study II cohort. Environmental health perspectives. 2015;123(3):264-70.

For peer review only

For peer review only - http://bmjopen.bmj.com/site/about/guidelines.xhtml

15. Grineski S, Alexander C, Renteria R, Collins TW, Bilder D, VanDerslice J, et al. Trimester-specific ambient PM2.5 exposures and risk of intellectual disability in Utah. Environmental Research. 2023;218:115009.

16. Shang L, Yang L, Yang W, Huang L, Qi C, Yang Z, et al. Effects of prenatal exposure to NO(2) on children's neurodevelopment: a systematic review and meta-analysis. Environ Sci Pollut Res Int. 2020;27(20):24786-98.

17. Blanc N, Liao J, Gilliland F, Zhang JJ, Berhane K, Huang G, et al. A systematic review of evidence for maternal preconception exposure to outdoor air pollution on Children's health. Environmental Pollution. 2022:120850.

18. Huang S, Mo T-T, Norris T, Sun S, Zhang T, Han T-L, et al. The CLIMB (Complex Lipids In Mothers and Babies) study: protocol for a multicentre, three-group, parallel randomised controlled trial to investigate the effect of supplementation of complex lipids in pregnancy, on maternal ganglioside status and subsequent cognitive outcomes in the offspring. BMJ open. 2017;7(10):e016637.

19. Harper A, Baker PN, Xia Y, Kuang T, Zhang H, Chen Y, et al. Development of spatiotemporal land use regression models for PM2. 5 and NO2 in Chongqing, China, and exposure assessment for the CLIMB study. Atmospheric Pollution Research. 2021;12(7):101096.

20. Chen B, Huang S, He J, He Q, Chen S, Liu X, et al. Sex-specific influence of prenatal air pollutant exposure on neonatal neurobehavioral development and the sensitive window. Chemosphere. 2020;254:126824.

21. Volk HE, Perera F, Braun JM, Kingsley SL, Gray K, Buckley J, et al. Prenatal air pollution exposure and neurodevelopment: A review and blueprint for a harmonized approach within ECHO. Environ Res. 2021;196:110320.

22. Ha S, Yeung E, Bell E, Insaf T, Ghassabian A, Bell G, et al. Prenatal and early life exposures to ambient air pollution and development. Environmental research. 2019;174:170-5.

23. Su X, Zhang S, Lin Q, Wu Y, Yang Y, Yu H, et al. Prenatal exposure to air pollution and neurodevelopmental delay in children: A birth cohort study in Foshan, China. Science of The Total Environment. 2022;816:151658.

24. Bennet L, Walker DW, Horne RS. Waking up too early–the consequences of preterm birth on sleep development. The Journal of physiology. 2018;596(23):5687-708.

For peer review only

For peer review only - http://bmjopen.bmj.com/site/about/guidelines.xhtml

25. Wang P, Zhao Y, Li J, Zhou Y, Luo R, Meng X, et al. Prenatal exposure to ambient fine particulate matter and early childhood neurodevelopment: A population-based birth cohort study. Science of The Total Environment. 2021;785:147334.

26. Chiu Y-HM, Hsu H-HL, Coull BA, Bellinger DC, Kloog I, Schwartz J, et al. Prenatal particulate air pollution and neurodevelopment in urban children: examining sensitive windows and sex-specific associations. Environment international. 2016;87:56-65.

27. Huang S, Mo T-T, Norris T, Sun S, Zhang T, Han T-L, et al. The CLIMB (Complex Lipids In Mothers and Babies) study: protocol for a multicentre, three-group, parallel randomised controlled trial to investigate the effect of supplementation of complex lipids in pregnancy, on maternal ganglioside status and subsequent cognitive outcomes in the offspring. BMJ Open. 2017;7(10):e016637.

28. Liu Y, Zhong M, Sun Q, Zhong B, Luo K. Temporal and spatial variations of atmospheric pollutants in Chongqing metropolitan area during autumn. Huanjing Kexue Xuebao/Acta Scientiae Circumstantiae. 2016;36:2344-54.

29. Statistics CMBo. Chongqing Statistical Yearbook 2015. 2015.

30. Jiaojiao L, Si C, Jie Z, Di Y. Characteristic Analysis of PM2. 5 and Evaluation of Forecast Results in Chongqing. Environmental Science & Technology (10036504). 2020;43(6).

31. Albert BB, Derraik JG, Xia Y-Y, Norris T, Zhang T, Han T-L, et al. Supplementation with milk enriched with complex lipids during pregnancy: a double-blind randomized controlled trial. Plos one. 2021;16(2):e0244916.

32. Zhao X, Xia Y, Zhang H, Baker PN, Norris T. Birth weight charts for a Chinese population: an observational study of routine newborn weight data from Chongqing. BMC Pediatr. 2019;19(1):426.

33. Shourong Y, Xuerong L, Zhiwei Y. The revising of the bayley scales of infant development (BSID) in China. Chin J Clin Psychol. 1993;2:71-5.

34. Chen Y-T, Zhang T, Chen C, Xia Y-Y, Han T-L, Chen X-Y, et al. Associations of early pregnancy BMI with adverse pregnancy outcomes and infant neurocognitive development. Scientific Reports. 2021;11(1):1-8.

35. Çelik P, Sucakli IA, Yakut HI. Which Bayley-III cut-off values should be used in different developmental levels? Turkish journal of medical sciences. 2020;50(4):764-70.

36. Guxens M, Garcia-Esteban R, Giorgis-Allemand L, Forns J, Badaloni C, Ballester F, et al. Air pollution during pregnancy and childhood cognitive and psychomotor development: six European birth cohorts. Epidemiology. 2014:636-47.

For peer review only - http://bmjopen.bmj.com/site/about/guidelines.xhtml

37. Lertxundi A, Baccini M, Lertxundi N, Fano E, Aranbarri A, Martínez MD, et al. Exposure to fine particle matter, nitrogen dioxide and benzene during pregnancy and cognitive and psychomotor developments in children at 15 months of age. Environment international. 2015;80:33-40.

38. Porta D, Narduzzi S, Badaloni C, Bucci S, Cesaroni G, Colelli V, et al. Air pollution and cognitive development at age 7 in a prospective Italian birth cohort. Epidemiology. 2016;27(2):228-36.

39. Kim E, Park H, Hong Y-C, Ha M, Kim Y, Kim B-N, et al. Prenatal exposure to PM10 and NO2 and children's neurodevelopment from birth to 24 months of age: Mothers and Children's Environmental Health (MOCEH) study. Science of the Total Environment. 2014;481:439-45.

40. Lin C-C, Yang S-K, Lin K-C, Ho W-C, Hsieh W-S, Shu B-C, et al. Multilevel analysis of air pollution and early childhood neurobehavioral development. International journal of environmental research and public health. 2014;11(7):6827-41.

41. Yorifuji T, Kashima S, Diez MH, Kado Y, Sanada S, Doi H. Prenatal exposure to traffic-related air pollution and child behavioral development milestone delays in Japan. Epidemiology. 2016;27(1):57-65.

42. Tozzi V, Lertxundi A, Ibarluzea JM, Baccini M. Causal effects of prenatal exposure to PM2. 5 on child development and the role of unobserved confounding. International Journal of Environmental Research and Public Health. 2019;16(22):4381.

43. Hurtado-Díaz M, Riojas-Rodríguez H, Rothenberg SJ, Schnaas-Arrieta L, Kloog I, Just A, et al. Prenatal PM2. 5 exposure and neurodevelopment at 2 years of age in a birth cohort from Mexico city. International journal of hygiene and environmental health. 2021;233:113695.

44. Lertxundi A, Andiarena A, Martínez MD, Ayerdi M, Murcia M, Estarlich M, et al. Prenatal exposure to PM2.5 and NO2 and sex-dependent infant cognitive and motor development. Environmental Research. 2019;174:114-21.

45. Iglesias-Vázquez L, Binter A-C, Canals J, Hernández-Martínez C, Voltas N, Ambros A, et al. Maternal exposure to air pollution during pregnancy and child's cognitive, language, and motor function: ECLIPSES study. Environmental Research. 2022;212:113501.

46. Wang H, Zhang H, Li J, Liao J, Liu J, Hu C, et al. Prenatal and early postnatal exposure to ambient particulate matter and early childhood neurodevelopment: A birth cohort study. Environmental Research. 2022;210:112946.

47. Zhu Y, Li X, Chen J, Gong W. Perinatal depression trajectories and child development at one year: a study in China. BMC pregnancy and childbirth. 2024;24(1):176.

For peer review only - http://bmjopen.bmj.com/site/about/guidelines.xhtml

48. Wang A, Qi W, Liu H, Wan Y, Xu S, Xia W. Association of maternal exposure to chlorpyrifos during pregnancy with neurodevelopmental abnormality in two-year-old children: a survey in Wuhan city. Chinese Journal of Public Health. 2023;39(12):1590-5.

49. 谢松敏, 王鲜艳, 姚英民. 贝利婴幼儿发展量表在婴幼儿保健中的应用. 护理学报. 2006;13(4):76-7.

50. Kim E, Park H, Hong Y-C, Ha M, Kim Y, Kim B-N, et al. Prenatal exposure to PM10 and NO2 and children's neurodevelopment from birth to 24months of age: Mothers and Children's Environmental Health (MOCEH) study. Science of The Total Environment. 2014;481:439-45.

51. Umezawa M, Onoda A, Korshunova I, Jensen AC, Koponen IK, Jensen KA, et al. Maternal inhalation of carbon black nanoparticles induces neurodevelopmental changes in mouse offspring. Particle and Fibre Toxicology. 2018;15:1-18.

52. Monk C, Lugo-Candelas C, Trumpff C. Prenatal developmental origins of future psychopathology: mechanisms and pathways. Annual review of clinical psychology. 2019;15:317-44.

53. Xu R, Zhong Y, Li R, Li Y, Zhong Z, Liu T, et al. Association between exposure to ambient air pollution and semen quality: A systematic review and meta-analysis. Science of The Total Environment. 2023;870:161892.

54. Braun JM, Messerlian C, Hauser R. Fathers matter: why it's time to consider the impact of paternal environmental exposures on children's health. Current epidemiology reports. 2017;4:46-55.

55. Ji X, Meng X, Liu C, Chen R, Ge Y, Kan L, et al. Nitrogen dioxide air pollution and preterm birth in Shanghai, China. Environmental research. 2019;169:79-85.

56. Zhang Y, Wang J, Chen L, Yang H, Zhang B, Wang Q, et al. Ambient PM2. 5 and clinically recognized early pregnancy loss: A case-control study with spatiotemporal exposure predictions. Environment International. 2019;126:422-9.

57. Wu J-S, Liao X, Peng J, Huang X-L. Simulation and influencing factors of spatial distribution of PM2. 5 concentrations in Chongqing. Huan Jing ke Xue= Huanjing Kexue. 2015;36(3):759-67.

58. Lopuszanska U, Samardakiewicz M. The relationship between air pollution and cognitive functions in children and adolescents: a systematic review. Cognitive and Behavioral Neurology. 2020;33(3):157-78.

For peer review only

FIGURES

Figure 1. Flow chart of the study population in CLIMB

Figure 2. Study area and location of monitoring sites (OpenStreetMap contributors, 2015; https://data.nextgis.com/en/region/CN-50/)

For peer review only

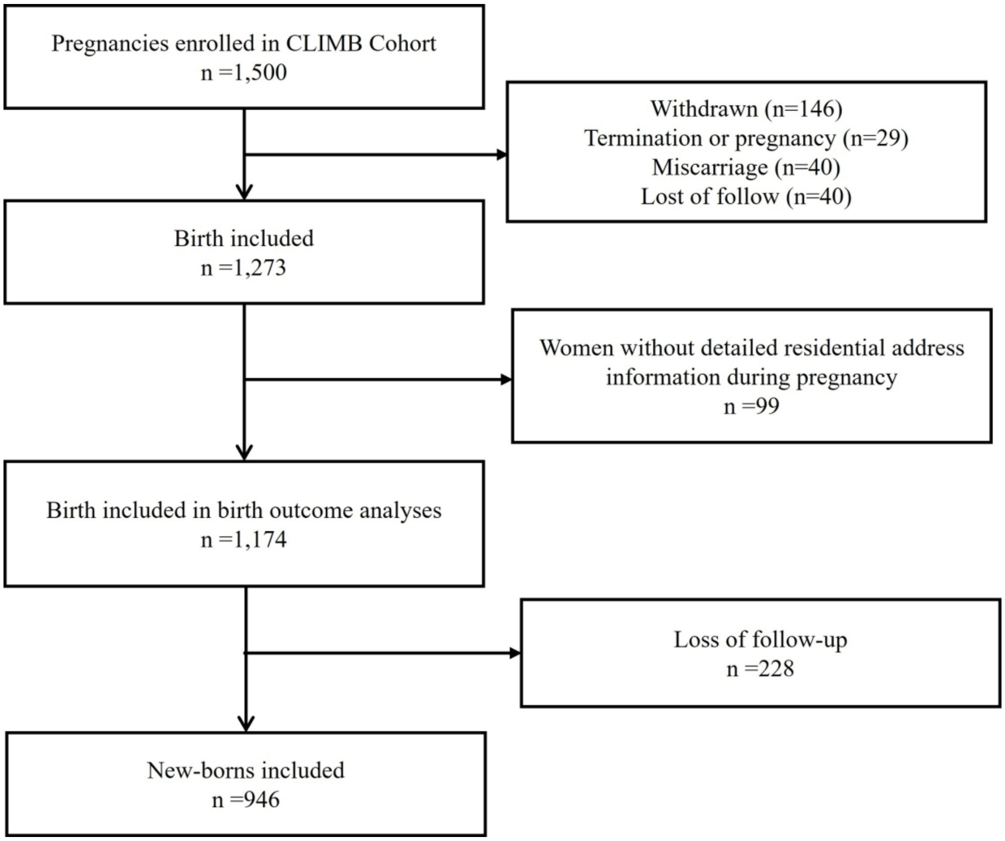

Figure 1 Flow chart of the study population in CLIMB

173x144mm (300 x 300 DPI)

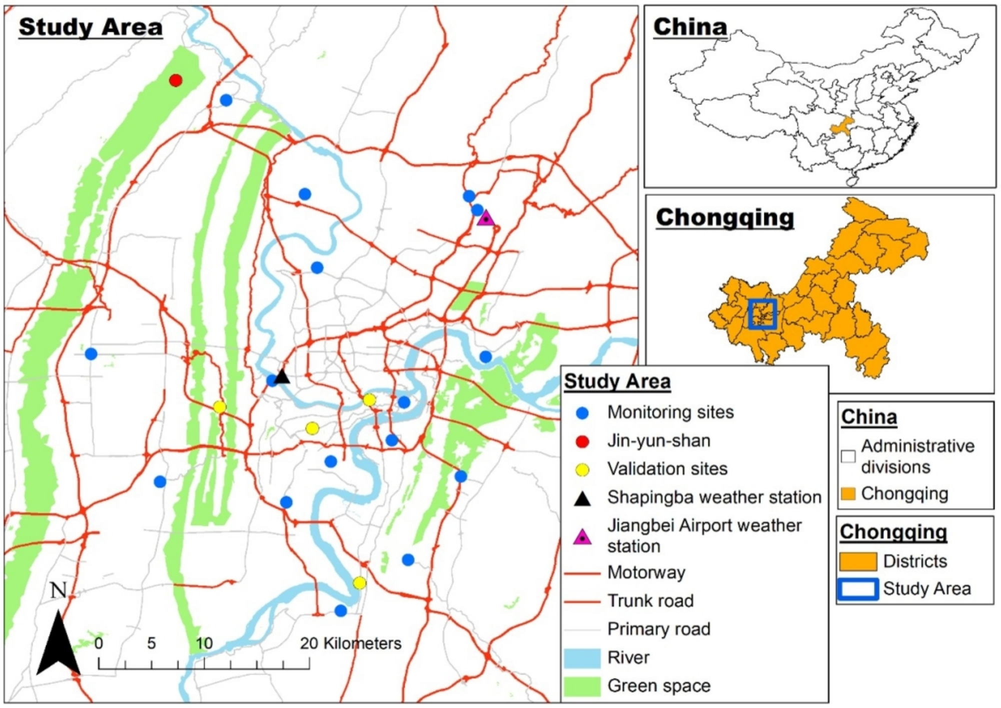

Figure 2 Study area and location of monitoring sites (OpenStreetMap contributors, 2015; https://data.nextgis.com/en/region/CN-50/).

159x112mm (220 x 220 DPI)

Associations of air pollution exposures in preconception and pregnancy with birth outcomes and infant neurocognitive development: analysis of the Complex Lipids in Mothers and Babies (CLIMB) prospective cohort in Chongqing, China

Yingxin Chen[1,2], Tao Kuang[3], Ting Zhang[4], Yutong Samuel Cai[1,2,5], John Colombo[6], Alex Harper[7], Ting-Li Han[8], Yinyin Xia[9], John Gulliver[10], Anna L Hansell[1,2,5], Hua Zhang[8], Philip N Baker[7,8]

1: Centre for Environmental Health and Sustainability, University of Leicester, Leicester, UK

2: The National Institute of Health Research (NIHR) Health Protection Research Unit (HPRU) in Environmental Exposure and Health at the University of Leicester, Leicester, UK

3: Department of public health and management, Zunyi Medical and Pharmaceutical College, Zunyi, 563000, Guizhou, China

4. Stomatological Hospital of Chongqing Medical University, Chongqing, 401147, China

5: NIHR Leicester Biomedical Research Centre, Leicester General Hospital, Leicester, UK

6: Schiefelbusch Institute for Life Span Studies and Department of Psychology, University of Kansas, Lawrence, KS 66045, USA

7: College of Life Sciences, University of Leicester, Leicester, UK

8: Department of Obstetrics and Gynaecology, the First Affiliated Hospital of Chongqing Medical University, Chongqing, 400016, China

9: School of Public Health, Chongqing Medical University, Chongqing, 400016, China

10: Environmental and Exposure Sciences, Population Health Research Institute, St George's, University of London, London, UK

Correspondence to: Yinyin Xia. School of Public Health, Chongqing Medical University, Chongqing, 400016, China. Email: 100118@cqmu.edu.cn

For peer review only

Supplement

eTable 1 Mental Development Index (Chinese version)

智力量表

（※可偶尔观察到）

序号	月龄	条目	计分
1	0.1	对铃声反应	
2	0.1	抱起时安静	
3	0.1	对摇鼓声反应	
4	0.1	对尖声反应：（电灯开关）	
5	0.1	短暂地注视红环	
6	0.2	短暂地注视人	
7	0.4	稍长时间地注视红环	
8	0.5	眼的水平协调活动（红环）	
9	0.7	眼的水平向天活动（光）	
10	0.7	眼睛追随移动的人	
11	0.7	对说话声反应	
12	0.8	眼的垂直协调活动（光）	
13	0.9	发声一至两次	
14	1	眼的垂直协调活动（红环）	
15	1.2	眼的旋转协调活动（光）	
16	1.2	眼的旋转细条活动（光环）	
17	1.3	※自由环视周围	
18	1.5	社交笑：测试者谈话与微笑时	
19	1.6	眼转向红环	
20	1.6	眼转向光	
21	1.6	※发声至少四次	
22	1.7	期待性兴奋	
23	1.7	对面部的纸有反应	
24	1.9	能用视觉辨认母亲	
25	1.9	社交笑：测试者微笑与安静时	
26	2	※对测试者的微笑和说话有发声反应	
27	2.1	※用眼睛寻找声源（详细说明）	
28	2.2	※发出两种不同的声音	
29	2.2	对手的遮蔽眨眼	
30	2.2	对面孔的消失有反应	
31	2.4	注视方木	
32	2.6	从一物转看另一物	
33	2.6	眼睛追随铅笔	
34	2.7	对抱起有预感性的调节反应	
35	2.9	目光追随横过桌面的球	
36	2.9	头追随悬摆的环	

For peer review only

37	3.1	头追随逐渐消失的勺子	
38	3.2	操作红环	
39	3.3	简单地玩摇鼓	
40	3.4	※轻轻地抚摸桌沿	
41	3.4	※意识到陌生环境	
42	3.5	头转向铃声	
43	3.6	头转向摇鼓声	
44	3.6	※手碰手的玩耍	
45	3.6	将红环送入口中	
46	3.7	伸手够悬环	
47	3.8	看自己的手	
48	4.2	接近悬环（优势手）	
49	4.4	※发声时的姿态（描述）	
50	4.4	※主动抚摸桌沿	
51	4.4	接近镜像	
52	4.4	注意小糖丸	
53	4.6	伸手取方木	
54	4.7	喜欢嬉戏	
55	4.9	伸手时眼手协调	
56	4.9	拾起方木（优势手）	
57	5	保持两块方木	
58	5	持久地看红环	
59	5	头部跟着掉下的勺转动	
60	5	探索性地玩纸	
61	5	对镜像微笑	
62	5	坚持够东西	
63	5.1	在小床内重新找到摇鼓	
64	5.1	※辨别生人	
65	5.4	举起倒扣的茶杯	
66	5.5	※敲打玩耍	
67	5.5	探索性地玩细绳	
68	5.5	伸手取第二块方木	
69	5.6	※由一手向另一手传递物体	
70	5.8	※对产生响声感兴趣	
71	5.9	灵巧而直接地拾起方木	
72	6	※对镜像开玩笑	
73	6	用把柄举起茶杯	
74	6	寻找掉落的勺子	
75	6.1	牵拉细绳获取红环	
76	6.1	保留三块方木中的两块	
77	6.6	※发出四个不同的音节	
78	6.8	能配合玩游戏	
79	7	恰当地牵拉细绳获取红环	
80	7.1	玩摇铃，对细节感兴趣	
81	7.4	企图获得三块方木	

For peer review only

82	7.4	有目的地摇铃	
83	7.5	※选择性地倾听熟悉的词语	
84	8	※对 da-da 或类同词	
85	8.1	暴露玩具	
86	8.2	注意测试者的乱写	
87	8.3	将手指插入桩板洞中	
88	8.6	观看书中图画	
89	8.9	对他人的言语要求有反应	
90	9.1	拿起茶杯获得方木	
91	9.8	寻找盒子里面的东西	
92	10.3	遵照命令将方木放入茶杯（放入数）	
93	10.7	企图模仿乱写	
94	10.8	模仿用勺子搅拌	
95	10.9	遵照命令停止	
96	10.9	推动小汽车	
97	11	模仿地拍打哨娃	
98	11.1	※重复引入发笑的把戏	
99	11.2	解开裹着的方木	
100	11.2	将三块方木放入杯中	
101	11.4	※快速而不清的表达	
102	11.4	揭开兰盒子的盖	
103	11.5	翻开书页	
104	11.5	摇晃悬环的	
105	11.8	将骰子放入盒中（6 个）	
106	12	恰当地握持画笔	
107	12.2	模仿说单词（记录用过的词）	
108	12.4	重复地插一根桩钉	
109	12.5	用手势表达想要的东西	
110	12.9	自动乱写	
111	12.9	能说两个词	
112	13	搭两层塔	
113	13.1	出示鞋子或其他衣服或自己的玩具	
114	13.2	从瓶中移出小糖丸	
115	13.3	掺九块方木放入杯中	
116	14.3	※盖上圆盒	
117	14.4	兰色模板：放置一个圆形模块	
118	14.8	用棍子够取玩具	
119	15.4	搭三层塔	
120	15.7	在 70 秒钟内插完桩钉	
121	16.1	指出娃娃身体的各部分：三个部位以上	
122	16.3	粉红模板：放置圆形模块	
123	16.6	兰色模板：放置两个圆形模块	
124	17.2	用笔模仿画一划	
125	17.5	在 42 秒钟内插完桩钉	
126	17.6	说出一物名	

For peer review only

127	17.7	对娃娃执行指令（在通过部位打钩：椅、杯、鼻）	
128	18.1	用语言表达要求	
129	18.6	不用于一划的乱写？	
130	18.8	兰色模板：放置两个圆快和方块	
131	18.8	指出三幅画	
132	19.1	能说两个单词的句子	
133	19.2	说出一副画名	
134	19.2	说出两幅画名	
135	19.3	找出两物	
136	19.8	在 30 秒钟内插完桩钉	
137	20.4	粉红模板：完成	
138	20.4	搭六层塔	
139	20.5	兰色模板，放置六个模块	
140	21	指出五副画	
141	21.1	说出三物名	
142	21.2	勉强合格地安装破娃娃	
143	21.2	区别两物：杯、盘、盒	
144	22.8	辨认钟表：第四张图 1，2，3，4，5	
145	22.9	说出三幅画名	
146	23.8	粉红模板（反转）	
147	24.3	近似地安装破娃娃	
148	24.6	区别三物：杯、盘、盒	
149	24.7	兰色模板，在 150 秒钟内完成	
150	25	搭八层塔	
151	25.1	指出七副画	
152	25.1	用方木搭火车	
153	25.7	说出五副画名	
154	26.3	模仿笔划：垂直线和水平线	
155	27.1	辨认钟表：第 2 张图	
156	27.6	理解两个方位词	
157	28	在 22 秒钟内插完桩钉	
158	28.5	兰色模板：90 秒钟内完成	
159	29.5	折纸	
160	29.6	兰色模板：60 秒钟内完成	
161	30+	正确安装破娃娃	
162	30+	“—”的概念	
163	30+	理解三个方位词	

For peer review only

eTable 2 Psychomotor Development Index (Chinese version)

运动量表

（※可偶尔观察到，△可在施测智力量表时观察到）

序号	月龄	条目	计分
1	0.1	抱起靠肩时抬头	
2	0.1	抱起靠肩时调整姿势	
3	0.1	侧头	
4	0.1	爬起	
5	0.8	△保留红环	
6	0.8	※伸臂玩耍	
7	0.8	※踢腿玩耍	
8	0.8	头起竖起：垂直位	
9	1.6	头部稳定地竖起	
10	1.7	抬头（背悬位）	
11	1.8	由侧卧转向仰卧	
12	2.2	在俯卧位时用双臂撑起自己	
13	2.2	支撑下坐起	
14	2.5	保持头部稳定	
15	2.6	※双手张开占优势	
16	3.3	头平衡	
17	3.4	※尺侧一手掌抓握方木	
18	3.5	轻度支撑坐位	
19	4.3	※由仰卧转向侧卧	
20	4.7	努力想坐起	
21	5.0	部分的拇指相对（桡侧一手掌）拾起方木	
22	5.1	独坐片刻	
23	5.1	※单手抽取	
24	5.2	※转腕	
25	5.2	牵拉坐起	
26	5.6	△试图获取小糖丸	
27	5.7	独立 30 秒钟或以上	
28	5.8	由仰卧转向俯卧	
29	6.2	稳定地独坐	
30	6.5	独坐时协调好	
31	6.6	※舀起小糖丸	
32	6.6	△完全的拇指相对拾起方木	
33	7	早期跨步运动	
34	7.5	牵拉站起	
35	7.6	※不完全的拇指相对抓糖丸	
36	7.6	走路之前的行进方式（俯卧、手膝、手足、其他）	
37	8.3	使两个勺子或方木在中线相碰	
38	8.5	跨步运动	

For peer review only

39	8.6	自己坐起	
40	8.6	借助家具站起	
41	8.9	精细地抓糖丸（灵巧地钳夹）	
42	9.6	拍手（中线技巧）	
43	9.8	坐下	
44	10	扶助下行走	
45	11.1	独站	
46	12	投球	
47	12.1	独走	
48	12.4	起立 I	
49	13.2	扶助下右足独站	
50	13.7	扶助下左足独站	
51	14.1	侧身走	
52	14.5	扶助上楼梯	
53	14.7	倒退走	
54	15.1	扶助下楼梯	
55	17.6	试图站在行木上	
56	18.7	左足独站	
57	19.3	单足踏在行木上走	
58	19.9	起立 II	
59	20.1	右足独站	
60	21.1	走直线：大致方向	
61	23.1	行木：双足站立	
62	24	踮脚走几步	
63	24.3	独自上楼梯：双足	
64	24.4	双足跳离地面	
65	25.3	独自下楼梯	
66	25.6	行木：企图跨步	
67	25.6	倒行两米半	
68	25.7	自第一级台阶下跳下	
69	29.2	自第二级台阶下跳下	
70	29.8	踮脚走两米半	
71	29.9	跳远：10 至 35cm（记录距离）	
72	30+	起立：III	
73	30+	上楼梯：双足交替向前	
74	30+	行木：交替步伐走部分路程	
75	30+	保持双足走在直线上（两米半）	
76	30+	跳远：35cm 至 60cm	
77	30+	跳过：5cm 高的绳子	
78	30+	跳远：60cm 至 85cm	
79	30+	独脚跳两次以上	
80	30+	下楼梯：双足交替向前	
81	30+	跳过 20cm 高的绳子	

For peer review only

For peer review only

eTable 3 Distributions of $PM_{2.5}$ and NO_2 exposure level in 90 days prior to conception, each trimester (T1, T2, and T3) and combined across whole pregnancy period (WP) (n = 1,174)

	Estimated exposure (μg/m³)						
	N	Minimum	25th percentile	Mean ± SD	Median ± IQR	75th percentile	Maximum
Estimated exposure to $PM_{2.5}$							
90 days prior to conception	1,174	38.17	44.00	52.91 ± 10.99	48.43 ± 18.07	62.06	80.53
First trimester	1,174	37.26	43.77	52.07 ± 10.98	47.26 ± 17.31	61.08	82.41
Second trimester	1,174	38.46	47.57	58.64 ± 12.21	57.97 ± 19.62	67.19	90.02
Third trimester	1,174	37.03	47.25	61.83 ± 16.04	58.82 ± 28.7	75.95	96.48
Total pregnancy	1,174	46.69	54.85	57.48 ± 3.97	57.31 ± 5.76	60.61	66.98
Estimated exposure to NO_2							
90 days prior to conception	1,174	25.86	45.49	49.59 ± 6.34	49.94 ± 8.27	53.76	70.48
First trimester	1,174	20.81	44.60	48.8 ± 6.43	48.92 ± 8.51	53.10	69.31
Second trimester	1,174	28.93	47.18	50.98 ± 6.23	51.20 ± 7.72	54.90	70.42
Third trimester	1,174	20.57	47.20	51.79 ± 6.78	52.45 ± 9.47	56.67	75.12
Total pregnancy	1,174	27.50	47.89	50.52 ± 5.08	50.46 ± 5.51	53.40	67.53

For peer review only

eTable 4 Pearson's correlations of $PM_{2.5}$ and NO_2 between each of the five different pregnancy time periods (N = 1,174)

Estimated exposure to		$PM_{2.5}$					NO_2				
		90 days prior to conception	First trimester	Second trimester	Third trimester	Total pregnancy	90 days prior to conception	First trimester	Second trimester	Third trimester	Total pregnancy
**$PM_{2.5}$**	90 days prior to conception	1									
	First trimester	-0.065	1								
	Second trimester	-0.779	-0.2012	1							
	Third trimester	0.288	-0.7613	-0.1688	1						
	Total pregnancy	-0.534	-0.2709	0.6838	0.3858	1					
**NO_2**	90 days prior to conception	0.6383	0.0684	-0.4588	0.3714	0.0376	1				
	First trimester	0.1537	0.6352	-0.0159	-0.4927	-0.0633	0.5545	1			
	Second trimester	-0.431	0.0714	0.6269	-0.0133	0.7251	0.3345	0.5399	1		
	Third trimester	0.3027	-0.5213	0.0528	0.6817	0.4432	0.7149	0.2159	0.5145	1	
	Total pregnancy	0.0057	0.0781	0.2862	0.0737	0.4779	0.6786	0.7435	0.8755	0.7331	1

For peer review only

eTable 5 Associations between $PM_{2.5}$ and NO_2 exposure in different pregnancy periods and adverse birth outcomes (unadjusted models)

Per IQR increase in		Mean difference		Odd ratios			
		Birth weight, grams (95% CI) (N=941)	Birth length, cm (95% CI) (N=927)	PTB (case: 33) (95% CI) (N=945)	LBW (case: 30) (95% CI) (N=945)	LGA (case: 108) (95% CI) (N=945)	SGA (case: 84) (95% CI) (N=945)
Estimated exposure to $PM_{2.5}$	90 days prior to conception	9.28 (-31.26, 49.83)	-0.09 (-0.27, 0.09)	0.98 (0.56, 1.74)	1.35 (0.77, 2.36)	1.2 (0.87, 1.64)	0.98 (0.68, 1.41)
	First trimester	21.95 (-16.90, 60.80)	0.14 (-0.03, 0.31)	0.98 (0.57, 1.70)	1 (0.57, 1.77)	0.97 (0.71, 1.33)	0.78 (0.54, 1.13)
	Second trimester	-18.21 (-57.78, 21.37)	0.04 (-0.13, 0.21)	0.85 (0.48, 1.50)	0.61 (0.32, 1.15)	0.92 (0.67, 1.27)	1.33 (0.94, 1.89)
	Third trimester	-37.38 (-81.43, 6.68)	**-0.32 (-0.51, -0.13)**	1.35 (0.74, 2.47)	1.51 (0.80, 2.85)	1.08 (0.76, 1.54)	1.12 (0.76, 1.66)
	Total pregnancy	-20.02 (-55.69, 15.65)	-0.1 (-0.26, 0.05)	0.81 (0.49, 1.33)	0.69 (0.41, 1.16)	1 (0.75, 1.34)	1.2 (0.87, 1.66)
Estimated exposure to NO_2	90 days prior to conception	-13.23 (-45.50, 19.03)	-0.12 (-0.26, 0.02)	1.2 (0.76, 1.89)	1.62 (0.99, 2.65)	1.21 (0.93, 1.57)	1.24 (0.92, 1.66)
	First trimester	0.3 (-32.36, 32.96)	0.08 (-0.06, 0.22)	1.01 (0.64, 1.60)	1.15 (0.71, 1.86)	1.17 (0.90, 1.52)	1.27 (0.94, 1.71)
	Second trimester	-22.85 (-53.39, 7.70)	-0.04 (-0.17, 0.09)	1.11 (0.72, 1.70)	1.08 (0.69, 1.69)	1.06 (0.83, 1.36)	**1.46 (1.10, 1.93)**
	Third trimester	-32.72 (-67.16, 1.72)	**-0.16 (-0.32, -0.01)**	1.13 (0.69, 1.85)	1.35 (0.80, 2.28)	1.24 (0.94, 1.65)	**1.58 (1.14, 2.18)**
	Total pregnancy	-16.58 (-43.35, 10.20)	-0.03 (-0.15, 0.09)	1.03 (0.71, 1.50)	1.13 (0.76, 1.69)	1.16 (0.93, 1.44)	**1.44 (1.13, 1.85)**

All significant findings in the table are bold.

For peer review only

eTable 6 Associations between $PM_{2.5}$ and NO_2 exposure in different pregnancy periods and adverse birth outcomes (co-exposure models)

Per IQR increase in		Mean difference		Odd ratios			
		Birth weight, grams (95% CI) (N=941)	Birth length, cm (95% CI) (N=927)	PTB (case: 33) (95% CI) (N=945)	LBW (case: 30) (95% CI) (N=945)	LGA (case: 108) (95% CI) (N=945)	SGA (case: 84) (95% CI) (N=945)
Estimated exposure to $PM_{2.5}$	90 days prior to conception	75.00 (-9.86, 159.86)	0.23 (-0.14, 0.60)	0.98 (0.56, 1.89)	0.41 (0.14, 1.22)	1.14 (0.55, 2.40)	1.18 (0.48, 2.92)
	First trimester	19.59 (-71.23, 110.41)	0.00 (-0.40, 0.39)	0.97 (0.26, 3.65)	0.66 (0.14, 3.05)	0.67 (0.32, 1.42)	0.73 (0.28, 1.93)
	Second trimester	-25.62 (-104.32, 53.09)	0.08 (-0.26, 0.42)	1.34 (0.37, 4.86)	0.94 (0.21, 4.21)	0.83 (0.42, 1.62)	0.69 (0.34, 1.40)
	Third trimester	13.77 (-72.33, 99.86)	-0.2 (-0.58, 0.17)	1.12 (0.31, 4.07)	0.94 (0.26, 3.35)	1.00 (0.48, 2.12)	0.57 (0.26, 1.23)
	Total pregnancy	21.13 (-36.41, 78.67)	0.02 (-0.23, 0.27)	0.73 (0.33, 1.61)	0.52 (0.24, 1.15)	0.98 (0.60, 1.61)	**0.55 (0.32, 0.96)**
Estimated exposure to NO_2	90 days prior to conception	-18.63 (-64.02, 26.76)	-0.09 (-0.29, 0.10)	1.24 (0.61, 2.49)	1.3 (0.64, 2.64)	1.27 (0.84, 1.90)	1.39 (0.90, 2.15)
	First trimester	-14.53 (-61.15, 32.09)	0.05 (-0.16, 0.25)	0.91 (0.45, 1.83)	1.14 (0.55, 2.37)	1.33 (0.89, 2.00)	**1.70 (1.07, 2.69)**
	Second trimester	-14.46 (-57.45, 28.54)	-0.08 (-0.26, 0.11)	1.22 (0.63, 2.36)	1.36 (0.68, 2.71)	1.27 (0.87, 1.87)	1.50 (1.00, 2.24)
	Third trimester	-13.13 (-64.87, 38.62)	0.04 (-0.18, 0.27)	0.77 (0.35, 1.67)	0.97 (0.43, 2.16)	1.41 (0.90, 2.23)	**1.77 (1.08, 2.91)**
	Total pregnancy	-15.02 (-49.33, 19.30)	0.00 (-0.15, 0.15)	1.08 (0.64, 1.80)	1.28 (0.75, 2.18)	1.21 (0.90, 1.63)	**1.60 (1.15, 2.23)**

All significant findings in the table are bold.

Models adjusted for maternal age at enrolment, infant’s sex, maternal BMI at 11–14 weeks' gestation, primiparity, monthly household income level, and season of births, and adjusted for the other air pollutant

For peer review only
